# Identification and spatio-temporal tracking of ubiquitous phage families in the human microbiome

**DOI:** 10.3389/frmbi.2022.1097124

**Published:** 2023-02-14

**Authors:** Arbel D. Tadmor, Gita Mahmoudabadi, Helen B. Foley, Rob Phillips

**Affiliations:** ^1^ TRON - Translational Oncology at the University Medical Center of the Johannes Gutenberg University Mainz, Mainz, Germany; ^2^ Department of Biochemistry and Molecular Biophysics, California Institute of Technology, Pasadena, CA, United States; ^3^ Department of Bioengineering, California Institute of Technology, Pasadena, CA, United States; ^4^ Department of Bioengineering, Stanford University, Stanford, CA, United States; ^5^ Department of Preventive Medicine, USC Keck School of Medicine, Los Angeles, CA, United States; ^6^ Department of Applied Physics, California Institute of Technology, Pasadena, CA, United States; ^7^ Department of Physics, California Institute of Technology, Pasadena, CA, United States; ^8^ Division of Biology and Biological Engineering, California Institute of Technology, Pasadena, CA, United States

**Keywords:** core human virome, human phage markers, phageome, human microbiome, oral virome, metagenome clustering, metagenomic clustering by reference library, MCRL

## Abstract

Viruses are a major component of the human microbiome, yet their diversity, lifestyles, spatiotemporal dynamics, and functional impact are not well understood. Elucidating the ecology of human associated phages may have a major impact on human health due to the potential ability of phages to modulate the abundance and phenotype of commensal bacteria. Analyzing 690 Human Microbiome Project metagenomes from 103 subjects sampled across up to 18 habitats, we found that despite the great interpersonal diversity observed among human viromes, humans harbor distinct phage families characterized by their shared conserved hallmark genes known as large terminase subunit (TerL) genes. Phylogenetic analysis of these phage families revealed that different habitats in the oral cavity and gut have unique phage community structures. Over a ~7-month timescale most of these phage families persisted in the oral cavity and gut, however, presence in certain oral habitats appeared to be transitory, possibly due to host migration within the oral cavity. Interestingly, certain phage families were found to be highly correlated with pathogenic, carriage and disease-related isolates, and may potentially serve as novel biomarkers for disease. Our findings shed new light on the core human virome and offer a metagenomic-independent way to probe the core virome using widely shared conserved phage markers.

## Introduction

Bacteriophages are a major component of the human microbiome, with saliva, for example, containing 10^8^ virus-like particles per milliliter (Pride et al., 2012), and stool containing up to 10^9^ virus-like particles per gram (Reyes et al., 2012). Viruses are also frequently encountered as prophages, with an estimated ~60% of sequenced bacterial genomes predicted to encode at least one integrated phage genetic element (Casjens, 2003; Edwards and Rohwer, 2005). The degree to which these pervasive phage genetic elements modulate the abundance and phenotype of commensal microbiota and impact human health is currently unknown. Phages, for example, have been shown to promote pathogenicity in bacteria, confer antibiotic resistance to hosts, and transduce genes that alter host fitness (Waldor and Mekalanos, 1996; Brüssow et al., 2004; Willner et al., 2011; Pride et al., 2012; Quirós et al., 2014; Navarro and Muniesa, 2017). Furthermore, commensal phages have been correlated with various medical conditions such as type I diabetes, chronic infection, and inflammatory bowel disease (Zhao et al., 2017; Łusiak-Szelachowska et al., 2017; Secor et al., 2017). Phages may therefore potentially have a significant impact on human health.

Despite the abundance of phages in human microbial habitats and their postulated impact on human health, we have a very limited understanding of phage ecology in the human body, in particular the identity of their hosts, their lifestyles, their spatial distribution, their temporal dynamics, and their potential role in mediating disease. Applying standard metagenomic approaches to address such questions is challenging in part because of the staggering genomic diversity that is a hallmark of viruses (Paez-Espino et al., 2016a) and the fundamental plasticity of viral genomes, making it difficult to target and precisely track in space and time specific phage families. Indeed, with few exceptions (Stern et al., 2012; Manrique et al., 2016), previous metagenomic studies have largely focused on the heterogeneity of human viromes (Reyes et al., 2010; Minot et al., 2011; Pride et al., 2012; Reyes et al., 2012; Moreno-Gallego et al., 2019; Shkoporov et al., 2019; Gregory et al., 2020; Zuo et al., 2020; Garmaeva et al., 2021). Conversely, traditional methods that are based on targeting universally conserved genes such as the small subunit ribosomal RNA (SSU rRNA) gene for mapping microbial diversity are not applicable to phages because there is no analogous universally conserved gene in viruses (Rohwer and Edwards, 2002; Edwards and Rohwer, 2005).

In this study we aimed to combine the benefits of metagenomic and targeted sequencing approaches to discover phage families that may be widely present in the human virome. We were motivated by the hypothesis that - in analogy to the SSU rRNA marker - there would be core phage families (whether lytic or lysogenic) that could be represented and identified by conserved marker sequences. If we could find such markers, then in analogy to phylogenetic profiling of SSU rRNA markers, we could use phylogenetic analysis to explore intra-family sequence diversity and track such members across different body habitats, different subjects, and different time points. In this context, we use the term “family” to informally denote phages that have a high degree of sequence similarity across a shared marker gene, and within each family, we use the term “sublineage” to denote members that are more phylogenetically similar based on their shared marker gene. As such, in our framework, we do not necessarily expect that members of the same phage family share homology or similarity across their entire genomes.

We chose to focus our search for phage markers on the large terminase (TerL) subunit, one of the most powerful molecular machines in nature (Sun et al., 2008), a component of the DNA packaging and cleaving mechanism present in numerous double stranded DNA (dsDNA) phages (Rao and Feiss, 2008) and considered to be an important signature of dsDNA phage genomes (Casjens, 2003). Typically, TerL genes of different phages exhibit little overall sequence similarity (Eppler et al., 1991; Chai et al., 1992; Moore and Prevelige, 2002; Rao and Feiss, 2008) and contain only a handful of conserved functional amino acid residues (Rao and Feiss, 2008). However, we previously found that in the case of termites, the hindgut microbiomes of numerous termite species from different parts of the globe shared a certain TerL gene family that was conserved across most amino acid residues enabling us to construct a universal phage marker for this family of phages in termites (Tadmor et al., 2011). Therefore, while the TerL gene in and of itself is not universally conserved and therefore cannot serve as a general purpose universal marker for phages, our finding raised the possibility that other TerL gene families may exist in other species that are conserved and widely shared across members of those species, including humans. Adopting this marker-based approach to the human virome, we were indeed able to identify a set of unrelated TerL-based phage families that are ubiquitously shared across humans. Within each family, phylogenetic analysis enabled us to map with high resolution sublineages across different subjects, body habitats and time points (for an overview of our methodology see **Figure S1**).

## Materials and methods

### Sample collection

Samples from nine orally healthy adults were kindly donated to us by Bik et al. who had collected these samples through a collaboration with a dentist and in accordance to the Stanford IRB protocols (Bik et al., 2010). For each subject, oral biofilm samples were collected from six oral sites using sterile curettes. These oral sites include the tongue ventral, tongue dorsum, buccal mucosa, sub-gingiva, supra-gingiva, and the hard palate. Upon collection, the samples were deposited in PBS buffer. For the viral fraction experiments, additional tongue dorsum samples were collected from a tenth subject that refrained from brushing their teeth or tongue for a minimum of 8 hours prior to sample collection to allow for a substantial buildup of plaque on the tongue dorsum. The samples were collected wearing gloves with a tongue scraper and deposited into a sterile collection tube. Exclusion criteria included: antibiotic use in the preceding three months, active cavities, or gum disease. Sample collection and processing protocols were approved by Caltech Institutional Review Board (IRB protocol 14-0430) and Institutional Biosafety Committee (IBC protocol 13-198).

### Datasets analyzed

**Figure 1:**
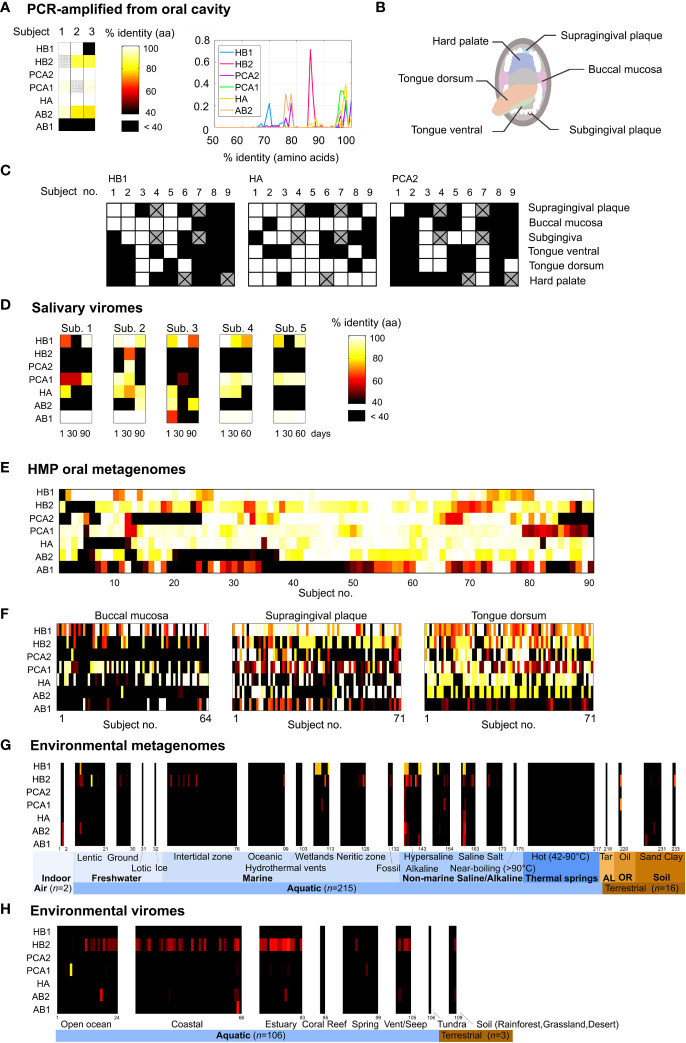
Prevalence of the TerL phage families in the human oral cavity and in natural environments. **(A)** Percent identity between the TerL markers and PCR-amplified TerL sequences obtained from the tongue dorsum, subgingival plaque and supragingival plaque of three orally healthy subjects (unless otherwise stated, percent identities in this study were calculated based on amino acid alignments). The heat map indicates the maximum percent identity across all PCR-amplified sequences. Striped cells indicate that the expected PCR band was present but sequencing failed. **(B)** Oral habitats analyzed by targeted sequencing. **(C)** Percent identity between the TerL markers and PCR-amplified TerL sequences across the oral habitats indicated in (B). Crossed out cells correspond to samples that were unavailable for testing. **(D)** Presence of the TerL phage families in salivary viromes obtained from five periodontally healthy subjects over a 60- to 90-day period (Pride et al., 2012). Heat map applies to panels d-h and shows the maximum percent identity across all BLAST alignments exceeding a predetermined optimal alignment length threshold (**Supporting Text S4**). **(E)** Prevalence of the TerL phage families across 90 subjects based on 382 HMP oral metagenomes regardless of collection site, visit number, or replicate. **(F)** Prevalence of the TerL phage families across 206 HMP oral metagenomes corresponding to three oral habitats, taking into account one metagenome per subject. **(G)** Prevalence of the TerL phage families across 233 metagenomes from natural environments. AL, asphalt lakes; OR, oil reservoir. **(H)** Prevalence of the TerL phage families across 109 viromes of DNA viruses from natural environments.

All metagenomes and viromes analyzed in this study were assembled by the original authors providing those datasets. Apart from the selection pressure analysis, which was performed on nucleotide sequences, analysis was performed on amino acid alignments. The following datasets and databases were analyzed in our study:

(1) The Mira dataset (Belda-Ferre et al., 2012) comprising six metagenomes corresponding to supragingival dental plaque collected from six patients in Spain and divided into three categories based on the number of caries per individual: two individuals who never developed caries in their lives (metagenomes M_HA_, M_HB_), two individuals who had been regularly treated for caries in the past and had a low number of active caries (1 and 4) at the time of sampling of sampling (metagenomes M_PCA_, M_PCB_), and two individuals who had a high number of active caries (8 and 15) and poor oral hygiene (metagenomes M_AA_, M_AB_). In all cases, plaque material from all teeth surfaces was pooled avoiding active cavities if present, and for each of the above six conditions a single metagenome was generated. The mean and median length of contigs in these metagenomes were 336 ± 167 nt (s.d.) and 409 nt, respectively. The mean genome size was 87.7 Mbases. Assembled translated metagenomes can be found on MG-RAST (Glass et al., 2010) with the following IDs: 4447192.3, 4447102.3, 4447103.3, 4447101.3, 4447943.3, 4447903.3.
(2) The Xie dataset (Xie et al., 2010) comprising a metagenome of supragingival and subgingival plaque collected and pooled from eight teeth of a caries-free and periodontally healthy individual from the United States. The mean and median length of contigs in this metagenome were 372 ± 126 nt s.d. and 411 nt, respectively. The genome size was 29.5 Mbases. The assembled translated metagenome can be found on MG-RAST with the ID 4446622.3.
(3) The HMP dataset (Methé et al., 2012) comprising contributions from 103 healthy individuals sampled from up to 15 body habitats, including: attached/keratinized gingiva, buccal mucosa, hard palate, palatine tonsils, saliva, subgingival plaque, supragingival plaque, throat, tongue dorsum, stool, anterior nares, posterior fornix, mid vagina, vaginal introitus, and the retroauricular crease. All subjects were subjugated to rigorous inclusion criteria to control for their health (Aagaard et al., 2013). 748 assembled metagenomes generated in Phase I of the HMP study were subjected to internal quality control assessment based on HMP study guidelines (Methé et al., 2012), remaining with 690 metagenomes that were used in the current analysis (**Table S8**). Metadata from the HMP cohort such as the Medical Record Number (MRN), collection site, visit number, and the replicate number were extracted as previously described (Markowitz et al., 2012). The mean and median length of contigs in HMP metagenomes passing HMP quality control were 582 ± 124 nt (s.d.) and 561 nt, respectively, and for oral metagenomes 529 ± 57 nt (s.d.) and 534 nt, respectively. The HMP metagenomes are available through the IMG/M database.
(4) The Pride dataset (Pride et al., 2012) comprising of viromes extracted from saliva samples of five subjects sampled at day 1, day 30 and day 60 or 90. Subjects were healthy and had not taken antibiotics for at least one year prior to donating samples. All subjects had good oral health based on rigorous inclusion criteria (Pride et al., 2012). The mean and median length of contigs in these metagenome were 328 ± 44 nt (s.d.) and 349 nt, respectively. Assembled translated metagenomes can be found on MG-RAST with the following IDs: 4445735.3, 4446121.3, 4445731.3, 4445728.3, 4446126.3, 4446075.3, 4445734.3, 4445729.3, 4446125.3, 4446124.3, 4445730.3, 4446122.3, 4446120.3, 4445737.3, and 4445736.3.
(5) The MetagenomesOnline (MgOl) portal (Wommack et al., 2012) hosted on the VIROME platform comprising 270 metagenomic libraries, including a large number of viromes. Environmental viromes in **Figure 1H** were selected to match the following filtering criteria: Genesis=natural, Environmental package=all excluding host-associated viromes, and considering only viromes of DNA viruses, resulting in 109 viromes. The mean and median length of contigs in these viromes were 377 ± 70 nt (s.d.) and 362 nt (range 319 – 1362 nt), respectively.
(6) The Human Oral Microbiome Database (HOMD) dataset (Chen et al., 2010) comprising genomes of oral bacteria sequenced either as part of the HOMD project or as part of other sequencing projects, including the HMP study.
(7) NCBI’s non-redundant (nr) protein database, comprising all non-redundant GenBank CDS translations, the protein data bank (PDB), SwissProt, the Protein Information Resource (PIR) database and the Protein Research Foundation (PRF) database, excluding environmental samples from WGS projects.
(8) The IMG/M database (Chen et al., 2018) comprising at the time of analysis 16338 bacterial and archaeal isolates, 475 viral isolates, and 1335 environmental metagenomes. Environmental metagenomes in **Figure 1G** were selected as follows: for each environmental ‘family’ class a maximum of 50 metagenomes were randomly selected, limiting metagenomes to 5 GB due to the downloading limitation of the IMG platform, resulting in 448 metagenomes. Of these, we retained only metagenomes with constructed protein databases and excluded metatranscriptomes. In order for our comparison between HMP oral metagenomes and environmental metagenomes to be unbiased, we further controlled for the average contig length and the total genome size. To control for the average contig length we selected only environmental metagenomes whose average contig length exceeded the minimal contig length of assembled HMP metagenomes (300 bp) (Methé et al., 2012). To control for the genome size, we excluded environmental metagenomes whose genome size was below the minimal genome size of HMP oral metagenomes. Applying these selection criteria resulted in 233 environmental metagenomes analyzed in **Figure 1G**.
(9) The IMG/VR database (Paez-Espino et al., 2016b) (IMG_VR_2018-07-01_4) comprising at the time of analysis viral contigs from 3663 metagenomes available on IMG satisfying the constraint “Ecosystem phylum =Environmental”.
(10) NCBI’s env_nr database containing nearly 10 million proteins sequences from whole genome sequencing (WGS) metagenomic projects.

### DNA extraction

DNA extraction was performed on each sample using the MoBio PowerBiofilm^®^ DNA Isolation Kit, which uses a DNA extraction and purification protocol optimized for biofilms. It combines the benefits of a chemical lysis treatment with the physical forces applied during a bead-beating process. Disposable lab coats and face masks were worn at all times.

#### Degenerate primer design

Degenerate primers for the TerL markers were designed based on sequences obtained from the HMP dataset, the Xie dataset, the Mira dataset and HOMD as follows: candidate 3’ positions for primers were chosen when possible at positions achieving a bit score of at least 3.5 when RPS-BLASTing the amino acid sequence of the given TerL marker against the Conserved Domains Database (CDD) (Marchler-Bauer et al., 2016). Primers were then selected in regions spanned by all datasets, requiring that the percent identity of the majority consensus amino acid residue, when equally weighted across all datasets, was at least 90% while limiting the degeneracy of each primer to 64 fold. Primer sequences were then designed using the CODEHOP algorithm (Rose et al., 1998), with the core region maximally degenerate based on the genetic code, and the consensus clamp region chosen to match the codon bias present in the alignments. Primer nucleotide sequences were optimized to have a GC clamp at the 3’ end, minimize homodimers, heterodimers and hairpins, and have a melting temperature of 60°C. Degenerate primer sequences and targeted conserved amino acid motifs are provided in **Table S3**.

### PCR preparation

PCR reactions using the degenerate primers described above were performed in a laminar flowhood. Each PCR reaction contained 10.5 µL of RT-PCR Grade Water (Ambion^®^), 1 µL of extracted DNA at 1 ng/µL, a premix containing AccuStart™ Taq DNA polymerase, dNTPs, and MgCl_2_, and 0.5 µL of reverse and 0.5 µL of forward primers (at 50 ng/µL). A higher than recommended concentration was used since the primes are 32-64 fold degenerate. For MiSeq sequencing, primers were barcoded using error-detecting barcodes (appended onto the forward primer sequence) and synthesized by IDT (Hamady et al., 2008). For each extraction protocol, we performed three negative controls that instead of biofilm sample contained RT-PCR Grade Water (Ambion^®^), free of any DNAase and RNAse. These three extraction controls along with five no template controls were used during each PCR session to ensure there is no contamination being introduced during either process. Disposable lab coats and face masks were worn at all times. After each session all surfaces were cleaned with DNA AWAY™ and 95% ethanol. The flowhood interior surfaces and the equipment inside were exposed to UV for one hour at the end of each session. The following PCR thermocycling protocol was used in accordance to PerfeCTa qPCR SuperMix recommendations: 1) 10-minute activation of AccuStart™ Taq DNA polymerase at 95°C, 2) 10 seconds of DNA denaturation at 95°C, 3) 20 seconds of annealing at 60°C, 4) 30 seconds of extension at 72°C, 40 cycles repeating steps 2 to 4, followed by 5 minutes of final extension at 72°C.

### Gel electrophoresis and PCR cleanup

2% agarose in TAE buffer was used to cast the gels. 5 µL of PCR reaction was mixed with 1 µL of 6X loading dye and set to run for 30 min at 100V. PCR products were purified using the QIAquick PCR Purification Kit from QIAGEN in accordance to their manual.

### Sequencing and sequence analysis

Double-stranded DNA concentration in PCR-purified products was measured and standardized using the Qubit instrument. Sequences amplified for the AB2, HB2 and PCA1 markers were sent for Sanger sequencing following the IDT standard protocol. Sequences amplified for the HB1, HA and PCA2 markers were sent for MiSeq sequencing. Because each sample for MiSeq sequencing was barcoded during the PCR reaction, the samples were mixed into one vial and sent to GENEWIZ, Inc for library preparation and Illumina MiSeq sequencing (2 × 300bp Paired-End sequencing). *join_paired_ends.py* script from the Quantitative Insights Into Microbial Ecology (QIIME) package (Caporaso et al., 2010) was used to join paired-end reads. We then performed several quality control steps to eliminate any sequences that arose due to sequencing error. Paired reads that had any mismatches across their overlapping bases were eliminated. The overlap between the paired reads constituted the entire length of the sequence. Using an in-house script developed for this project, *seqQualityFilters.py*, we then eliminated sequences with any bases with Phred scores of 29 or below (excluded from this step were the first and last two bases which are generally associated with low Phred scores for all sequences). Using the same in-house script (i) sequences were assigned to their respective TerL markers based on their primer sequences; (ii) sequences with incorrect barcode lengths or incorrect primer sequences were eliminated; (iii) the primer and barcode sequences were removed and the barcode sequences were written to a separate file for a later step; (iv) sequences with incorrect lengths were removed. *split_libraries_fastq.py* from QIIME was used to demultiplex the reads based on their barcode sequence, while further eliminating reads with any errors in their barcodes. MiSeq sequences analyzed in **Figure 1A** and **Table S5** were clustered using QIIME’s *pick_otus.py* script, based on their sequence similarity into operational taxonomic units (OTUs) (Edgar, 2010) using an OTU cutoff of 95% for HA and PCA2, and 98% for HB1.

### Viral fraction protocol

To test if oral phages carrying close homologs of HB1 are lytic we tested the bacterial and viral fractions derived from an oral sample for the presence of the HB1 marker. Saliva samples were defrosted from storage at -20°C. Samples and an extraction control were vortexed for 2 minutes at half-speed, followed by centrifugation at 8000g for 10 minutes. The supernatant was removed to a fresh tube and the pellet was resuspended in sterile filtered PBS. Supernatant and pellet were re-centrifuged (8000g, 5 minutes). 200µL of the original supernatant (putative viral fraction, VF) were filtered through a PBS-rinsed 0.2µm 13mm tuffryn filter. Original pellet (putative bacterial fraction, BF) was rinsed and resuspended 200µL PBS. BF and VF, as well as extraction controls, were extracted according to standard protocol with PowerBiofilm DNA Isolation Kit (MoBio). TerL markers HB1 and HA were amplified as described above. Markers were amplified from 1 µL template using 0.8 µL of 10 micromolar forward and reverse primers, with PerfeCTa MasterMix. Marker HB1 was also amplified using 2 µL of template and 0.8 µL of 100µM primers. PCR products were assayed for presence or absence on 2% agarose gel (**Figure S7**). Six replicates of the same VF extract were amplified to test for low-copy templates in the viral fraction.

### Identifying shared TerL markers in the human oral cavity

#### Identifying viral gene families in the Mira metagenomes

To identify TerL markers core to the human oral cavity we focused our analysis on the six plaque metagenomes from the Mira dataset reflecting human subjects with varying degrees of oral hygiene. We applied to each of these metagenomes a clustering algorithm called Metagenomic Clustering by Reference Library (MCRL) that was developed by the current authors (Tadmor and Phillips, 2022). Briefly, MCRL uses a reference library containing a set of reference sequences (in this case the viral RefSeq database v48 (Pruitt et al., 2007) containing ~97,000 viral genes) to initially identify and retain all reference sequences that have putative homologs in the given input metagenome. MCRL then proceeds to apply an iterative greedy clustering algorithm to the list of retained reference sequences and, upon convergence, reports the subset of reference sequences that are homologous to minimally overlapping sets of contigs in the metagenome. Thus, the final output of MCRL is the list of reference sequences with putative homologs in the input metagenome that have minimally overlapping “signatures” in the metagenome. A “signature” of a reference sequence in a metagenome is the list of contigs in the metagenome yielding an E value below 0.001 when BLASTing the amino acid sequence of reference sequence against the translated metagenome. Reference sequences reported by MCRL therefore reflect potential different and unrelated gene families present in the metagenome.

To maximize detection sensitivity, we applied MCRL using its default parameters and a “stringent overlap” condition wherein two reference sequences are determined to overlap if their signatures overlap by more than 50% regardless of the reference sequence. In terms of sensitivity, we have previously shown that when using a stringent overlap condition MCRL achieves a sensitivity of at least 95% for detecting TerL gene families that exhibit up to 30% divergence compared to the viral RefSeq database, and overall has better sensitivity compared to conventional metagenomic clustering methods (Tadmor and Phillips, 2022). A detailed discussion of MCRL’s default parameters, robustness to changes in parameters or presence of noise, and a benchmark comparing MCRL to standard metagenomic clustering methods in terms of sensitivity and accuracy is provided in (Tadmor and Phillips, 2022). When applying MCRL to each of the six Mira metagenomes, analyzing in total 1.75 × 10^6^ translated contigs, MCRL reported in total 7411 viral RefSeq genes (as depicted in **Figure S2A**).

### Screening for shared TerL sequences

To enrich for TerL candidates with significant putative homologs in the metagenomes and to remove spurious solutions, we retained from the list of 7411 viral RefSeq genes reported by MCRL a total of 76 reference genes encoding TerL genes (based on the RefSeq annotation provided by MCRL) that have a signature size of 5 or higher and that share at least 10% identical amino acid residues when aligned against their representative contig (the representative contig of a reference sequence is the contig yielding the lowest E value when BLASTing that reference sequence against the metagenome).

To enrich for closely related TerL lineages that are potentially shared across humans we BLASTed the amino acid sequence of the representative contig corresponding to each of the 76 homologous TerL RefSeq genes identified by MCRL in the Mira dataset against the translated oral metagenome from the Xie study - an oral metagenome of a healthy individual from a different continent participating in an independent study – and retained only candidates that yielded at least 75% identity at the amino acid level. A 75% identity threshold was empirically motivated based on our previous experimental results in the termite hindgut system (Tadmor et al., 2011) where we found that the universally shared TerL lineage in this ecosystem exhibited 81.1% ± 7.8 identity at the amino acid level across different termite species. Indeed, this threshold was justified in retrospect given that the diversity of HMP metagenomic sequences closely related to the markers was captured using a 70% identity threshold at the amino acid level, as shown in **Figure S9** and discussed in **Supporting Text S8**. This final filtering step left us with 11 TerL gene fragments (**Table S2**). We then BLASTed all 11 TerL gene fragments against each other at the protein level and removed redundant sequences, leaving us with seven non-homologous independent candidates for shared TerL markers (**Table S3**).

### Obtaining full-length TerL markers

Since the metagenomes used to obtain the TerL marker candidates have relatively short contigs (with a mean contig length of 336 nt), the seven candidate TerL markers identified in the Mira dataset span only a fragment of the TerL gene length, which spans on average 1650 nt. To obtain shared TerL markers that span the entire length of a TerL gene we collected and aligned for each of the seven TerL candidate markers closely related amino acid sequences from the Xie, Mira, HOMD and the HMP datasets yielding at least 70% identity at the amino acid level. For each of the seven alignments we then selected the sequence that maximized the average percent identity across all other sequences (applying equal weights to each database), penalizing shorter sequences by setting the alignment score in positions containing gaps to 0. In this manner, we identified for each of the seven TerL candidates a closely related sequence spanning the entire length of the TerL gene. Contigs carrying the full-length TerL genes are listed in **Table S3** and annotation for these contigs is provided in **Figure S5**.

### BLAST alignments

All BLAST analyses were performed locally using blastp v2.2.22+ with default settings on amino acid alignments. Alignment thresholds are discussed in **Supporting Text S4** and **S8**.

### Collection of TerL marker homologs present in bacterial and phage isolates

To exhaustively identify all close homologs of the TerL markers in bacterial and phage isolates, each of the seven TerL markers were BLASTed against all available genomes on the IMG platform, NCBI’s non-redundant (nr) protein database, and the HOMD database. For our phylogenetic analysis we included all TerL sequences that yielded at least 70% identity at the amino acid level across at least 90% of the TerL marker length, remaining with approximately 2300 hits (**Table S10**).

### Determining health-related status of isolates

Each isolate harboring a close homolog of a TerL marker was assigned a “health-related status” to reflect its pathogenicity or potential association with disease. The decision regarding the health-related status was determined as follows: when information about the pathogenicity of the isolate or details about the bacterium’s isolation were provided in IMG annotation or in annotation from another public database this information was used to determine the health-related status of the isolate. When public annotation was not available or not sufficiently detailed, original publications describing the isolation of the bacterium were sought. When the information provided in the original publication was not sufficiently detailed, the original authors were consulted. Based on the above information, the health-related status isolates was assigned to one of the following categories: “P”=the bacterial isolate/strain was designated as a pathogen by the author and/or the bacterium was isolated from a sick individual with a diagnosed disease or from a diseased organ, a diseased body site, a sterile body site, or a diseased animal. Sterile body sites include, for example, blood, cerebral spinal fluid, lymph nodes, peritoneal fluid, synovial fluid, and internal organs. “C”=the bacterial isolate was designated as a carriage strain by the author. “H”=the bacterial isolate/strain is not considered to be pathogenic by the author and/or was isolated from a healthy subject, healthy tissue or a healthy animal. When the required information was insufficient or unavailable to determine the health-related status of the isolate, the health-related status was designated “n.a.”. In case of phage isolates, the health-related status pertains to the bacterium strain from which the phage was induced. The health-related status for all isolates is provided in **Table S10** along with appropriate references.

### Phylogenetic analysis

Phylogenetic analysis was performed on translated TerL sequences obtained from all 690 HMP metagenomes passing HMP quality control criteria as well as all bacterial and phage isolates harboring close homologs of the markers listed in **Table S10**, taking one representative per OTU as described below (OTU assignment for all isolates is provided in **Table S10**). Phylogenetic analysis was performed based on sequence alignments spanning at least 400 amino acids and yielding at least 70% identity at the amino acid level compared to the TerL markers, resulting in alignments spanning on average 69.2% of the TerL gene length (range: 62.7% to 88.9%). In the case of human bacterial isolates, one representative strain was selected per species per body region and per given health-related status, using a 3% OTU threshold at the amino acid level with alignments spanning at least 98% of the TerL marker length. For non-human bacterial isolates, one representative strain per species was selected. Translated nucleotide sequences were then aligned with MUSCLE (Edgar, 2004) in MEGA (Tamura et al., 2013). The optimal amino acid substitution model was estimated with ProtTest3.4 (Darriba et al., 2011) using the AIC criterion allowing for 48 model combinations permitted in SplitsTree4 (Huson and Bryant, 2006) with +G and +I options (amino acid frequencies are hard-coded in SplitsTree4). Models tested include: WAG (Whelan and Goldman, 2001), JTT (Jones et al., 1992), mtREV (Adachi and Hasegawa, 1996), mtMam (Cao et al., 1998), Dayhoff (Dayhoff and Schwartz, 1978), CpREV (Adachi et al., 2000). Optimal model-averaged parameters using Akaike weights were estimated with ProtTest3.4 for the shape parameter of the gamma distribution (α), and the proportion of invariant sites (Pinv). Neighbor-Net networks were estimated with SplitsTree4 (Huson and Bryant, 2006) based on amino acid sequence alignments using maximum likelihood distances estimated with optimal model-averaged parameters.

### Selection pressure analysis

Selection pressure analysis was performed using codeml codon models included in the PAML package (Yang, 2007). Sequence alignments were generated using Geneious global alignment with free end gaps with default gap open and gap extension penalties, using an identity cost matrix (Kearse et al., 2012). Phylogenetic trees were created using SeaView GTR model with default parameters (Gouy et al., 2009). We tested NSsite models with different number of site classes: M0 (one site class with constant ω, where ω = dN/dS), M1a (two site classes: ω=1, ω<1) and M2a (three site classes: ω=1, ω<1, ω>1). The CodonFreq parameter was set to F3x4. Models M0 and M1a were compared against each other as were M1a and M2a. The models were compared using the likelihood ratio test and the statistical significance of the outcome was determined based on the chi-squared distribution (Yang, 2007).

## Results and discussion

### Hunting for shared phage families in the human oral virome

The habitat we chose to begin our search for ubiquitous phage families in humans was the oral cavity due to its rich microbial diversity (Huttenhower et al., 2012), presence of many unique niches that can be explored, and its relevance to human health as a gateway to the human body (Li et al., 2000). The most straightforward way to find a TerL marker core to the human oral virome would be to perform a joint phylogenetic analysis of all TerL sequences across multiple oral metagenomes obtained from different individuals. Such an approach, however, is impractical due to the highly divergent nature of TerL sequences, the relatively short lengths of contigs, and limitations of metagenomic annotation (**Supporting Text S1**). To circumvent these challenges we devised a method based on a combination of clustering and filtering steps. To this end, we applied a novel metagenomic clustering method that we developed that uses a reference library of annotated viral sequences to extract putative unrelated viral gene families from a metagenome (Tadmor and Phillips, 2022) (see Materials and Methods). This approach enabled us to examine the putative viral gene families present in six metagenomes of supragingival dental plaque samples obtained from six individuals from Spain with varying degrees of oral hygiene (Belda-Ferre et al., 2012), referred to as the Mira dataset (see **Figure S2A** and the Materials and Methods section for a summary of our search strategy). Analzying in total nearly two million contigs, our search algorithm identified an average of 1236 viral gene families per metagenome (**Table S1**), of which 76 encoded TerL genes (**Table S2**). Since our goal was to establish whether the majority of healthy humans share certain conserved phage markers, we narrowed the list of TerL candidates to those that were conserved across the majority of the TerL gene in at least two human subjects from two independent studies from different parts of the world. The second study we selected, which we refer to as the Xie dataset, was obtained from the oral cavity a healthy individual from the United States (Xie et al., 2010). This final screening step left us with seven non-homologous TerL gene fragments labeled *H*A, *H*B1, *H*B2, *PC*A2, *PC*A1, *A*B1, *A*B2, with the prefix corresponding to the oral health of the subject in which the marker discovered, indicating good (*H*), mediocre (*PC*), or poor (*A*) oral hygiene (**Figure S2**). Such a labeling scheme enabled us to correlate marker prevalence with oral hygiene (see below). Lastly, each TerL gene fragment was swapped with a closely related homologous full-length TerL sequence, using the Human Oral Microbiome Database (HOMD) (Chen et al., 2010) and the Human Microbiome Project (HMP) dataset (Methé et al., 2012) to expand our sequence search space to include full length sequences (see Material and Methods for objective search strategy). The HMP dataset was excluded from the step of identifying shared phage markers in order to avoid introduction of biases in subsequent analyses of this dataset.

Our full-length phage markers corresponded to HK97-associated COG4626/pfam03354 Terminase_1 (HA, HB1, HB2, PCA2, AB1), and SPP1-associated COG1783/pfam04466 Terminase_3 (PCA1), with AB2 not corresponding to any known pfam/COG (**Table S3**). These results were consistent with phylogenetic analysis of the TerL markers in the broader context of TerL genes observed in nature (**Figure S4**). The seven full-length TerL marker genes we obtained represent unrelated lineages since any pair of TerL markers exhibited little or no sequence similarity at the amino acid level (**Table S4**), as is typically the case for TerL genes. Going back to the Mira study, we BLASTed the full-length TerL markers against the six oral metagenomes and found that apart from PCA2, all markers achieved alignments exceeding 70% identity at the amino acid level in 3 to 5 of the six subjects, confirming the shared presence of these markers in this small cohort (**Figure S2B**).

### Experimental validation of phage families derived bioinformatically from metagenomic datasets

To confirm that our bioinformatically-derived TerL-based phage families can also be verified experimentally we tested for the presence of TerL markers in oral samples collected from orally healthy subjects using targeted sequencing. Using amino acid alignments from multiple public datasets we designed degenerate primers (Rose et al., 1998) targeting conserved amino acid motifs (**Table S3, Figure S3**). Sequencing the resulting PCR products, we were indeed able to experimentally identify the presence of all but one (AB1) of the phage families in at least two of the three tested individuals (**Figure 1A**, **Table S5**). Using the same targeted sequencing approach we then tested for the presence of three of the phage families (HB1, HA, and PCA2) across six oral habitats collected from nine additional subjects (**Figure 1B**). We found all three phage families in this cohort were robustly present in the oral cavity (**Figure 1C**). In a companion paper we discuss in greater depth TerL sequence diversity obtained by targeted sequencing, including HB1 sequences obtained from 61 individuals across three continents (Mahmoudabadi et al., 2019).

### Evidence for the functionality of sequences retrieved by the phage markers

Although whole community metagenomes provide a snapshot into both lytic and lysogenic phage families, it has the drawback that it does not provide direct evidence that the sequences we recover are part of functional phages. However, several indirect lines of evidence suggest that the shared TerL lineages we identified encode functional genes associated with genuine phage elements. First, we confirmed that the original contigs encoding the TerL markers harbored larger phage-like elements (**Figure S5**), and that close homologs of most of the markers can be found in extended prophage-like elements (**Figure S6, Supporting Text S2**), helping to rule out non-genuine phage elements such as gene transfer agents (GTAs) and bacteriocins (**Supporting Text S3**). Second, we confirmed that sequences retrieved using the markers or primers were under substantial negative selection (**Table S6**), lacked premature stop codons or frameshift mutations and functional signatures typical of TerL genes were strictly conserved in these sequences (see **Figure S3** for alignments and **Table S7** for a summary of conserved functional signatures). Finally, we showed that the markers can be detected in virus-like particles (VLPs) using a fourth metagenomic dataset comprising 15 salivary viromes obtained from five periodontally healthy human subjects (Pride et al., 2012) (**Figure 1D**). In the case of HB1, we further experimentally verified these results by showing that this marker could be detected by PCR amplification in virus-like particles extracted from a tenth oral sample from our own cohort of oral samples (**Figure S7A**). Taken together, the evidence above suggests that, overall, TerL sequences retrieved using our markers encode functional genes that have either been active in recent evolutionary history and/or are part of a population of functional phages, and thus we speculate are not degenerating pseudogenes experiencing random drift (**Supporting Text S3**).

### Prevalence of the phage markers in the HMP oral metagenomes

**Figure 2:**
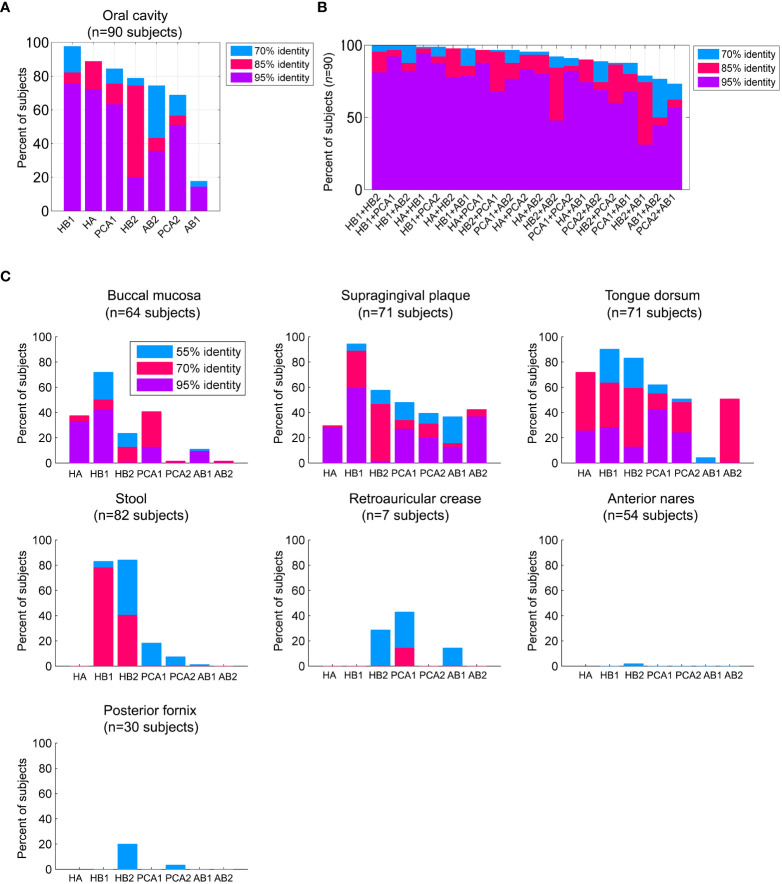
Prevalence of the TerL phage families across human habitats. **(A)** Percent of subjects that were positive for the TerL phage families in the oral cavity regardless of collection site, visit number, or replicate evaluated across 90 subjects based on 382 HMP oral metagenomes passing HMP quality control criteria. For all panels, a TerL phage family was considered present in a subject if the maximum percent identity of its TerL sequence across all BLAST alignments spanning at least 150 amino acids exceeded the indicated percent identity threshold. **(B)** Percent of subjects positive for any pair combination of TerL phage families. **(C)** Presence of TerL phage families across seven body habitats taking into account one metagenome per subject. Alignments in all panels were performed on amino acid sequences.

We next explored the prevalence of these phage families within the HMP oral cohort, which comprises 90 subjects sampled from up to eight oral sites spanning in total 382 metagenomes (**Table S8**). We found that remarkably virtually all 90 subjects were positive for the HB1 phage family with at least 70% identity, and 76% of subjects were positive for the HB1 phage family with at least 95% identity (see **Figures 1E**, **2A**, for alignment criteria see **Supporting Text S4**). Likewise, more than 85% of subjects were positive for the HA and PCA1 phage families with at least 70% identity at the amino acid level, and 72% and 63% of subjects were positive for the HA and PCA1 phage families, respectively, with at least 95% identity (**Figure 2A**). In addition, nearly all subjects were positive for any pair combination of HB1, HB2, HA and PCA1 (**Figure 2B**), however, presence of any specific pair of phage families was only weakly correlated (absolute Spearman’s rank correlation ≤0.24), consistent with these markers representing independent TerL phage families. Since all subjects participating in the HMP study were orally healthy, perhaps expectedly, we found that markers obtained from metagenomes of orally healthy subjects in the Mira dataset (HB1, HB2, HA) were more prevalent than markers obtained from metagenomes of subjects with oral health problems (**Supporting Text S5**). Given the high prevalence of TerL phage families in the HMP, Mira, and Xie oral metagenomes, the salivary VLP metagenomes, and our own oral cohort interrogated by targeted sequencing suggests that these TerL phage families are ubiquitous in humans and contribute to a widely shared human virome. In **Supporting Text S2** we summarize the requirements we propose a ubiquitous viral marker should satisfy.

### Prevalence of the phage families in natural environments

To check whether the TerL phage families that we identified are in fact specific to the human virome or also prevalent in natural environments, we compared the prevalence of the TerL phage families across three oral habitats (206 metagenomes) with their prevalence across 233 environmental metagenomes from the IMG/M database (Chen et al., 2018) collected from over 70 unique sites across 13 countries, selected to have comparable genome sizes (number of assembled coding contigs) and contig lengths to HMP oral metagenomes (**Table S9**). Our comparison indicates that members of the TerL phage families were mostly prevalent in human oral metagenomes and relatively sparse in environmental metagenomes, with most markers, except for HB1 and to a lesser extent HB2, displaying relatively remote homologs in a small subset of environmental metagenomes (**Figures 1F, G**). In **Supporting Text S6** we show that members of the HB1 and HB2 phage families appearing in environmental samples are phylogenetically distinct from their respective human-associated counterparts. To rule out potential sampling bias, we repeated this analysis in 3663 environmental metagenomes from the IMG/VR database spanning 35 distinct ecosystems (listed in **Table S9**), comprising in total nearly 20 million viral contigs (Paez-Espino et al., 2016b). Indeed, this analysis revealed a similar pattern of prevalence, as shown in **Figure S8**. An analysis of 109 environmental viromes (metagenomes of VLPs) deposited in the VIROME portal (Wommack et al., 2012) also revealed similar patterns (**Table S9** and **Figure 1H**). In addition, we confirmed that the env_nr database did not contain more divergent homologs when using PSI-BLAST, and ruled out potential biases related to contig length, genome size, community complexity, read depth, method of assembly and sequencing technology (**Supporting Text S7**). Lastly, we performed an exhaustive search for TerL phage families in bacterial and viral genomes deposited in the IMG/M and non-redundant (nr) protein databases (Pruitt et al., 2007). Consistent with our analyses of whole community and VLP metagenomes, we found that except for six genomes originating from environmental bacteria that were positive for HB1, and two genomes positive for HB2 isolated from sewage and industrial environments, all remaining ~2300 genomes were obtained from bacteria isolated from human, animal, or insect (HB1) hosts (**Table S10**). These results agree with our previous finding and show that aside from HB1 and to a lesser extent HB2, the shared TerL phage families were quite specific to the viromes of humans and animals.

### Distribution of the phage families across the human body

To elucidate the spatial distribution of the TerL phage families across the human body we mapped the presence of members of these families across seven body sites collected from 94 healthy individuals spanning 379 HMP metagenomes. Presence was determined based on a 70% identity threshold because this threshold captured the majority of phage family members (**Figure S9**), however, our findings did not depend on the applied percent identity threshold, as further discussed in **Supporting Text S8**.

We found that most TerL phage families (HA, PCA1, PCA2, AB1, AB2) were indeed prevalent in the oral cavity and generally absent from stool, the nasal cavity, the female urogenital (UG) tract, and skin, except for a mild presence of PCA1 in skin (**Figure 2C**). HB1 and HB2 phage families, however, were exceptional and were found to be widespread not only in the oral cavity, but also in a considerable fraction of stool samples (**Figure 2C**), with up to ~90% and ~60% of subjects containing HB1 and HB2 TerL phage families in stool samples, respectively, when controlling for genome size (**Supporting Text S7**). To confirm the distribution of these phage families in stool samples, we tested for their presence in 14 metagenomic studies investigating stool samples obtained from heathy individuals included in the Gut Virome Database (GVD) (Gregory et al., 2020). We found the HB1 phage family in nearly all studies, including 11 viromes (metagenomes of VLPs), showing that HB1 was present in stool samples of individuals across four continents. With few exceptions, the remaining markers were either not detected in the gut studies, or present only as remote homologs, confirming the distribution we observed in the HMP metagenomes (**Table S11**). HB2 phage family was present in all three whole community studies, and to a lesser extent in viromes. The remaining phage families were largely absent from the gut studies, confirming the spatial patterns of distribution we had observed in the HMP metagenomes (**Table S11**).

We next contrasted our findings in the HMP dataset with the presence of the markers in bacteria and phages isolated from different human body habitats. To this end we exhaustively searched the IMG, HOMD and the non-redundant (nr) protein databases for close homologs of the markers, carefully determining for each isolate its health-related status, for example, was it isolated from a healthy human subject or a human subject diagnosed with a certain disease, was the isolate designated as a human pathogen, a carriage strain, or was the isolate obtained from a non-human host (see Materials and Methods for precise criteria and **Table S10** for a comprehensive list of isolates). Focusing on bacterial isolates obtained from healthy individuals, we indeed found that the HA and PCA1 phage families were present in oral and/or airway bacterial isolates from the *Streptococcus* genus, a genus known to be highly abundant in the oral cavity of healthy humans (Huttenhower et al., 2012). Likewise, AB2 was found in an oral bacterial isolate from the *Actinomyces* genus, a genus also known for its abundance in the oral cavity of healthy humans (Huttenhower et al., 2012) (phylogenetic placement of all bacterial hosts is summarized in **Table S12**). No oral bacterial isolates were found for PCA2, however, PCA2 was found in colon and gastric isolates, the latter suspected to be a swallowed oral bacterium caught in the act of transiting (see **Table S10** for further details).

Finally, members of the HB1 and HB2 phage families were found in multiple gut bacterial isolates from the widespread Firmicutes phylum (Huttenhower et al., 2012), in agreement with our metagenomic analysis. Interestingly, however, no bacterial isolate from the oral cavity or airways, including carriage and pathogenic strains, was found to contain even distant homologs of the HB1 marker despite the overwhelming abundance of HB1 in the oral cavity of healthy humans. One possible explanation for this intriguing result could be that in healthy humans, the HB1 phage family found in the oral cavity is predominately lytic, a prediction that we were able to experimentally confirm, as we further discuss below. Despite the high prevalence of HB1 and HB2 phage families in stool samples, they were not related to the crAss-like phage family (Guerin et al., 2018), a recently identified widespread family of phages in gut viromes.

### Phylogenetic analysis of TerL phage families

Thus far our attention has been focused on the prevalence of each phage family. However, within each family, members display incredible inter-and intra-subject sequence diversity (**Tables S5, S13**). To better characterize this sequence diversity, we wished to understand whether each phage family was comprised of a single indivisible TerL lineage, or, conversely, multiple distinct TerL sublineages, in which case we aimed to determine how different body sites were associated with different sublineages. For our marker-based phylogenetic analysis we chose to use phylogenetic networks (Bryant and Moulton, 2004; Huson and Bryant, 2006) to account for possible viral recombination events, events which cannot be represented by phylogenetic trees (Lemey et al., 2009).

**Figure 3:**
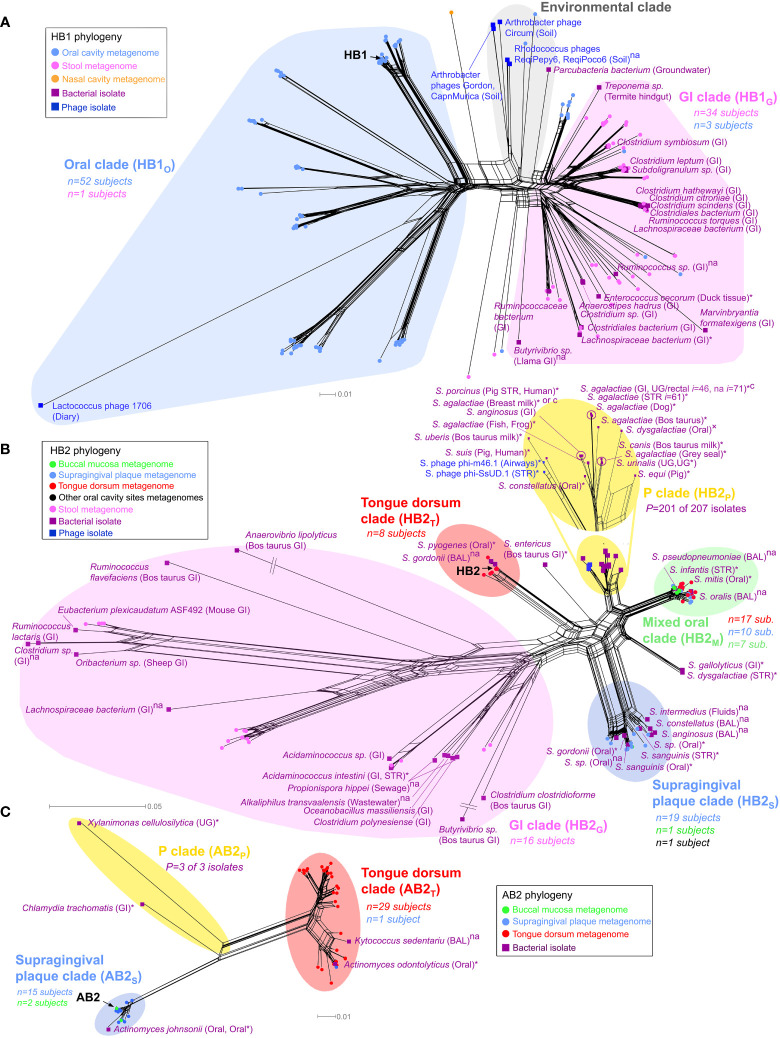
Phylogenetic analysis of TerL phage families. Neighbor-Net analysis for **(A)** HB1, **(B)** HB2 and **(C)** AB2 phage families based on 386, 341, and 350 unambiguous amino acid residues, respectively including sequences obtained from the HMP metagenomes (circular nodes) and sequenced bacterial and phage isolates (square nodes). Pathogenic bacteria, bacteria isolated from diseased body sites, sterile organs, individuals with diagnosed diseases or diseased animals are marked with an asterisk, otherwise “×” denotes suspected pathogenicity, “c” denotes a carriage strain, and “na” denotes unknown health-related status. Bacterial isolates belonging to the same species, sampled from the same body region (mouth, skin, nose, the gastrointestinal (GI) tract or the UG tract), and with the same health-related status were consolidated using a 3% OTU threshold at the amino acid level (OTU assignment for all isolates is provided in **Table S10**). *n* denotes the number of HMP subjects contributing sequences to a given clade color coded by the body habitat indicated in the legend, *P* denotes the total number of disease or carriage associated human bacterial isolates within a “P” clade out of all isolates in the given clade. In the “P” clade of HB2, *i* denotes the total number of human bacterial isolates represented by the given OTU (shown for *I ≥* 10). If unstated, bacterial isolates were obtained from humans. See Materials and Methods for precise inclusion criteria of sequences. Neighbor-Net networks were calculated with SplitsTree4 (Huson and Bryant, 2006). Phylogenetic analysis of HB1, HB2 and AB2 was based on 176, 139 and 57 sequences, respectively, using optimal models determined by the AIC criterion (WAG+I+G) with optimal α and Pinv parameters. BAL, bronchoalveolar lavage; STR, sterile body site.

A phylogenetic analysis of the HB1 TerL phage family revealed that it is comprised of three main sublineages: (i) a sublineage consisting primarily of gut metagenomic sequences and gut bacterial isolates (the “GI clade” in **Figure 3A**), (ii) a sublineage consisting nearly exclusively of oral metagenomic sequences and completely devoid of bacterial isolates (the “oral clade” in **Figure 3A**), and (iii) a sublineage consisting primarily of environmental sequences (the “Environmental clade” in **Figure 3A** and **Supporting Text S6**). The phylogenetic distinction between gut and oral sequences was supported with 98% bootstrap support by a maximum likelihood phylogenetic tree after removing potentially recombinant sequences (**Figure S10**). The finding that metagenomic HB1 gut-derived sequences grouped with 16 human-associated bacterial isolates from the gut is consistent with the notion that the human gut is generally dominated by phages exhibiting a lysogenic lifestyle (Reyes et al., 2010; Reyes et al., 2012; Ogilvie and Jones, 2015). In contrast, the oral HB1 clade was devoid of bacterial isolates and grouped with the lytic *Lactococcus lactis* phage 1706, further supporting our prediction that oral phages positive for the HB1 marker should be predominately lytic. To further explore this hypothesis, we filtered oral samples obtained from an orally healthy subject through a 0.2 *μm* pore size filter and performed multiple PCRs on the bacterial and the viral fractions. We were unable to amplify HB1 from any of the PCRs performed on the bacterial fraction, however, we were able to amplify HB1 from the majority of samples corresponding to viral fractions (**Figure S7A**). When the same experiment was performed on the HA marker, the opposite result was obtained: we could amplify HA from all samples originating from the bacterial fraction, yet we could not amplify HA from any of the samples originating from the viral fraction (**Figure S7B**). These experiments support our hypothesis that the HB1 phage family in the oral cavity is likely predominately lytic.

### Spatial distribution of phage family sublineages

Our phylogenetic analysis further revealed TerL sublineages that displayed remarkable specificity to certain oral habitats. For example, the oral clade of the HB1 phage family contained distinct sublineages uniquely associated with the tongue dorsum, and different sublineages that were uniquely associated with supragingival plaque (**Figure S11A**). The HB2 phage family followed a similar oral/gut organization as HB1 (**Figure 3B**), and like HB1 also displayed sublineages uniquely associated with either the tongue dorsum or supragingival plaque. Similar site-specific sublineages were found for the AB2, HA, and PCA1 phage families (**Figure 3C** and **Figures S11B**, **S12A**, respectively). Such exclusive associations between certain TerL phage sublineages and specific oral habitats suggests that proximal habitats within the oral cavity can comprise unique phage communities that remain localized despite constant contact between these habitats mediated by the tongue and saliva. These findings are in line with the site-specialist worldview of the oral cavity microbiome where most microbes in the mouth are found in specific oral habitats (Welch et al., 2019). However, most phage families also contained sublineages obtained from a mixture of oral habitats (denoted as “M” clades, highlighted in green in **Figure 3B** and **Figures S11**, **S12**), possibly an indication that the bacterial hosts of these specific phage family members colonize multiple oral habitats, a hypothesis we further explore below.

### Phage family sublineages potentially associated with pathogenicity

Interestingly, most phage families contained certain clades that were not found in the HMP study. These clades, denoted as “P” clades, are highlighted in yellow in the phylogenetic networks (**Figures 3B, C** and **Figures S11B**, **S12**). The absence of HMP metagenomic sequences from “P” clades was statistically significant (**Table S14**), and confirmed by targeted sequencing in our own cohort of oral samples (see below). This observation can possibly be explained by the fact that the vast majority of human-associated bacterial isolates in “P” clades were either pathogens, were isolated from diseased body sites, were isolated from individuals with a diagnosed disease, or were carriage strains, as indicated in **Table S14**, whereas the subjects participating in the HMP study and in our cohort were healthy (all bacterial isolates belonging to “P” clades are highlighted in **Table S10**). Since “P” clades were absent in healthy individuals, “P” clades could possibly serve as specific biomarkers for detection of potential pathogens in humans. Another intriguing feature of “P” clades was the presence of bacteria isolated from animals (HP2, HA, PCA1), potentially revealing a phage signature of animal-to-human transmission. For example, the “P” clade of HB2 (**Figure 3B**) contains a mixture of human pathogens, carriage strains and sequences isolated from animals, including *Streptococcus suis sv.* JS14 and *Streptococcus porcinus Jelinkova* 176, two human pathogens originally isolated from pigs (**Table S10**).

### Phylogenetic analysis of PCR-amplified sequences supports metagenomic results

To independently confirm phylogenies that were based on HMP metagenomic sequences, we also inferred phylogenies based on PCR-amplified TerL sequences together with HMP metagenomic sequences. In **Supporting Text S9** we show that PCR-amplified alleles obtained from specific oral sites for HB1, HB2, HA, PCA1, PCA2 and AB2 were generally intermixed and indistinguishable from metagenomic alleles obtained from the same body sites. Our analysis also showed that none of the PCR-amplified TerL sequences mapped to “P” clades, further supporting our observation that heathy subjects did not contribute TerL alleles to “P” clades. These results show that our metagenomic-based phylogenetic inferences could be confirmed by targeted sequencing, indicating that the phylogenic patterns we observed in metagenomic datasets were not a result of sequencing or assembly artifacts.

### Temporal stability of phage families

**Figure 4:**
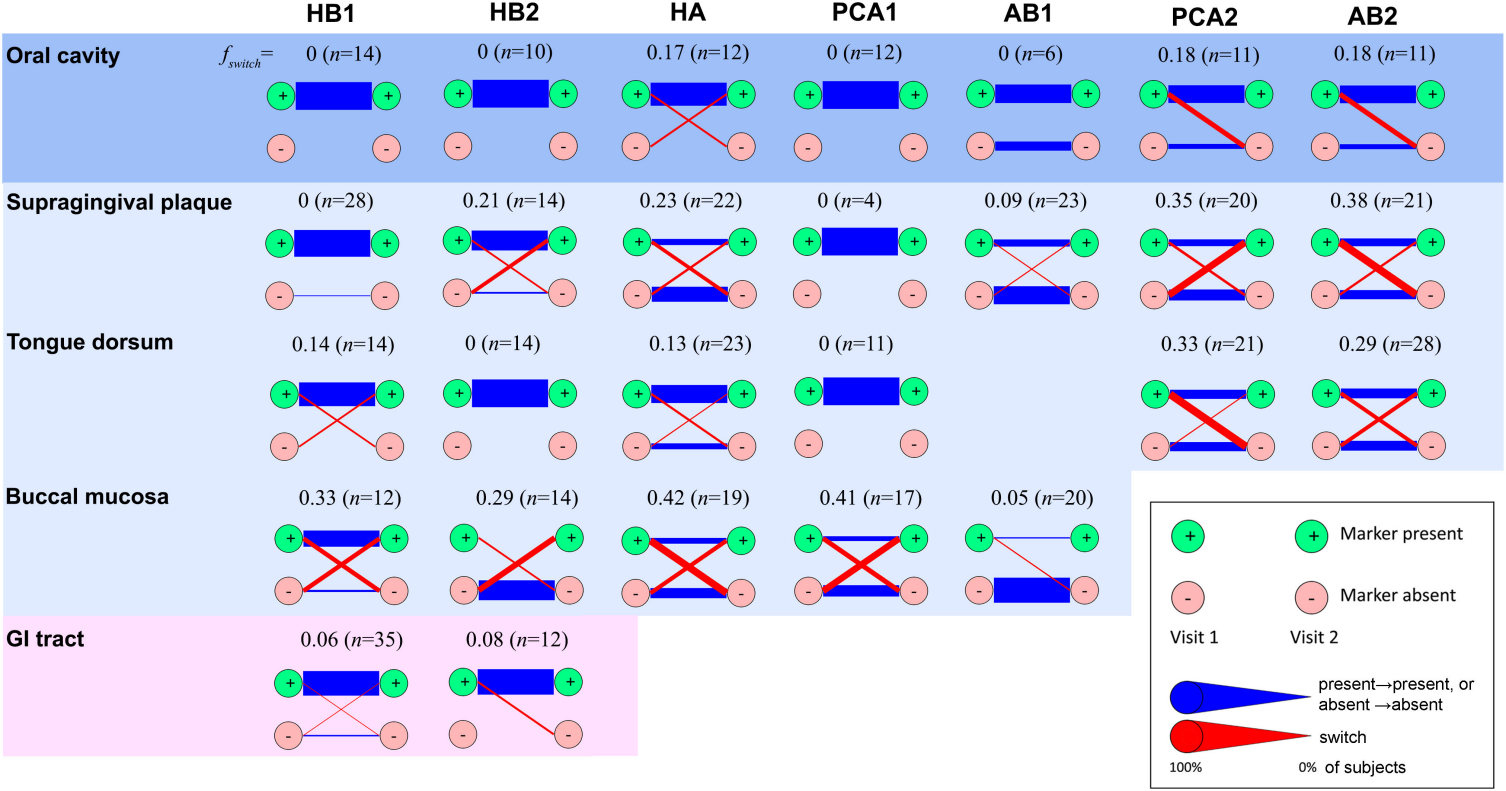
Temporal stability of phage families. Presence of a phage family was determined in two consecutive visits of the same subject considering one metagenome per habitat. In the case of the oral cavity, oral habitats were considered both separately and as a single ecosystem (top row). In the latter case, presence was required in any oral habitat, and absence was required for all oral habitats. A phage family was considered present if any alignment against the corresponding TerL marker sequence spanning at least 150 aa exceeded 70% identity at the amino acid level (see **Supporting Text S4** for optimal alignment length criteria for the HMP metagenomes). Reducing the percent identity threshold to 55% did not have a significant impact on results. To minimize potential coverage bias, a marker was determined to be absent if no alignment spanning a minimum of 75 aa exceeded 40% identity, allowing us to also rule out remote homologs and homologs on short contigs. Blue lines denote unchanged state (presence in both visits or absence in both visits). Red lines denote a change (presence in visit 1 and absence in visit 2, or vice versa). Line widths are proportional to the fraction of subjects that share the same transition. *n* denotes the total number of subjects. Diagrams for habitats for which a marker was found to be always absent were omitted.

Finally, to explore the temporal dynamics of phage families we estimated their persistence across specific body habitats in subjects sampled between two consecutive visits, separated on average by 219 ± 69 (s.d.) days (Huttenhower et al., 2012). We quantified this persistence by measuring the fraction of subjects for which a phage family was detected in the first visit but was absent in the second visit, or vice versa, denoted by *f_switch_* (**Figure 4**). We found that presence of most families (HB1, HB2, PCA1, and AB1) was stable in the oral cavity (*f_switch_* =0), with HB1 and HB2 also stable in the gut (*f_switch_* ≤ 0.08). Indeed, members of a phage family that were present in both visits often had identical amino acid sequences (**Figure S13**), consistent with previous studies that showed that salivary and fecal viromes are genetically stable (Reyes et al., 2010; Pride et al., 2012; Minot et al., 2013; Ogilvie and Jones, 2015; Shkoporov et al., 2019). However, when considering specific oral habitats, most families exhibited considerable temporal variability, with variability highest in the buccal mucosa (*f_switch_* = 0.36 ± 0.06, omitting AB1). One possible explanation for habitat variability could be host migration within the oral cavity. For example, the fact that buccal mucosa-derived sequences typically mapped to “M” clades (clades containing a mixture of sequences from different oral habitats) may indicate that the buccal mucosa contains bacterial hosts that can colonize multiple oral habitats that possibly migrate between different compartments (see examples for potential host migration events in **Figure S13**).

## Conclusions

Much like classical SSU rRNA studies, we found that by focusing our analysis on TerL markers we were able to identify certain TerL phage families that were both conserved and widely shared across the human oral microbiome. This finding is intriguing in light of the tremendous genetic diversity of viruses in nature (Edwards and Rohwer, 2005; Paez-Espino et al., 2016a), the lack of conservation of the TerL gene (Eppler et al., 1991; Chai et al., 1992; Moore and Prevelige, 2002; Rao and Feiss, 2008), and the individualized nature of human viromes established by previous studies (Reyes et al., 2010; Minot et al., 2011; Pride et al., 2012; Reyes et al., 2012; Shkoporov et al., 2019; Moreno-Gallego et al., 2019; Gregory et al., 2020; Zuo et al., 2020; Garmaeva et al., 2021). Overall, the shared TerL lineages we identified accounted for, on average, about 25% of all nonredundant TerL gene families (**Table S15**), adding to the growing body of evidence of the existence of widely shared members of the human virome (Stern et al., 2012; Manrique et al., 2016; Moreno-Gallego et al., 2019).

Although our marker-based approach provided a relatively narrow genomic window into the core human virome, focusing on a single gene enabled us to perform a comparative analysis of this gene across different subjects, different habitats and different time points. Furthermore, our markers, through the use of primers that we developed, enable sequence diversity analysis that is independent of metagenome sequencing. It would therefore be interesting to complement this study with single cell sequencing and genome assembly approaches, which could help shed light on the covariation between different phage families and their bacterial hosts across different body habitats. Furthermore, our analysis focused only on shared phage families within the oral cavity, however, our approach can be extended to other sites to create a comprehensive atlas of shared TerL phage families across the entire human body. More broadly, the fact that we have identified to date phage families with shared TerL lineages in both humans and termites (Tadmor et al., 2011) suggests that phage families with shared TerL lineages across species of organisms may be a common theme in the animal kingdom. Consequently, a comprehensive catalog of ubiquitous TerL phage families could potentially be expanded to encompass other organisms, possibly serving as a useful means for classifying and cataloging recurrent viral diversity core to different organisms.

## Data availability statement

Experimental sequences used in the current study are available at: https://github.com/gitamahm/human_virome.

## Ethics statement

The human samples collected in this study followed Caltech Institutional Review Board IRB protocol 14-0430 and Institutional Biosafety Committee IBC protocol 13-198 with subjects providing written consent. Additional human samples analyzed in this study were provided to us by Bik et al. [The ISME journal 4, 962 (2010)] and were collected in accordance to Stanford IRB protocols.

## Author contributions

AT and RP conceived the study, AT devised and performed the bioinformatic analysis as well as designed the degenerate primers for the markers, GM designed and executed experiments and performed the selection pressure analysis, HF and GM performed the experiments testing bacterial and viral fractions of oral samples, GM and AT performed data analysis related to experiments, and RP scientifically oversaw the project and advised. The paper was written by AT and critically reviewed and edited by all authors. All authors contributed to the article and approved the submitted version.
