# Supplementary material for: Identification and spatio-temporal tracking of ubiquitous phage families in the human microbiome
Source: Front Microbiomes. 2023 Feb 14;1:1097124. doi: 10.3389/frmbi.2022.1097124 (PMC12993540; doi:10.3389/frmbi.2022.1097124)
Supplement: Supplementary file 1 [file DataSheet_1.pdf]

SUPPLEMENTARY MATERIAL

TABLE OF CONTENTS

SUPPORTING TEXT 3

S1. Clustering versus phylogenetic analysis search strategies for shared phage markers 3

S2. Requirements for a viral marker gene 4

S3. Nature of phage-like elements encoding TerL lineages 7

S4. Determining optimal thresholds for BLAST analyses 9

S5. Correlation between marker prevalence in the HMP oral cohort and the hygiene group to which the markers belong 12

S6. Analysis of environmental alleles 13

S7. Investigation of potential biases in metagenomic analyses 19

S8. Diversity of metagenomic sequences in the HMP cohort homologous to the TerL markers 28

S9. Phylogeny of PCR-amplified sequences 33

SUPPORTING FIGURES 38

Figure S1. Overview of methodology and analysis 38

Figure S2. Summary of bioinformatic approach to identify shared TerL markers 39

Figure S3. Conserved functional signatures in TerL lineages 41

Figure S4. Phylogenetic placement of TerL markers. 48

Figure S5. Annotation of the HMP contigs that encode the full-length TerL markers 50

Figure S6. Prophage-like elements harboring close homologs of the TerL markers 51

Figure S7. Amplification of the HB1 and HA markers from bacterial and viral fractions of a saliva sample 53

Figure S9. Diversity of HMP metagenomic sequences homologous to the TerL markers as a function of the applied percent identity threshold 54

Figure S10. Tree-based phylogenetic analysis of the HB1 TerL lineage 55

Figure S11. Phylogenetic analysis of HB1 and HA TerL lineages. 56

Figure S12. Phylogenetic analysis of PCA1 and PCA2 TerL lineages 58

Figure S13. Phylogenetic analysis of TerL lineages recovered from in individual subjects 59

SUPPORTING TABLES ... 61
Table S1. Nonredundant list of viral RefSeq genes reported by MCRL for the metagenomes in the Mira dataset ... 61
Table S2. Nonredundant list of viral RefSeq genes reported by MCRL for the metagenomes in the Mira dataset encoding a TerL gene ... 61
Table S3. Definition of TerL markers and corresponding degenerate primers ... 62
Table S4. Pairwise alignment of full-length TerL markers ... 62
Table S5. Diversity of TerL lineages based on HMP metagenomes and PCR-amplified sequences ... 63
Table S6. Selection pressure analysis of TerL lineages ... 64
Table S7. Conserved functional signatures in TerL lineages ... 66
Table S8. HMP metagenomes that passed HMP quality control criteria ... 66
Table S9. Environmental metagenomes and viromes interrogated for the presence of TerL markers ... 66
Table S10. Bacterial and phage isolates harboring close homologs of the TerL markers ... 67
Table S11. Prevalence of markers in metagenomic studies of stool samples obtained from healthy individuals. ... 68
Table S12. Phylogenetic placement of bacterial and phage isolates harboring close homologs of the TerL markers ... 68
Table S13. Diversity of TerL lineages in individual metagenomes ... 69
Table S14. Statistical evaluation of “P” clades. ... 69
Table S15. Fraction of TerL gene families in the phageome associated with the TerL markers ... 70
Table S16. Statistical analysis of potential biases in the prevalence of markers in HMP and environmental metagenomes ... 71
Supplementary References ... 72

SUPPORTING TEXT

S1. Clustering versus phylogenetic analysis search strategies for shared phage markers

An alternative search strategy to identify shared markers would be to use a combined phylogenetic analysis of all TerL sequences from all databases. Although this method is conceptually more straightforward, in practice it has severe limitations that would lead to poor sensitivity and biased results. There are several reasons for this. First, across biology TerL genes are highly divergent and, as Casjens et al. points out, even when starting with full length TerL genes, encompassing all TerL genes in a single phylogenetic framework is not possible (Casjens et al., 2005). Indeed, in our case, HB1 and AB1 had to be excluded from the combined phylogenetic analysis in Fig. S4 since these sequences were too divergent compared to all other TerL sequences. Thus, a phylogenetic-based search strategy for shared TerL sequences would bias against divergent TerL sequences. This problem is unique to viral sequences given the large sequence space that viral genes typically span. Second, the majority of metagenomic contigs are significantly shorter compared to the length of TerL genes. Therefore, in practice, the vast majority of TerL metagenomic alleles would be too short to be included in a phylogenetic analysis framework, leading to poor sensitivity and further sampling bias (e.g., rarer or less abundant TerL sequences would be negatively biased). For example, in the case of the Mira and Xie metagenomes, the mean contig length was 336 nt and 372 nt, respectively, which is significantly shorter compared to the average length of TerL genes (1650 nt). Thus, a phylogenetic analysis approach to detect shared TerL genes would not be feasible in practice for these metagenomes. Moreover, the combination of short sequences and the highly divergent nature of TerL sequences means that any phylogenetic alignment would target only a small fraction of the TerL gene resulting not just in poor sensitivity (since many sequences would need to be excluded) but potentially also poor specificity. Lastly, a combined phylogenetic analysis approach would require extracting from metagenomes in an unbiased manner contigs encoding TerL genes. However relying on metagenomic annotation for this screening is problematic because contig annotation is often partial or missing, it can vary in quality between datasets and may not be up to date. Our clustering approach using MCRL circumvent these challenges because MCRL has no limit on contig length and is also capable of detecting sequences that are highly divergent from the reference sequences (Tadmor and Phillips, 2022). In addition, MCRL does not rely on metagenomic annotation, and provides for each reported

reference sequence the comprehensive RefSeq annotation for that sequence using the most up-to-date RefSeq release, enabling to systematically screen the annotation records of all reported sequence.

S2. Requirements for a viral marker gene

Analysis of viral genes can be complicated by the fact that certain viral genes can be of bacterial origin and therefore cannot be linked uniquely to viruses, while other viral genes may be simply non-functional degenerating pseudogenes. Therefore, a genuine and biologically relevant viral marker that can be used to track viral diversity across a given species should satisfy certain basic requirements (Casjens, 2003; Tadmor et al., 2011): (1) it should be unique to viruses (whether lytic and or lysogenic), (2) it should be present in a larger genomic viral context such as a prophage or viral genome, (3) it should be prevalent and conserved in the ecosystem being investigated, (4) alleles prevalent in the ecosystem being investigated should contain sufficiently conserved regions that can be used to design degenerate primers (primers are required in order to both validate the marker and apply it experimentally), and (5) the marker should encode a gene that exhibits evidence of functionality in the ecosystem being investigated, ideally proving that the marker is present in viral particles obtained from various samples of the ecosystem being investigated.

The seven TerL markers we identified fulfil of the above requirements in the context of the human microbiome:

(1) The TerL gene is considered to be one of the best phage identifiers because it is found only in phages (both lytic and lysogenic) (Casjens, 2003). The TerL gene is also one of the most universally conserved phage genes because it contains certain functional signatures that are conserved across many phage species, including even certain human eukaryotic viruses (Rao and Feiss, 2008; Sun et al., 2008), suggesting it is an ancient viral domain (Mitchell et al., 2002; Baker et al., 2005; Koonin et al., 2006). In the current study we showed that markers for which we could establish structure-based amino acid alignments (HB1, HB2, HA, PCA1 and PCA2) exhibit intact functional signatures that are universal to TerL genes (see Fig. S3 and Table S7**)**. However, since conserved functional residues span, on average, only ~4% of the length of the TerL gene (outlined in Fig. S3), the majority of residues in the TerL gene are not conserved, and, across biology, TerL proteins of different phages typically exhibit little overall sequence

similarity (Eppler et al., 1991; Chai et al., 1992; Moore and Prevelige Jr, 2002; Rao and Feiss, 2008). The TerL gene therefore appears to be system specific (Black, 1995) and has the potential to serve as an effective differentiating marker between different phage families.

(2) All seven full-length TerL markers were present in HMP contigs that included neighboring phage genes (Fig. S5). Furthermore, six of the seven TerL markers had close homologs in prophage-like elements residing in sequenced bacterial isolates, yielding between 96% to 99% identity at the amino acid level (75% for HB1) across at least 90% of the corresponding marker length (Fig. S6). In all cases, prophage-like elements spanned a minimum of ~20 kb to ~35 kb. BLASTing viral genes responsible for construction of the virion (e.g., terminase genes, portal protein genes, capsid genes, and tail protein genes) from these prophage-like elements against the viral RefSeq database showed that HB1, HA, HB2, and AB2 were strongly associated with the *Siphoviridae* family (6/6 genes, 7/8 genes, 7/7 genes, 4/4 genes, respectively), PCA2 was associated with the *Caudovirales* order (4/6 genes), and PCA1 was associated with unclassified bacterial viruses (5/6 genes). For all the six markers above, viral genes followed a genomic organization pattern that is typical for tailed-phages (Casjens, 2008), with head related genes (e.g., small and large terminase subunit genes, portal protein genes, prohead protease genes, and capsid protein genes) located towards the start of each cassette, followed by tail related genes located towards the end of each cassette. Also in all cases TerL genes were always adjacent to portal protein genes, an exclusive organization in tailed phages (Casjens, 2003).

(3) Our analysis showed that all seven TerL markers were conserved across most amino residues of the TerL gene in a significant percent of subjects in the HMP cohort. For example, when using a 70% identity threshold at the amino acid level and an optimal alignment length threshold of 150 aa (see Materials and Methods), 6 of the 7 TerL markers were found in 68.9% to 97.8% of 90 subjects contributing oral samples (Fig. 2 panel a). Raising the percent identity threshold to 85%, six of the seven TerL markers were found in 43.3% to 88.9% of the 90 HMP subjects contributing oral samples (Fig. 2 panel a). To test these results experimentally, we checked for the presence of the markers in oral samples obtained from three orally healthy subjects using degenerate primers targeting the markers. Our experiments confirmed the existence of PCR-amplified sequences that yielded between 85.7% to 100% identity at the amino acid level when aligned against the markers for six of the seven markers (all markers except AB1) in at least two of the three subjects (Fig. 1 panel a). For three markers (HA, PCA2 and HB1) we further tested our bioinformatic predictions in a cohort of nine orally healthy subjects who provided oral samples from six oral sites (Fig. 1 panel b). We confirmed that for

all three markers we were able to find PCR-amplified sequences that yielded a 100% identity match at the amino acid level to the markers in 9/9 (HA), 7/9 (PCA2) and 6/9 (HB1) of the tested subjects (Fig. 1 panel c). The prevalence of HB1 in our oral cohort was lower compared to its prevalence in the HMP dataset possibly because the HB1 degenerate primer set targeted the C-terminal domain of the TerL gene encoding the nuclease center, which is less conserved compared to the N-terminal domain containing the ATPase center (Rao and Feiss, 2008). In contrast, the HA and PCA2 degenerate primer sets targeted the N-terminal domain encoding the conserved ATPase center and were therefore probably more effective at amplifying TerL alleles (see Fig. S3 for primer positions with respect to TerL domains). Overall, both metagenomic TerL sequences and PCR-amplified TerL sequences exhibited close sequence similarity to the TerL markers at the amino acid level. For example, metagenomic sequences spanning at least 400 amino acids (200 amino acids for AB1) exhibited an average percent identity ranging from 75.9% to 98.1% at the amino acid level when aligned against the corresponding TerL markers, and an average percent identity of 85% to 98.1% when aligned against translated PCR-amplified amplicons (Table S5). Taken together these results indicate that the TerL markers were both highly conserved at the amino acid level and highly prevalent in human subjects.

(4) Amino acid alignments of TerL sequences collected from four independent datasets (HMP, Mira, Xie, HOMD) revealed conserved regions that could be used for primer design. Table S3 shows conserved amino acid motifs targeted by the forward and reverse degenerate primers, and Fig. S3 highlights these conserve motifs in amino acid sequence alignments. Thus, the TerL gene families that we identified were sufficiently conserved to enable design of effective degenerate primers. These degenerate primer sets enabled us in turn to validate our findings in metagenomes using targeted sequencing.

(5) Selection pressure analysis performed on metagenomic sequences from the HMP dataset and from PCR-amplified sequences showed that all TerL lineages were under substantial negative selection with $\omega \ll 1$, where ω represents the ratio of the rate of non-synonymous substitutions to the rate of synonymous substitutions (Table S6). Furthermore, none of the tens to hundreds of TerL alleles we analyzed for each of the markers across ~50 to ~90 HMP subjects (depending on the marker) encoded either errant stop codons or obvious frameshift mutations despite displaying significant sequence variation at the amino acid level (Table S13). Moreover, for five markers for which 3D-stucture based domain models could be fitted

(Marchler-Bauer et al., 2016), terminase-specific functional signatures appeared to be intact across virtually all tested HMP subjects, and for four markers (HA, PCA1, PCA2 and HB2) for which amplicons outside the primer regions overlapped with functional signatures, functional signatures were strictly conserved in 279 of 289 amplicons (96.9%) (Fig. S3 and Table S7). In virtually all cases the observed residues were strictly conserved and represented either the expected motif or residue, a residue found in a corresponding terminase gene with a similar 3D-strucutre from a phage known to be functional, or a substitution that was either biochemically similar or neutral and strictly conserved. In total, we inspected 14,242 residues in alignments of metagenomic sequences of five markers obtained from the HMP dataset, and 862 residues in alignments of alleles of four markers obtained from targeted sequencing, and no obvious violations were observed (data summarized in Table S7).

Finally, close homologs of the markers could be identified in salivary viromes obtained from five subjects, with maximal percent identities in BLAST analyses ranging from 90.4% to 100% identity at the amino acid level for all markers except HB2, and 66.7% identity for HB2 (Fig. 1 panel d). In the case of HB1 we also showed experimentally that only viral fractions of oral samples were positive for the HB1 marker with bacterial fractions being negative for the marker (Fig. S7), suggesting that virus-like particles carrying close homologs of the HB1 marker exist. Taken together, these findings suggest that the TerL lineages are not degenerating pseudogenes experiencing random drift, but are functional genes that are either part of a population of functional phages and/or have been active in recent evolutionary history.

S3. Nature of phage-like elements encoding TerL lineages

Our observations suggest that the TerL lineages corresponding to the markers encode functional TerL genes embedded in phage-like genomic elements. We postulate several scenarios consistent with these observations (Tadmor et al., 2011):

(1) The terminase is part of a functional phage or prophage, as supported by the presence of close homologs of the markers in salivary viromes, and in the case of HB1 also by means of direct experimental detection in viral fractions.

(2) The prophage indeed decayed and the terminase gene degraded over time, but was subsequently repaired by a recombination event with another phage that was likely functional, having had to infect the cell in the first place (Casjens, 2003).

(3) The terminase is part of a defective prophage but the gene remained functional because there was not enough time for point mutations accumulate. This can happen because "prophage-debilitating deletions can accumulate more rapidly than gene-inactivating point mutations" (Casjens, 2003).

Scenarios 2 and 3 would be consistent with the fact that the TerL alleles we observed in metagenomes and using targeted sequencing were under substantial negative selection pressure, contained conserved functional signatures typical of terminase genes, and did not contain obvious frameshift mutations or stop codons.

(4) The terminase was recruited by the bacterium because it confers some competitive advantage to the host and is therefore under negative selection pressure. Examples of phage-like elements recruited by the bacterium that are not genuine phages are bacteriocins and gene transfer agents (GTAs) (Casjens, 2003). Bacteriocins, however, structurally resemble phage tails (Daw and Falkiner, 1996; Casjens, 2003) and do not have heads or package DNA. Therefore, it is unlikely that bacteriocins would encode a terminase gene. For example, type F and type R tail-like bacteriocins of *Pseudomonas aeruginosa* PAO1 do not appear to encode a terminase gene or any other head related proteins (Nakayama et al., 2000; Michel-Briand and Baysse, 2002). GTAs, which are tailed phage-like particles that encapsidate random fragments of the bacterial genome to facilitate horizontal gene transfer (Casjens, 2003), have a typically short coding region spanning ~14-16 kb (Lang and Beatty, 2007) that contains the genes required for assembly of the GTA head and tail structures and the genes required for DNA packaging (including a terminase gene) (Lang and Beatty, 2000; 2007). DNA-specific replication functions and DNA-specific integration or excision functions are not required by the GTA (Lang and Beatty, 2000). Although it cannot be ruled out that the TerL lineages are part of GTAs, this possibility appears to be unlikely given that the predicted prophage-like elements carrying close homologs of the TerL markers are significantly longer than typical GTA lengths (at least ~20 to ~35 kb vs. 14 – 16 kb). Furthermore, none of the TerL markers grouped with the GTA headful clade (Fig. S4). In addition, unlike GTAs, some of the prophage-like elements carrying close homologs of the TerL lineages did, in fact, encode integration

genes (HA, PCA2) and/or DNA replication machinery genes (AB2, HA, HB2). Therefore, it appears unlikely that members of the TerL lineages were adopted by the cell to function as bacteriocins or GTAs.

Although options 2 and 3 are generally plausible, given that close homologs of the TerL markers were also found in viral-like particles suggests that at least those homologs are part of functional phage-like or prophage-like elements. Since in all plausible scenarios (1, 2 and 3) the TerL gene needs to be functional (a small number of random mutations allowed by option 3 should not impact the phylogenetic placement of the sequences), the results presented in our study should provide a biologically relevant picture reflecting present and/or recent phage infections of bacterial hosts and provide an unbiased overview of the ecology of both lytic and lysogenic phages carrying close homologs of these markers.

S4. Determining optimal thresholds for BLAST analyses

Detection of a close homolog of a TerL marker in a metagenome depends on several factors, including: the frequency of the virions or host cells in the sample that harbor close homologs of the marker, the community complexity within the sample, the coverage of the metagenome and the length distribution of contigs in the metagenome. As a general rule, a high alignment length threshold increases the likelihood that a sequence displaying high sequence similarity is genuinely homologous to the query and not a spurious hit. However, as the alignment length threshold increases, depth of coverage becomes a limiting factor, which can increase the rate of misdetection. We therefore wished to determine the optimal alignment length threshold that maximizes the reproducibility of determining presence and absence of close homologs of the TerL markers in metagenomes.

We addressed this tradeoff by objectively determining the optimal alignment length threshold for which the probability of detecting a marker in a given metagenome and not detecting it in a duplicate metagenome from the same sample, or vice versa, denoted $p_{misdetection}$, is minimized. We calculated $p_{misdetection}$ as follows: Let $b_{i,j}^{(m,s)}$ be a binary 2×2 matrix calculated for the m-th marker and the s-th sample denoting four possible outcomes: $b_{+,+}^{(m,s)}$: the m-th marker was detected in both replicates, $b_{-,-}^{(m,s)}$: the m-th marker was not detected in any replicate, $b_{+,-}^{(m,s)}$: the m-th marker was detected in replicated no. 1 but was not detected in

replicate no. 2, and $b_{-,+}^{(m,s)}$: the m-th marker was not detected in replicated no. 1 but was detected in replicate no. 2. The four $b_{\alpha,\beta}^{(m,s)}$ 2×2 matrices were calculated for 15 HMP metagenomes and their replicates (30 metagenomes in total) collected from six body sites (buccal mucosa, supragingival plaque, tongue dorsum, anterior nares, posterior fornix, and retroauricular crease) from eight individuals. A close homolog of a TerL marker was deemed present in a metagenome if the maximum percent identity at the amino acid level exceeding a given alignment length threshold (which is a parameter) was 70% or higher. We chose a threshold of 70% because this threshold captured the primary diversity of close homologs of the markers in the HMP cohort (see Fig. S9 and Supporting Text S8). Averaging the $b_{\alpha,\beta}^{(m,s)}$ matrices across the 15 samples we obtain the following 2×2 probability matrix for the m-th marker:

$$P^{(m)} = \begin{pmatrix} p_{-,-}^{(m)} & p_{-,+}^{(m)} \\ p_{+,-}^{(m)} & p_{+,+}^{(m)} \end{pmatrix},$$

where

$$(P^{(m)})_{\alpha,\beta} \triangleq p_{\alpha,\beta}^{(m)} = \frac{1}{15}\sum_{s=1}^{15} p_{\alpha,\beta}^{(m,s)}$$

The percent of samples for which a marker was detected in one of the replicates and not detected in the other, averaged across all markers, is then given by $p_{misdetection} = \frac{1}{7}\sum_{m=1}^{7}\left(p_{+,-}^{(m)} + p_{-,+}^{(m)}\right)$. Thus, for each alignment length threshold, $p_{misdetection}$ was calculated based on 210 data points (30 metagenomes × 7 markers). Ideally, the $P^{(m)}$ matrices should be diagonal for all TerL markers and $p_{misdetection}$ would be zero. Maximizing BLAST reproducibility then translates to finding the alignment length for threshold for which $p_{misdetection}$ is minimal. The alignment length that yielded the minimal off diagonal elements occurred at a length of 455 $\pm$ 45 nt (s.d.), noted by the arrow in Fig. S4.1 panel a. This optimal length is close to the asymptotic median contig length of a typical HMP metagenome (495 nt) (Methé et al., 2012), and the median contig lengths across these replicates (range $\pm$ 1 s.d.: 419 - 693 nt). The matrices show that BLAST results were reproducible in 14 out of 15 samples (Fig. S4.1 panel b). The percent of samples with inconsistent BLAST results (i.e., misdetection of a marker that is detected in a replicate metagenome) given the optimal alignment length threshold was therefore at most 7%, and 2 $\pm$ 3.4% (s.d.) when averaged across all markers. In the case of environmental metagenomes (Fig. 1 panel g) we used the same alignment length threshold because environmental metagenomes were selected to have a comparable contig

length distribution to that of the HMP metagenomes. For consistency, we used the same alignment length threshold for the IMG/VR metagenomes (Fig. S8). In addition, in order to obtain an upper estimate on marker prevalence in natural environments, in Supporting text S6 we repeated all BLAST analyses with an alignment length threshold of 50 aa.

Applying the same optimality criterion to the Mira, Xie and Pride (oral virome) datasets we set the optimal alignment length thresholds to 120aa for the Mira and Xie datasets, which is close to the mean/median contig lengths in these datasets (336 nt and 409 nt, respectively), and 110 aa for the Pride dataset (Fig. 1 panel d), close to the mean/median contig length in this dataset (328 nt, 348 nt, respectively). In order for our analysis of environmental viromes (Fig. 1 panel h) and oral viromes (Fig. 1 panel d) to be comparable we used the same alignment length threshold (110 aa) for environmental viromes, which is above the minimum read length in this dataset (319 nt), with 94% of environmental viromes having more than 90% of reads longer than 300bp, and 90% of viromes having more than 95% of reads longer than 300bp.

Figure S4.1 Determining the optimal alignment length threshold for BLAST analysis Panel **a** shows $p_{misdetection}$ as a function of the amino acid alignment length threshold (in units of nucleotides). Panel **b** shows the $P^{(m)}$ matrices calculated for the seven TerL markers for the optimal alignment length threshold of 450 nt.

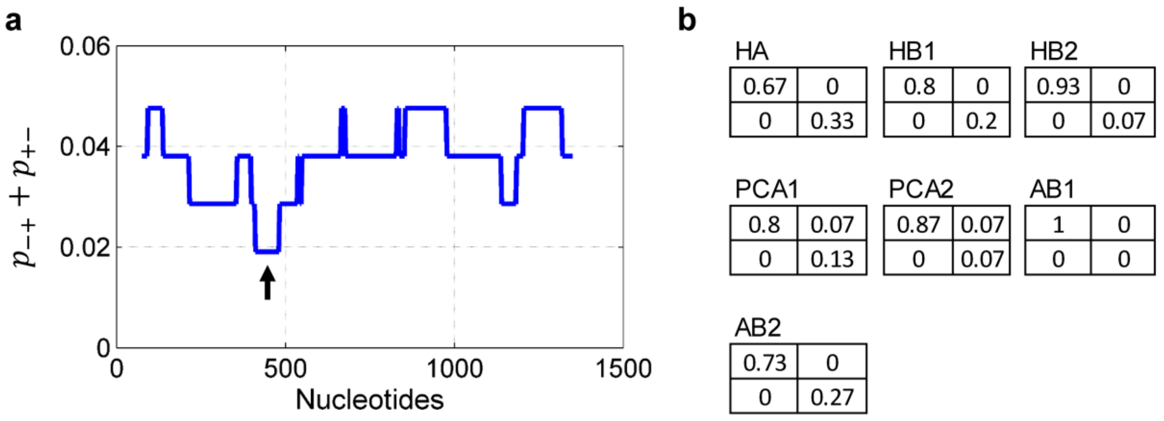

S5. Correlation between marker prevalence in the HMP oral cohort and the hygiene group to which the markers belong

The Mira metagenomes in which the markers were first discovered correspond to subjects with varying degrees of oral hygiene that fall into three categories: good (*H*), mediocre (*PC*) and poor (*A*) oral hygiene (see Materials and Methods). The TerL markers were labeled according to the metagenome in the Mira study in which they were identified (marker naming scheme is explained in Fig. S2): HA, HB1 and HB2 were identified in metagenomes of subjects belonging to the good oral hygiene group (*H*); PCA1 and PCA2 were identified in metagenomes of subjects belonging to the mediocre oral hygiene group (*PC*); AB1 and AB2 were identified in metagenomes of subjects belonging to the poor oral hygiene group (*A*).

Below we show that the better the oral hygiene of the patient from which a given marker was identified, the more prevalent that marker was in the HMP oral cohort. Such a finding is consistent with the fact that subjects included in the HMP cohort all had good oral health (see Materials and Methods for oral health inclusion criteria for the HMP study). Treating the oral hygiene group as an ordinal parameter (good=1, mediocre=2, poor=3), we calculated for the group of 382 oral metagenomes shown in Fig. 1 panel e and Fig. 2 panel a, spanning 90 subjects in total, a Kendall's tau ranging from −0.7 to −0.75 (n=90 subjects, see Fig. 2 panel a) when using a percent identity threshold of 85% at the amino acid level, and −0.6 to −0.7 when using a percent identity threshold of 70%, where we excluded HB1 which had homologous candidates discovered in oral hygiene groups 2 and 3 (Table S2), and where PCA2 - which had a homologous candidate discovered in group 3 (Table S2) - was assigned once to group 2 and once to group 3.

Grouping markers into two categories: no carries (group 1) versus active carries at the time of sampling (groups 2+3), we found a statistically significant difference between the average prevalence of markers in both groups. For example, when using an 85% identity threshold, HA and HB2 were present in 82% of oral samples of the HMP cohort (based on the 90 subjects shown in Fig. 2 panel a), versus an average prevalence of 48% for PCA1, PCA2, AB1 and AB2, yielding a P value of $1.7 \cdot 10^{-6}$ (n=90, two tailed). When applying a 70% identity threshold we obtained a P value of $7 \cdot 10^{-4}$ (84% versus 61%, two tailed). Comparing HA and HB2 (good hygiene group) to AB1 and AB2 (poor hygiene group) using an 85% identity threshold we obtained a P value of $1.2 \cdot 10^{-12}$ (n=90, 81.7% versus 28.9%, two tailed) and when using a 70% identity threshold we obtained a P value of $9.9 \cdot 10^{-8}$ (n=90, 83.4% versus 46%, two tailed).

The same correlation persisted when taking into account one metagenome per subject per oral site, considering only subjects sampled across three specific oral sites (tongue, supragingival plaque and the buccal mucosa). For this group of 159 oral metagenomes spanning 53 subjects, we obtained a Kendall's tau of −0.75 (n=53 subjects) at an 85% identity threshold and a Kendall's tau of −0.64 at a 70% identity threshold. Furthermore, when using an 85% identity threshold HA and HB2 were present in 78.3% of oral samples of the HMP cohort, versus an average prevalence of 33% for AB1 and AB2, yielding a P value of $3.1 \cdot 10^{-6}$ (n=53, two tailed). When applying a 70% identity threshold we obtained a P value of $3 \cdot 10^{-4}$ (n=53, two tailed, 79.2% versus 45.3%).
Restricting our analysis only to subjects for which the total genome size of all three habitats together was above the median genome size in this group, we obtained a Kendall's tau of −0.6 (n=27 subjects) at an 85% identity threshold. At a 70% identity threshold the correlation was weaker (−0.23), although our analysis for this group of metagenomes was less powerful due to the smaller sample size (n=27 versus n=90 in our original analysis) and overall smaller number sequenced metagenomes (382 metagenomes versus 81 metagenomes). Nevertheless, at 85% identity threshold, HA and HB2 were still more prevalent in a statistically significant manner compared to AB1 and AB2 despite the small sample size (P value = 0.0095, n=27, two tailed, 74% versus 39%).

Taken together, these results suggest that across large datasets, close homologs of markers derived from orally unhealthy subjected were statistically less prevalent in a population of orally healthy individuals compared to markers derived from orally healthy subjects. This difference may reflect, for example, a difference in the oral community composition of orally healthy versus orally unhealthy subjects, or, given the weaker results in metagenomes with a high genome size, may reflect a difference in the abundance of phages (or hosts) associated with markers belonging to good versus mediocre or poor oral hygiene groups. Such a conclusion would generally be consistent with the observed correlation between certain oral microbial compositions and periodontal health (Chen et al., 2018a).

S6. Analysis of environmental alleles

We searched for homologs of the markers across three environmental metagenomic databases: 233 environmental metagenomes from the IMG/M database (Chen et al., 2018b), 3663

environmental metagenomes from the IMG/VR database comprising nearly 20 million viral contigs (Paez-Espino et al., 2016) and 109 environmental DNA viromes from the VIROME database (Wommack et al., 2012). The table below compares the prevalence of the markers across these three datasets with the prevalence of the markers in the oral cavity of 53 subjects. Prevalence was calculated using two percent identity thresholds: 70% and 55% at the amino acid level, and using two alignment length thresholds: 150 aa (the threshold used for whole community metagenomes in Fig. 1) and 50 aa. Although at a threshold of 50 aa the specificity of the search is lower, in particular at the lower percent identity range, results in this range can provide an upper estimate on the prevalence of markers in the environment.

The table shows that at 70% identity, HB1 was present in about 6% to 11% of environments, compared to 96% to 100% prevalence in the oral cavity. The other markers were generally absent from the environment. The table further shows that at 70% identity, marker prevalence was similar across the three environmental datasets, with the exception of HB1 which was negative in environmental viromes. These results were not dependent on the alignment length threshold. This table therefore shows that our smaller panel of 233 environmental metagenomes were a representative sample set consistent with results based on environmental viromes. At 55% identity, marker prevalence in the environment generally remained in the same range, except for HB2, which showed an elevated prevalence. Close homologs of HB1 and more distant homologs of HB2 in natural environments were phylogenetically distinct compared to close homologs of these markers in human and animal microbiomes (see below).

Prevalence of HB1 and HB2 in natural environments

HB1

Close homologs of the HB1 marker could be found in a small subset of environmental metagenomes. Although negative in environmental viromes, HB1 was positive in 6% to 7% of environmental metagenomes based on the IMG/M and IMG/VR databases (Table S6.1) and positive in 13.2% of environmental metagenomes with above median genome size (Supporting Table S5). However, Fig. 3 panel a shows that environmental bacterial and phage isolates harboring close homologs of HB1 did not group with the two main clades of the HB1 lineage corresponding to the oral cavity and the GI tract, and instead formed an independent clade, which we denoted as the ‘environmental clade’ in Fig. 3 panel a. When including HB1 alleles from environmental metagenomes (Fig. 1 panel g) in our phylogenetic analysis shown in Fig.

3 panel a we find that environmental metagenomic alleles indeed grouped within the environmental clade (Fig. S6.1).

Consequently, based on our search for homologs of the HB1 marker in the HMP cohort comprising of nearly 700 metagenomes, all publicly available sequenced bacterial and phage isolates, and hundreds of environmental metagenomes and viromes, close homologs of HB1 appear to fall into three main sublineages: an oral sublineage, a gut sublineage, and an environmental sublineage enriched with environmental alleles. Therefore, while HB1 is to a certain extent prevalent in the natural environments, HB1 alleles found in humans and animals appear to be generally specific to humans and animals.

HB2

Although environmental metagenomes and environmental viromes generally did not contain close homologs of HB2 exceeding 70% identity at the amino acid level (only 1 of 233 environmental metagenomes in IMG/M yielded a hit exceeding 70% identity, 3 of 3663 environmental metagenomes in IMG/VR yielded a hit exceeding 70% and no close hits were detected in environmental viromes), more distant homologs yielding alignments in the range of 50% to 65% identity at the amino acid level were more prevalent: 6.4% of environmental metagenomes in Fig. 1 panel g yielded >50% identity (median 60%), and 27.5% of environmental viromes in Fig. 1 panel h yielded >50% identity (median 53%). Fig. S6.2 shows the phylogeny of human and animal associated HB2 alleles in conjunction with environmental alleles from metagenomes and viromes. Fig. S6.2 shows that alleles obtained from environmental metagenomes and environmental viromes were significantly divergent with respect to human and animal associated alleles, suggesting that the diversity of HB2 alleles found in the environment is distinct from the diversity of HB2 alleles found in humans and animals.

Table S6.1 Prevalence of TerL markers across HMP oral metagenomes versus environmental datasets. Environmental datasets correspond to the data shown in Fig. 1 panel g (*n*=233 environmental metagenomes from the IMG//M database), Fig. S8 (viral contigs from *n*=3663 environmental metagenomes in the IMG/VR database), Fig. 1 panel h (*n*=109 environmental DNA viromes from the VIROME database). For the HMP oral cohort, for each of the *n*=53 subjects analyzed, the maximum percent identity was selected across three oral habitats (buccal mucosa, supragingival plaque and the tongue dorsum) sampled in the same visit. Alignment length thresholds are indicated at the top. Percent identities are calculated at the amino acid level. HB1 and HB2, which had homologs in the environment, are highlighted in the table.

	150 aa				50 aa			
	Oral cavity (*n*=53 subjects)	Environmental metagenomes (*n*=233)	IMG/VR (*n*=3663 metagenomes)	Environmental viromes (*n*=109)	Oral cavity (*n*=53 subjects)	Environmental metagenomes (*n*=233)	IMG/VR (*n*=3663 metagenomes)	Environmental viromes (*n*=109)
70% identity threshold								
**HB1**	96.2	6.0	6.9	0	100.0	10.7	7.0	1
**HB2**	71.7	0.4	0.1	0	94.3	1.3	0.1	0
**PCA2**	66.0	0	0	0	73.6	0	0	0
**PCA1**	86.8	0.4	0	1	88.7	0.4	0	1
**HA**	86.8	0	0	0	90.6	0	0	0
**AB2**	69.8	0	0	0	75.5	0	0	0
**AB1**	20.8	0	0	0	22.6	0	0	0
55% identity threshold								
**HB1**	98.1	6.0	7.1	0	100.0	11.6	7.2	1
**HB2**	90.6	3.9	1.0	0	100.0	11.6	1.1	24.8
**PCA2**	73.6	0	0	0	79.2	0	0	0
**PCA1**	86.8	0.4	0.1	0.9	96.2	1.3	0.1	2.8
**HA**	86.8	0	0	0	90.6	0	0	0
**AB2**	69.8	0.9	0	0	75.5	1.3	0	2
**AB1**	39.6	1.3	0	0	47.2	2.1	0	1

Figure S6.1 Phylogenetic analysis of close homologs of the HB1 marker obtained from the HMP cohort and environmental metagenomes. Neighbor-Net analysis drawn with SplitsTree4 (Huson and Bryant, 2006) for 366 unambiguous amino acid residues of the HB1 TerL marker. The Neighbor-Net analysis included all alleles from Fig. 3 panel a and all close homologs of HB1 found in the 233 environmental metagenomes analyzed in Fig. 1 panel g, following the same inclusion criteria for alleles as in Fig. 3 panel a. Phylogenetic analysis was based on 189 sequences using the optimal WAG+I+G model based on the AIC criterion with optimal α and Pinv parameters.

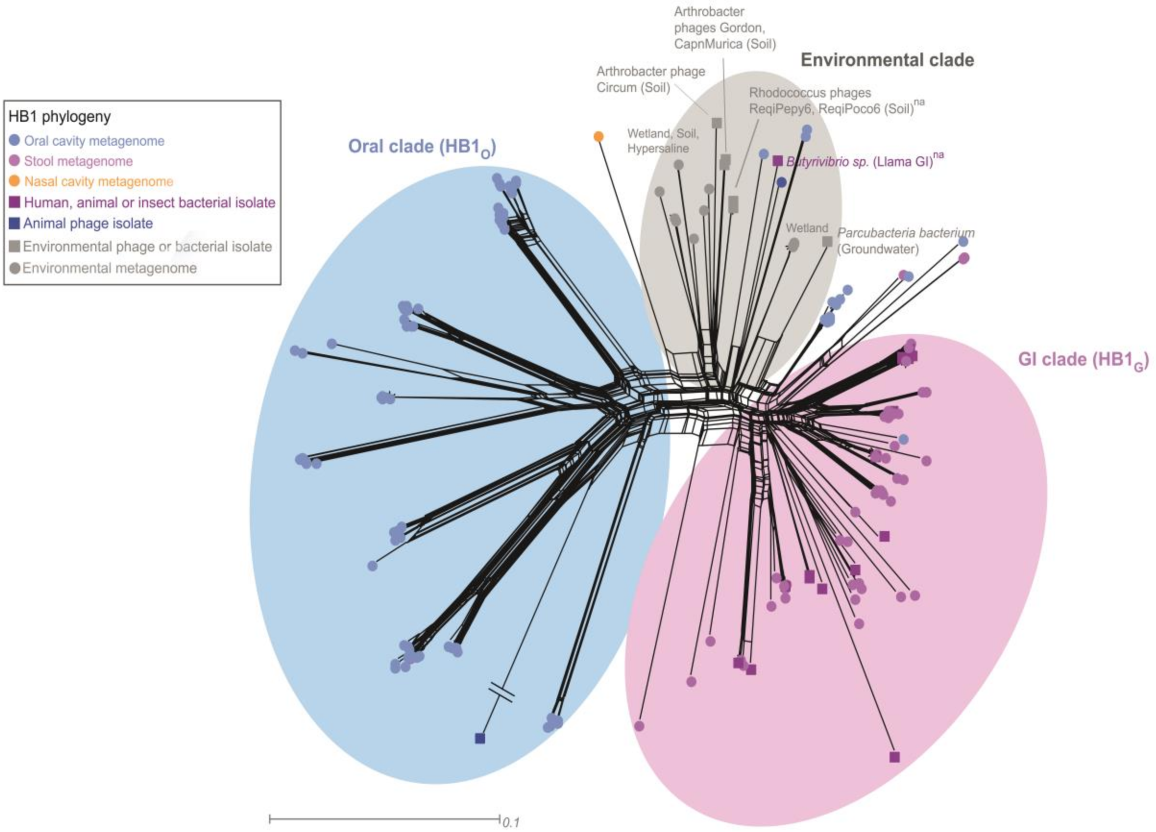

Figure S6.2 Phylogenetic analysis of close homologs of the HB2 marker obtained from the HMP cohort, environmental metagenomes and environmental viromes. Neighbor-Net analysis drawn with SplitsTree4 (Huson and Bryant, 2006) for 165 unambiguous amino acid residues of the HB2 TerL marker. The Neighbor-Net analysis included all alleles from Fig. 3 panel b, and, in addition, all distant homologs of HB2 found in the 233 environmental metagenomes analyzed in Fig. 1 panel g and the 109 environmental viromes analyzed in Fig. 1 panel h that yielded at least 50% identity and spanned at least 180 amino acids when aligned against the HB2 marker. Phylogenetic analysis was based on 166 sequences using the optimal WAG+I+G model based on the AIC criterion with optimal α and Pinv parameters.

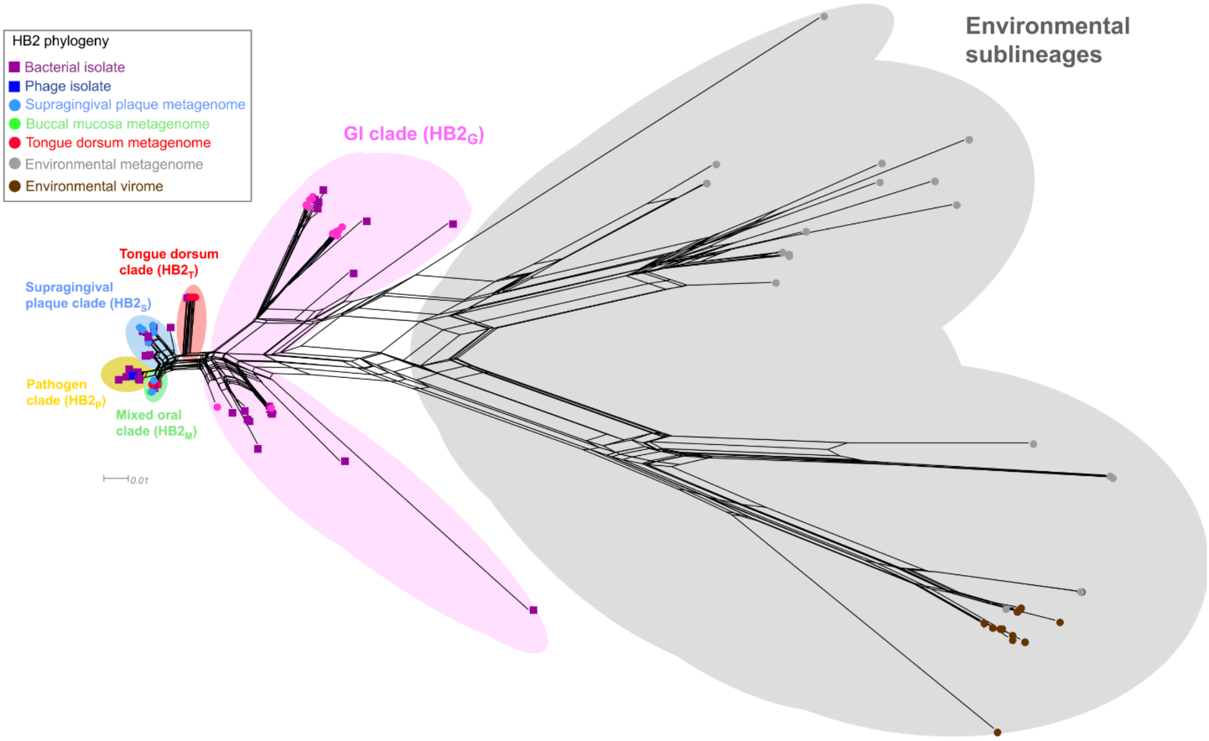

S7. Investigation of potential biases in metagenomic analyses

One of the challenges of searching for markers in metagenomic datasets is that factors related to coverage, community complexity and assembly can bias search results and lead to misdetection of alleles. For example, presence of markers can potentially be obscured in metagenomes with short contig lengths and/or low coverage, in particular for ecosystems with high complexity community structures, and lead to an underestimation of the prevalence of certain markers in certain body habitats or environments. We therefore explored potential sources of bias in our analysis of both HMP and environmental metagenomes.

Potential biases in analysis of HMP metagenomes

Genome size

In terms of community complexity, the genome size of HMP metagenomes was generally correlated with the alpha diversity of different body habitats (38)($\rho = 0.73$, Fig. S7.1 panel a) suggesting that sequencing depth in HMP metagenomes was generally sufficiently high to capture the diversity of microbial communities in these environments. Since diversity and genome size were correlated in the HMP cohort, we explored whether the genome size parameter (total number of assembled coding contigs) had an impact on the prevalence of markers in the metagenomes across different body habitats. To check this, we divided the metagenomes in each body habitat analyzed in Fig. 1 panel f and Fig. 2 panel c into two groups: metagenomes with a genome size above and below the median genome size of metagenomes in the corresponding body habitat group (median genome sizes for each body habitat are shown in Table S18 panel a). We then tested whether the difference in marker prevalence between both groups was statistically significant using a 70% identity threshold at the amino acid level and applying a two tailed Z test (Table S16 panel a). We found that for most markers and body habitats there was no statistically significant difference between both groups of metagenomes. However, for a small subset of marker/habitat combinations genome size was limiting (highlighted in yellow in Table S16 panel a). For example, the prevalence of PCA1 in buccal mucosa increased from 40.6% (*n*=64 subjects) to 53.1% when considering only metagenomes above the median genome size in this body habitat. Similarly, the prevalence of PCA2 in supragingival plaque increased from 31% (*n*=71 subjects) to 55.6%, and in the tongue dorsum prevalence increased from 47.9% (*n*=71 subjects) to 66.7%, respectively. Finally, prevalence of HB1 in stool increased from 78% (*n*=82 subjects) to 92.7%. Repeating our statistical analysis for the subset of metagenomes in each body habitat with a genome size above the median (Table S16 panel b), only three marker/habitat combinations were still limiting: the prevalence

of PCA2 in supragingival plaque and the tongue dorsum increased from 55.6% to 72.2%, and from 66.7% to 83.3%, respectively, and the prevalence of HB2 in stool increased from 48.8% to 61.9%. Therefore, the prevalence of markers across different body habitats shown in Fig. 2 was slightly underestimated for certain combinations of markers and body habitats in the oral cavity and stool, in particular for HB2 and PCA2.

Contig length

To check whether our BLAST-based search for close homologs of the markers in HMP metagenomes was biased by the mean contig length, we separated metagenomes in different body habitats again into two groups: metagenomes with a mean contig length above and below the median, and we repeated our statistical analysis (Table S16 panel c). We found that except for HA in the buccal mucosa, there was no statistically significant difference in the prevalence of TerL markers between HMP metagenomes with contigs lengths below and above the median contig length, suggesting that overall contig length was not a limiting factor in our analysis.

Figure S7.1 Alpha diversity in HMP metagenomes and distribution of contig length and genome size in HMP and IMG/M and environmental metagenomes. a, Median genome size is plotted as a function relative alpha diversity based on 16S phylotypes (38) for HMP metagenomes across seven main body habitats. PF=posterior fornix (n=30 subjects), RC=retroauricular crease (n=7 subjects), BM=buccal mucosa (n=64 subjects), AN=anterior nares (n=54 subjects), S=stool (n=82 subjects), TD=tongue dorsum (n=71 subjects), SP=supragingival plaque (n=71 subjects).Vertical bars correspond to 25% and 75% percentiles. ρ denotes Pearson's linear correlation coefficient. **b**, Distribution of average contig length of oral HMP metagenomes passing HMP quality control criteria analyzed in Fig. 1 and Fig. 2 (blue), and IMG/M environmental metagenomes analyzed in Fig. 1 panel g (red). **c,** Cumulative length distribution of genome size of HMP oral metagenomes passing HMP quality control criteria (red) and IMG/M environmental metagenomes (blue).

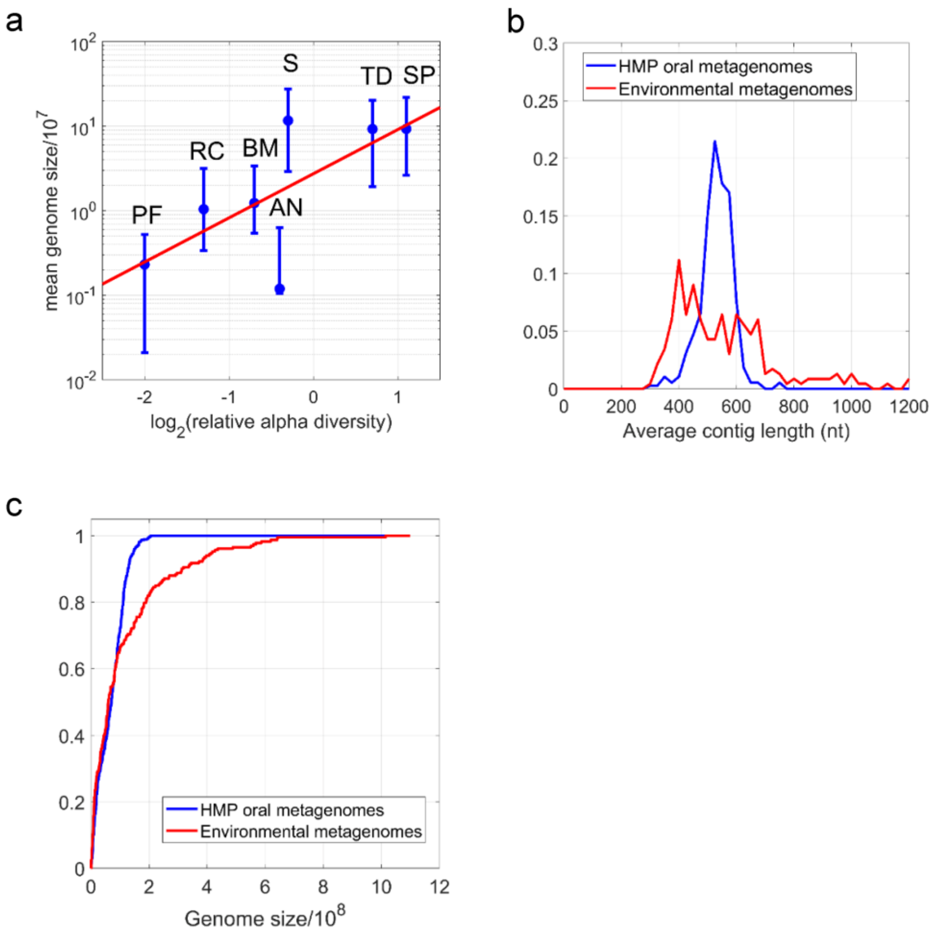

Potential biases in analysis of IMG/M environmental metagenomes

Any negative bias in our search for homologs of the TerL markers in environmental metagenomes can lead to an underestimation of the prevalence markers in the natural environments. In this section we describe the measures we took to minimize such biases (e.g., due to genome size and contig length), and assess whether our search for homologs of TerL markers in IMG/M environmental metagenomes was biased in a statistically significant manner by any experimental or bioinformatic factor, such as contig length, genome size, community complexity, read depth, method of assembly, or sequencing technology. We further confirm that our statistical analysis is robust to changes in the percent identity threshold. In addition, we assessed potential sampling biases and biases related to database selection.

Minimizing potential biases due to contig length and genome size in analysis of IMG/M environmental metagenomes

To minimize potential biases related to contig length and genome size and enable us to compare results from environmental metagenomes (Fig. 1 panel g) and oral metagenomes (Fig. 1 panel f) using a single set of thresholds, we controlled for both the contig length and the genome size when selecting IMG/M environmental metagenomes such that the distribution of contig lengths and genome sizes of the environmental metagenomes would be comparable to that of oral HMP metagenomes. For the contig length parameter, this was done by excluding environmental metagenomes whose average contig length was below the minimum contig length of HMP oral metagenomes analyzed in Fig. 1 panel f, which was 300 bp (the assembly parameter threshold for scaffolds used in the HMP study (7)). The resulting distribution of contig lengths of environmental metagenomes analyzed in Fig. 1 panel g was comparable to that of the oral metagenomes analyzed in Fig. 1 panel f (see Fig. S7.1 panel b), with both distributions yielding similar means (563 nt for environmental metagenomes compared to 529 nt for the oral metagenomes), allowing us to use a single alignment length threshold for both datasets.

To control for the genome size of metagenomes, we excluded IMG/M environmental metagenomes with a genome size that was lower than the minimal genome size of HMP oral metagenomes analyzed in Fig. 1 panel f. The resulting distribution of genome sizes in the final set of environmental metagenomes included in Fig. 1 panel g had an overall higher genome size compared to the oral metagenomes included in Fig. 1 panel f, with a mean genome size of $1.2 \cdot 10^8$ nt compared to $0.7 \cdot 10^8$ nt for the oral metagenomes (Fig. S7.1 panel c).

Potential biases related to genome size

To check whether our BLAST-based search for close homologs of the markers in the final set of 233 IMG/M environmental metagenomes included in Fig. 1 panel g was biased by genome size, we investigated whether the prevalence of close homologs of each marker across the final set of environmental metagenomes was dependent on the genome size. Following the same statistical framework described above for the HMP metagenomes, we divided the 233 environmental metagenomes into two groups: metagenomes with a genome size above the median genome size ($5.9 \cdot 10^7$ nt), and metagenomes below the median genome size, and compared the prevalence of close homologs between both groups. We found that except for HB1, there was no statistically significant difference between the two groups (Table S16 panel d, HB1 highlighted in yellow). To check whether we underestimated the distribution of close homologs of the HB1 marker in the environment, we retained only environmental metagenomes with a genome size that was above the median genome size of HMP oral metagenomes shown in Fig. 1 panel f ($7 \cdot 10^7$ nt), remaining with 106 environmental metagenomes (the possible impact of community complexity will be discussed below). In the group of environmental metagenomes with high genome size, the percent of metagenomes carrying a close homolog of HB1 indeed increased from 6% (Table S16 panel d) to 13.2% (Table S16 panel e). However, repeating our statistical evaluation in the group of environmental metagenomes with a high genome size, we found no statistically significant difference between the group of environmental metagenomes below and above the median genome size in this group of metagenomes (Table S16 panel e), suggesting that HB1 can potentially be prevalent in up to 13.2% of environments. A prevalence of 13.2% is still significantly lower compared to the prevalence of close homologs of HB1 in oral sites such as supragingival plaque (89.8% across 71 subjects, Table S16 panel e). Furthermore, a low incidence rate in environmental metagenomes is consistent with our finding that close homologs of the HB1 marker were found is a small number of hosts isolated from natural environments, including: *Rhodococcus equi,* a soil dwelling pathogen of animals and humans, *Arthrobacter sp.*, another soil dwelling bacterium, and *Parcubacteria* bacterium that was isolated from ground water (Table S10). Environmental alleles related to HB1, belonged, however, to a distinct phylogenetic lineage compared to human-associated alleles (see Supporting text s6 for a phylogenetic analysis).

To check whether the percent identity threshold had an impact on results, we repeated our statistical analysis applying a 55% identity threshold at the amino acid level, which is the lower threshold for encompassing remote homologs comprising distant lineages in the human microbiome (Supporting Text S7). Reducing the percent identity threshold to 55% did not change the results, with the exception of HB2, which at this percent identity threshold was limited by genome size (Table S16 panel h). Repeating this statistical analysis for environmental metagenomes with high genome size we found that HB2 was borderline significant, with the percent of environmental metagenomes positive for HB2 at this lower percent identity threshold increasing from 3.9% to 11.3% when considering only metagenomes above the median genome size (Table S16 panel i), which is closer the prevalence of HB2 observed in environmental viromes (Fig. 1 panel h). Environmental alleles related to HB2, however, as in the case of HB1, belonged to a distinct phylogenetic lineage compared to human-associated alleles (see Supporting text s6 for a phylogenetic analysis).

Potential biases related to contig length

To check if the average contig length was a limiting parameter in our analysis, we repeated the same statistical analysis for the average contig length parameter, comparing the prevalence of close homologs of the markers in metagenomes with an average contig length above and below the median contig length of environmental metagenomes (521 nt). Table S16 panel d shows that apart for HB1 there was no statistically significant difference between the two groups, and in the case of HB1 there is no evidence that higher contig length increases prevalence. Reducing the percent identity threshold to 55% identity at the amino acid level did not change these results (Table S16 panel h). In Supporting text s6 we further show that the prevalence of close homologs of the markers in environmental datasets (IMG/M, IMG/VR and VIROME) was not dependent on the alignment length threshold, which indirectly reflects the contig length distribution.

Potential biases related to coverage

To estimate the average coverage of different populations in environmental metagenomes we calculated the median read depth per base for five representative ribosomal proteins (pfam00318, pfam00347, pfam00411, pfam00573, pfam01196), and averaged these results. We then repeated the same statistical analysis described above for the read depth parameter, comparing the prevalence of close homologs of the markers in environmental metagenomes with an estimated read depth above and below the median read depth (17.2 reads per base). We

found that the read depth parameter did not impact the prevalence of markers in environmental metagenomes (Table 17 panel d). Repeating this analysis using a 55% identity threshold at the amino acid level also did not reveal statistically significant biases. Taking the mean instead of the median read depth did not impact results (Table S16 panel h).

Potential biases related to community complexity

Another confounding factor that can potentially bias our search for homologs of the markers in environmental metagenomes is the community structure in the underlying sample. Samples with complex community structures need to sequenced at a higher depth of coverage to uncover the full extent of the underlying diversity. We therefore checked for potential biases related to the degree of community complexity alone and in conjunction with the coverage of the environmental metagenomes.

Estimating community complexity using ribosomal RNA phylotypes

Since the degree of community complexity should generally be correlated with the number of ribosomal RNA phylotypes detected in a metagenome, we divided environmental metagenomes again into two groups: metagenomes in which the number of ribosomal RNA phylotypes detected in the sample was either above or below the median number of RNA phylotypes detected in the given metagenome. The number of ribosomal RNA phylotypes in environmental samples was determined by the standard DOE-JGI Metagenome Annotation Pipeline (MAP) (Huntemann et al., 2015) using INFERNAL 1.1.2 ("INFERence of RNA ALignment") (Nawrocki and Eddy, 2013). INFERNAL searches a sequence database (Rfam) for homologs of structural RNA sequences to identify structural RNAs in both sequenced genomes and metagenomes hosted on IMG/M. INFERNAL uses a covariance model to score both sequence consensus and RNA secondary structure consensus, thereby enabling it to detect also distant homologs (Nawrocki and Eddy, 2013). To increase the robustness of our statistical analysis, we performed our analysis for each of the three ribosomal RNA genes (5S rRNA, 16S rRNA and 23S rRNA) separately. Table S16 panel d shows that detection of close homologs of the TerL markers was not negatively biased in the group of metagenomes with a high number of rRNA phylotypes. Reducing the percent identity threshold to 55% at the amino acid level did not change these results (Table S16 panel h).

Estimating community complexity using ribosomal proteins

To confirm our statistical analysis of community complexity based on rRNA markers, we also estimated community complexity based on counts of ribosomal proteins, focusing on the five ribosomal protein families described above (pfam00318, pfam00347, pfam00411, pfam00573, pfam01196). Consistent with our findings based on the rRNA markers, detection of close homologs of the TerL markers was not negatively biased in the group of metagenomes with a high number of ribosomal proteins (Table S16 panel d). Reducing the percent identity threshold to 55% at the amino acid level did not change these results (Table S16 panel h).

Potential biases related to community complexity and read depth

To check whether community complexity in conjunction with coverage was a limiting factor in our analysis, we repeated our analysis separately for high coverage environmental metagenomes (>17.2 reads per base, Table S16 panel f) versus low coverage environmental metagenomes (<17.2 reads per base, Table S16 panel g). We found that when restricting our analysis to metagenomes with a high or low read depth there was no statistical difference between metagenomes with low versus high number of ribosomal protein counts. Repeating this analysis for rRNA phylotypes showed there was no evidence that markers were negatively biased in high complexity low coverage environments. Reducing the percent identity threshold to 55% at the amino acid level did not change these results, and only reinforced that prevalence of distant homologs of HB1 and HB2 is elevated in high complexity low coverage environments (Table S16 panels j and k), which is consistent with the fact that these markers were also elevated in environmental metagenomes with high genome size.

Potential biases related to assemblers or sequencing technology

We further checked whether grouping environmental metagenomes based on the assembler (e.g., PGA versus Newbler versus other assemblers) or grouping environmental metagenomes based sequencing technology (454 versus Illumina versus Sanger) had an on impact on the prevalence of TerL markers, including remote homologs yielding >55% identity at the amino acid level. We found no statistically significant result at the 0.05 significance level except for HB1 and HB2, which were enriched in metagenomes sequenced with Illumina sequencers, however this was likely because sediment environments, which were enriched for close homologs of HB1 and HB2, were primarily sequenced using Illumina sequencers (~50% of HB1 and HB2 hits were found in sediment environments, and ~80% of sediment environments were sequenced using Illumina sequencers).

Summary of statistical analyses

To conclude, apart from HB1 and to a lesser extent HB2, whose prevalence was elevated in high complexity environments (Table S16 panels d and h), our statistical analyses did not reveal negative biases in our search results related to factors such as contig length, genome size, community complexity, coverage (including low coverage/high complexity environments), assembly method or sequencing technology. We further showed that our results were robust to the applied percent identity threshold.

Sampling or database bias

To independently confirm our findings in environmental metagenomes and rule out potential methodological sampling or database biases, we also searched for homologs of the markers in: (i) nearly 20 million viral contigs from the IMG/VR database obtained from 3663 environmental metagenomes, (ii) nearly 10 million protein sequences included in NCBI's non-redundant environmental database env_nr, (iii) 109 viromes of DNA viruses obtained from natural environments (Fig. 1 panel h), and (iv) we performed an exhaustive search for close homologs of the markers across all sequenced bacterial and phage isolates in major public databases (IMG and NCBI's nr protein database). When considering together our search results in all datasets a consistent picture emerges: close homologs of the TerL markers, with the exception of HB1 and HB2, are generally specific to human and animal microbiomes (see also main text and Supporting text s6). Close homologs of HB1 were detected in a small subset of natural environments, and relatively distant homologs of HB2 were detected in a larger subset of natural environments. However, phylogenetic analysis of environmental alleles of HB1 and HB2 shows that these alleles form separate phylogenetic sublineages that are generally distinct from those of humans/animals (Supporting text s6).

In our search for the presence of marker homologs in natural environments we investigated three types of sample sets: whole community metagenomes, viromes and sequenced isolates. Given that these three samples sets are qualitatively different with different biases associated with them, a systematic bias that would apply to all three sample sets that would account for the lack of homologs in natural environments appears to be unlikely.

S8. Diversity of metagenomic sequences in the HMP cohort homologous to the TerL markers

To determine a percent identity threshold at the amino acid level that captures the diversity of closely related homologs of the TerL markers and defines TerL lineages we explored the diversity of TerL sequences in the HMP metagenomes displaying homology to TerL markers as a function of the applied amino acid percent identity threshold. Fig. S9 shows that in the case of HA and AB2 a 70% identity threshold at the amino acid level captured all TerL sequence diversity apart from a small percent of distant homologs yielding less than 45% identity. For the other markers, the stepwise nature of the resulting plots in Fig. S9 suggests that additional more distant lineages exist, however, the majority of sequence diversity of alleles closely related to these markers, forming the 'primary' lineage for each of these markers, was captured when applying a 70% identity threshold. We therefore empirically defined a TerL lineage - or a TerL 'primary' lineage - associated with a marker to be the collection of alleles displaying 70% identity or more at the amino acid level when aligned against the given marker, and we refer to phylogenetic diversity within a lineage as sublineages. In the case of HB1 and PCA2, diversity appeared to extend somewhat below 70%, however including these more divergent alleles in a network analysis together with the 'primary' lineages of these markers resulted in only further diversifying existing sublineages without adding qualitative new features to the network (see below).

Diversity beyond the 'primary' lineages

Fig. S9 suggests that the TerL (primary) lineages analyzed in this study are part of larger ensembles of diversity. The more divergent alleles associated with the TerL markers were not related to lineages of other markers: when collecting for each TerL marker over 400 alleles from the 690 HMP metagenomes using a 40% identity threshold, no allele associated with marker i was found in the ensemble of alleles collected for marker $j \neq i$. Furthermore, the divergent groups of alleles corresponding to stepwise increases in diversity shown in Fig. S9 are, in fact, cohesive ensembles of closely related alleles that can be thought of as 'secondary' lineages or divergent sublineages of the markers. Table S8.1 compares the within-group average percent identity of secondary lineages corresponding to a given "step" in Fig. S9 (noted by arrows in Fig. S9) with the average percent identity of these alleles with respect to the given TerL marker. Table S8.1 shows that secondary lineages have a much higher within-group similarity compared to their overall percent identity with respect to the markers. For example, in the case of AB2, the group of six alleles yielding 44.4±0.8% identity at the amino acid level

when aligned against the AB2 marker have a within group percent identity of 78.8%, suggesting that these alleles are part of cohesive ensemble of closely related alleles forming a distantly related sublineage for the AB2 marker. Indeed, all divergent alleles in this case were obtained from the anterior nares and the retroauricular crease, body sites that were not associated with the ‘primary’ lineage. Similarly, in the case of PCA2, the group of 69 alleles yielding 45.6±1.8% identity at the amino acid level when aligned against the PCA2 marker had a within group percent identity of 71.4%. Here to, nearly all divergent alleles were obtained from stool, a body site that was not associated with the ‘primary’ lineage of this marker. In another example, the group of 245 alleles yielding 64.1±1.3% identity at the amino acid level when aligned against the HB2 marker manifested phylogenetically as novel divergent sublineages of the ‘primary’ lineage, as shown below.

Table S8.1 Diversity of distant homologs of the TerL markers. “% identity compared to marker”: average percent identity at the amino acid level between secondary lineages (indicated by the arrows in Fig. S9) and the corresponding TerL marker. “Within group % identity”: average within-group percent identity at the amino acid level of the alleles comprising secondary lineages. “No. of sequences”: number of sequences comprising a given secondary lineage. “No. of aa residues in alignment”: number of unambiguous residues in amino acid alignments used to calculate percent identities scores.

	Diversity of distant homologs of the markers			
	% identity compared to marker	Within group % identity	No. of sequences	No. of aa residues in alignment
**AB2**	44.4±0.8%	78.8	6	342
**HA**	44.5±1.3%	62.6	31	334
**PCA2**	45.6±1.8%	71.4	69	240
**PCA1**	48.4±1.7%	65.2	365	305
**AB1**	62.0±0.7%	85	13	387
**HB2**	64.1±1.3%	79.4	245	275

In the current study, we focused our analysis on the ‘primary’ lineage of each marker because this allowed us to simplify the complexity of phylogenetic networks by omitting divergent sublineages that may reflect additional phage-host associations, for example, associations with hosts related to other body habitats, as discussed above. Indeed, including distant homologs of the markers in our analysis did not change our findings, as discussed below. Furthermore, an in depth analysis of more divergent homologs of the TerL markers would preferably be performed bioinformatically and experimentally based on dedicated markers and degenerate primers. These dedicated markers can then be used, in turn, to search for more divergent homologs, and so on. Such an approach can possibly reveal novel shared TerL gene families

that are beyond the detection limit of MCRL using the current viral RefSeq database (Tadmor and Phillips, 2022).

Impact of reducing the percent identity threshold

Including in our analysis more distant homologs of the markers obtained by reducing the percent identity threshold did not impact our findings. For example, reducing the percent identity threshold from 70% to 55% did not have a qualitative impact on the prevalence of markers across subjects in the oral cavity or the anterior nares, with only a relatively small percent of subjects (<20% to 30%) displaying distant homologs in novel body habitats, including PCA1 and PCA2 in stool, HB2 in the posterior fornix, and HB2 and AB1 in the retroauricular crease (Fig. 2 panel c). In the case of AB2, reducing the percent identity threshold to 55% simply added six divergent but closely related alleles associated with two novel body habitats – the anterior nares and the retroauricular crease, as discussed above. In the case of HB1 and PCA2, phylogenetic analysis showed that the added TerL diversity only expanded existing sublineages, and in the case of HB2 the added TerL diversity created new sublineages, however these novel sublineages did not perturb clades discussed in the main text (Fig. S8.1). For instance, in the case of HB1, the distinct separation between oral TerL alleles and (mostly) gut TerL alleles shown in Fig. 3 panel a persisted also after addition of more distant sublineages. In the case of HB2, the clades S, T, M, and P in Fig. 3 panel b were unaffected by the more distant sublineages, the G (gut-associated) clade was divided into distinct groups (G1 and G2), with additional clades emerging, including: G3, M2, T2, T3 and PF (posterior fornix-associated clade). In the case of PCA2, only slight changes appeared compared to Fig. S12 panel b. Fig. S8.1 also shows that even after reducing the percent identity threshold to 55% no metagenomic alleles mapped to "P" clades, consistent with our observation that "P" clades were devoid of alleles from healthy individuals (see main text).P

Furthermore, reducing the percent identity threshold to 55% did not have a significant impact on marker stability analysis (data not shown). Also, importantly, when reducing the percent identity threshold to 55% we did not identify additional biases in our BLAST analysis of environmental metagenomes (Supporting Text S7), and marker presence in the environment remained generally unchanged with the exception of HB2, which displayed distant homologs that were divergent from human-associated alleles (Supporting text s6). Therefore, the main conclusions of this study were not dependent on the applied percent identity threshold.

Figure S8.1 Phylogenetic analysis of TerL markers applying a 55% identity threshold at the amino acid level. Neighbor-Net networks are shown for **a**, HB1, **b**, HB2, and **c**, PCA2 TerL alleles. Phylogenetic analysis of HB1 was based on 321 unambiguous amino acid residues obtained from 290 sequences using the optimal WAG+I+G model based on the AIC criterion with optimal α and Pinv parameters. Phylogenetic analysis of HB2 was based on 327 unambiguous amino acid residues obtained from 298 sequences using the optimal WAG+I+G model based on the AIC criterion with optimal α and Pinv parameters. Phylogenetic analysis of PCA2 was based on 353 unambiguous amino acid residues obtained from 72 sequences using the optimal WAG+I+G model based on the AIC criterion with optimal α and Pinv parameters.

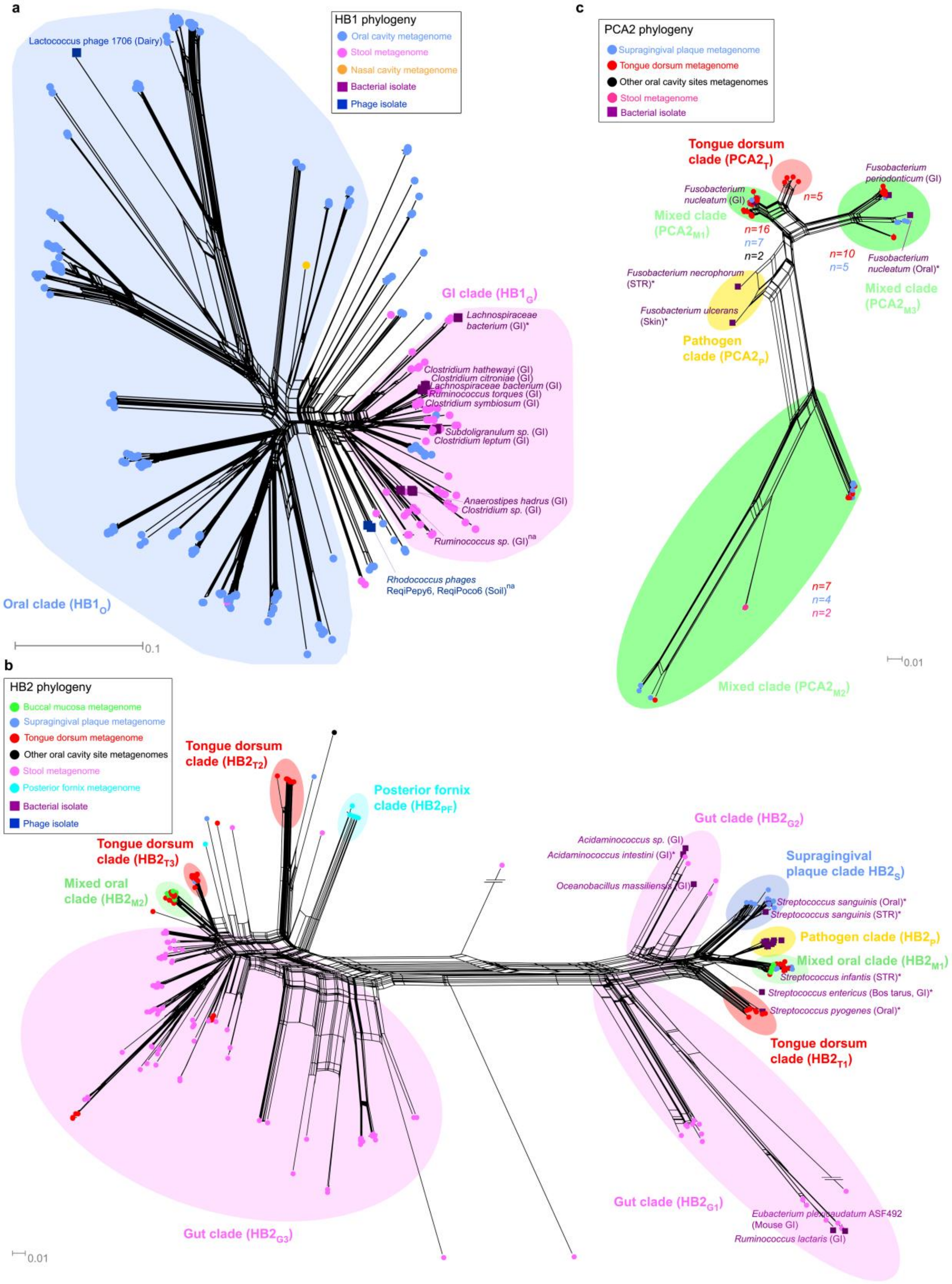

a
HB1 phylogeny
Oral cavity metagenome
Stool metagenome
Nasal cavity metagenome
Bacterial isolate
Phage isolate
Lactococcus phage 1706 (Dairy)
GI clade (HB1G)
Lachnospiraceae bacterium (GI)*
Clostridium hathewayi (GI)
Clostridium citroniae (GI)
Lachnospiraceae bacterium (GI)
Ruminococcus torques (GI)
Clostridium symbiosum (GI)
Subdoligranulum sp. (GI)
Clostridium leptum (GI)
Anaerostipes hadrus (GI)
Clostridium sp. (GI)
Ruminococcus sp. (GI)na
Rhodococcus phages
ReqiPepy6, ReqiPoco6 (Soil)na
Oral clade (HB1O)
0.1
b
HB2 phylogeny
Buccal mucosa metagenome
Supragingival plaque metagenome
Tongue dorsum metagenome
Other oral cavity site metagenomes
Stool metagenome
Posterior fornix metagenome
Bacterial isolate
Phage isolate
Tongue dorsum clade (HB2T2)
Posterior fornix clade (HB2PF)
Tongue dorsum clade (HB2T3)
Mixed oral clade (HB2M2)
Gut clade (HB2G2)
Acidaminococcus sp. (GI)
Acidaminococcus intestini (GI)*
Oceanobacillus massiliensis (GI)
Supragingival plaque clade HB2S)
Streptococcus sanguinis (Oral)*
Streptococcus sanguinis (STR)*
Pathogen clade (HB2P)
Mixed oral clade (HB2M1)
Streptococcus infantis (STR)*
Streptococcus entericus (Bos tarus, GI)*
Streptococcus pyogenes (Oral)*
Tongue dorsum clade (HB2T1)
Gut clade (HB2G1)
Eubacterium plexicaudatum ASF492 (Mouse GI)
Ruminococcus lactaris (GI)
Gut clade (HB2G3)
0.01
c
PCA2 phylogeny
Supragingival plaque metagenome
Tongue dorsum metagenome
Other oral cavity sites metagenomes
Stool metagenome
Bacterial isolate
Tongue dorsum clade (PCA2T)
Fusobacterium nucleatum (GI)
Fusobacterium periodonticum (GI)
n=5
Mixed clade (PCA2M1)
n=16
n=7
n=2
n=10
n=5
Fusobacterium nucleatum (Oral)*
Fusobacterium necrophorum (STR)*
Fusobacterium ulcerans (Skin)*
Mixed clade (PCA2M3)
Pathogen clade (PCA2P)
n=7
n=4
n=2
0.01
Mixed clade (PCA2M2)

Searching for distant homologs of the TerL markers in environmental datasets using PSI-BLAST

To confirm that our search in environmental samples was sufficiently sensitive, we used PSI-BLAST to search for potentially more divergent homologs of the TerL markers in the National Center for Biotechnology Information (NCBI) metagenomic protein database, env_nr, which includes nearly 10 million protein sequences from large environmental sequencing projects. Consistent with our BLAST-based results, we found that, with the exception of HB2, the closest hits for the markers yielded between 25% to 35% identity at the amino acid level, which is significantly lower than the similarity of TerL alleles from the HMP cohort to corresponding markers, which ranged from 79% to 98.3% identity at the amino acid level (Table S5). In the case of HB2, the closest homolog in env_nr yielded 52% identity at the amino acid level when aligned against the HB2 marker, which is consistent with our results in environmental viromes (Fig. 1 panel h), and can be compared to 85$\pm$6.8% (s.d.) identity for human associated HB2 alleles (Table S5).

S9. Phylogeny of PCR-amplified sequences

To independently confirm phylogenies that were based on HMP metagenomic sequences (Fig. 3, Fig. S11 and Fig. S12), we show that phylogenetic networks based on metagenomic sequences could be reproduced using PCR-amplified sequences. PCR-amplified sequences were obtained using degenerate primers, with each primer set targeting a different marker as described in the Materials and Methods. The resulting phylogenies are shown in Fig. S9.1, with clades labeled as indicated in Fig. 3, Fig. S11 and Fig. S12. Clades where PCR-amplified alleles were intermixed with metagenomic alleles are denoted by arrows in Fig. S12. Our analysis shows that PCR-amplified alleles obtained from specific oral sites were generally intermixed and indistinguishable from metagenomic alleles obtained from the same body sites. For example, in the case of HB1 (Fig. S9.1 panel a), PCR-amplified alleles obtained from the tongue dorsum, supragingival plaque and the buccal mucosa mapped to the $HB1_{M1}$ clade, and were intermixed with metagenomic sequences from these body sites. Similar intermixed clades were detected for the $HB1_{M2}$, $HB1_{S}$, $HB2_{M}$, HA_{M}, HA_{T2}, $PCA1_{M1}$, $PCA2_{M1}$, $PCA2_{M2}$, $PCA2_{M3}$, $AB2_{T}$, and $AB2_{S}$ clades. These results show that TerL sequences amplified directly from oral samples using our degenerate primers were capable of reproducing the phylogeny determined based on metagenomic sequences, indicating that phylogenies inferred based on metagenomic sequences were not a result of sequencing or assembly artifacts. Our analysis also shows that

none of the PCR-amplified TerL sequences mapped to “P” clades (clades highlighted in yellow in phylogenies), including “P” clades found for the HA marker (panel b), the PCA2 marker (panel c), the AB2 marker (panel d), the HB2 marker (panel e) and the PCA1 marker (panel f). Since our cohort of oral samples was obtained from carefully screened orally healthy subjects, this resul, which are hypothesized to be associated with disease-related and animal isolates.

Figure S9.1 Joint phylogeny of metagenomic alleles from the HMP cohort, PCR-amplified alleles and bacterial and phage isolates belonging to “P” clades. Degenerate primers targeting the TerL markers were applied to oral samples obtained from a cohort of orally healthy subjects. The Neighbor-Net analyses shown below were inferred using SplitsTree4 (Huson and Bryant, 2006) for six of the seven TerL markers including **a,** HB1 (6 subjects), **b,** HA (9 subjects), **c,** PCA2 (6 subjects), **d,** AB2 (3 subjects), **e,** HB2 (2 subjects), **f,** PCA1 (2 subjects). All phylogenies were inferred based on amino acid alignments that spanned the amplicon length (degenerate primers and amplicon lengths are provided in Table S3). PCR-based alleles were labeled using square symbols and a light color palette and metagenomic-based alleles were labeled using circle symbols and a dark color palette, as indicated in the legend. For reference, we included bacterial and phage isolates belonging to “P” clades, shown as purple and black squares, respectively. Clades where PCR-amplified alleles were intermixed with metagenomic alleles are denoted by arrows.

Phylogenetic analysis of HB1 was based on 81 unambiguous amino acid residues obtained from 231 sequences using the optimal JTT+G model based on the AIC criterion with an optimal α parameter. Phylogenetic analysis of HB2 was based on 107 unambiguous amino acid residues obtained from 107 sequences using the optimal WAG+G model based on the AIC criterion with an optimal α parameter. Phylogenetic analysis of PCA1 was based on 94 unambiguous amino acid residues obtained from 106 sequences using the optimal JTT+I+G model based on the AIC criterion with optimal α and Pinv parameters. Phylogenetic analysis of PCA2 was based on 60 unambiguous amino acid residues obtained from 179 sequences using the optimal JTT+G model based on the AIC criterion with an optimal α parameter. Phylogenetic analysis of HA was based on 82 unambiguous amino acid residues obtained from 246 sequences using the optimal JTT+G model based on the AIC criterion with an optimal α parameter. Phylogenetic analysis of AB2 was based on 139 unambiguous amino acid residues obtained from 68 sequences using the optimal WAG+G model based on the AIC criterion with an optimal α parameter.

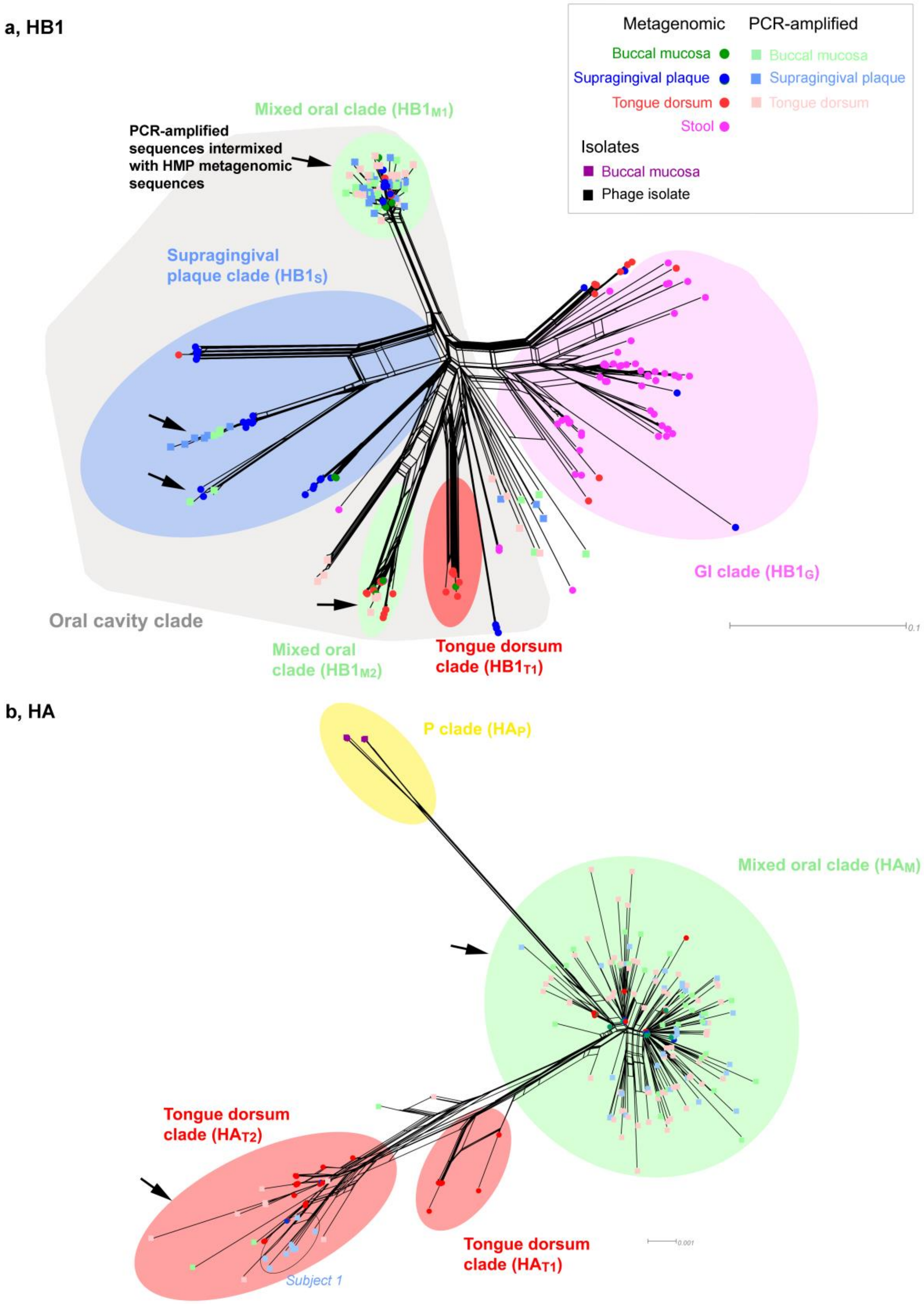
a, HB1
Metagenomic
PCR-amplified
Buccal mucosa
Supragingival plaque
Tongue dorsum
Stool
Buccal mucosa
Supragingival plaque
Tongue dorsum
Isolates
Buccal mucosa
Phage isolate
Mixed oral clade (HB1M1)
PCR-amplified sequences intermixed with HMP metagenomic sequences
Supragingival plaque clade (HB1S)
GI clade (HB1G)
Oral cavity clade
Mixed oral clade (HB1M2)
Tongue dorsum clade (HB1T1)
0.1
b, HA
P clade (HAP)
Mixed oral clade (HAM)
Tongue dorsum clade (HAT2)
Tongue dorsum clade (HAT1)
Subject 1
0.001

c, PCA2

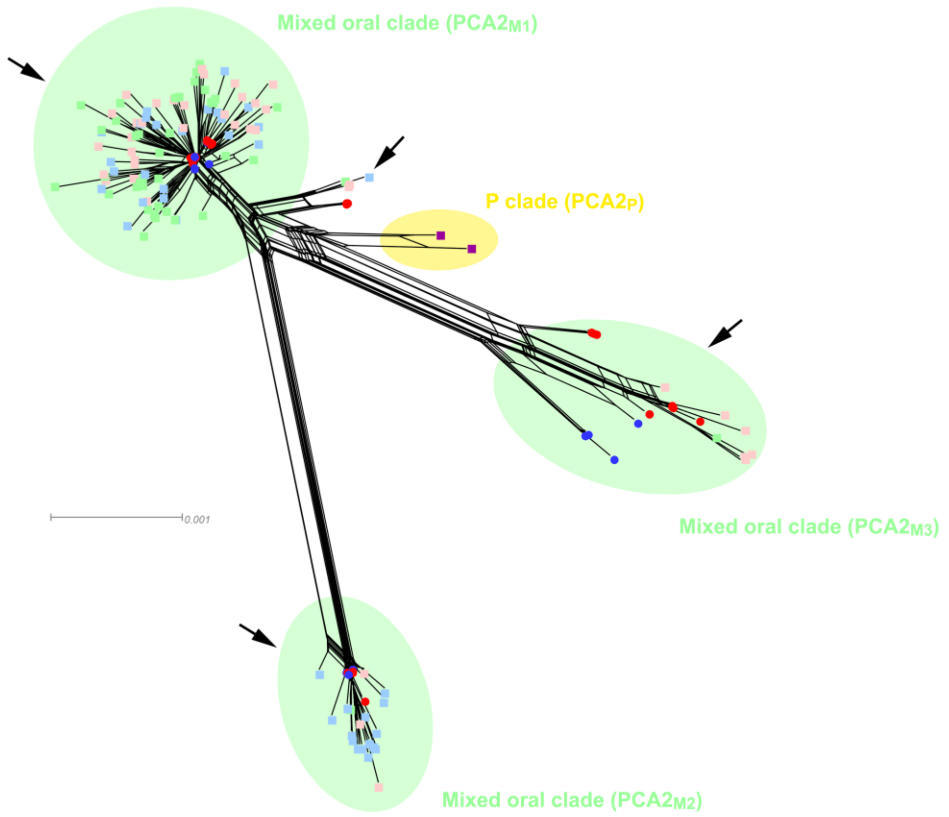
Mixed oral clade (PCA2M1)
P clade (PCA2P)
Mixed oral clade (PCA2M3)
0.001
Mixed oral clade (PCA2M2)

d, AB2

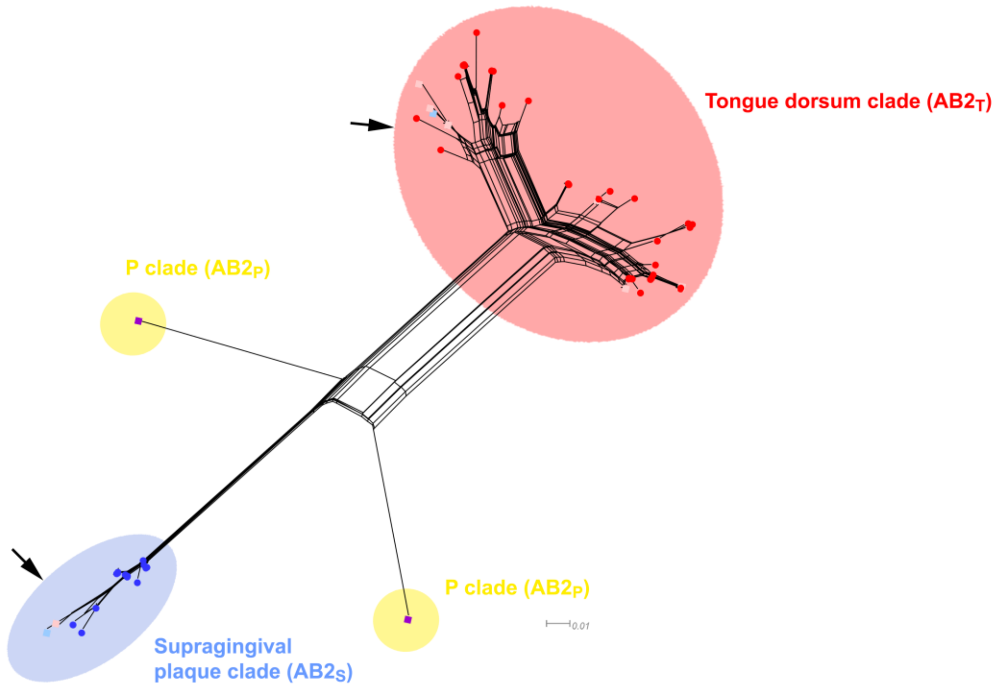
Tongue dorsum clade (AB2T)
P clade (AB2P)
P clade (AB2P)
0.01
Supragingival
plaque clade (AB2S)

e, HB2

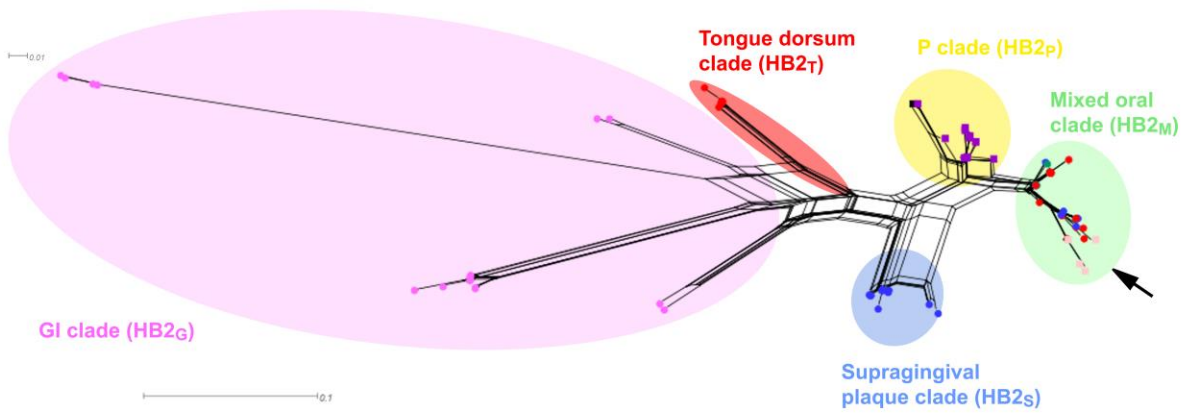
0.01
Tongue dorsum clade (HB2T)
P clade (HB2P)
Mixed oral clade (HB2M)
GI clade (HB2G)
Supragingival plaque clade (HB2S)
0.1

f, PCA1

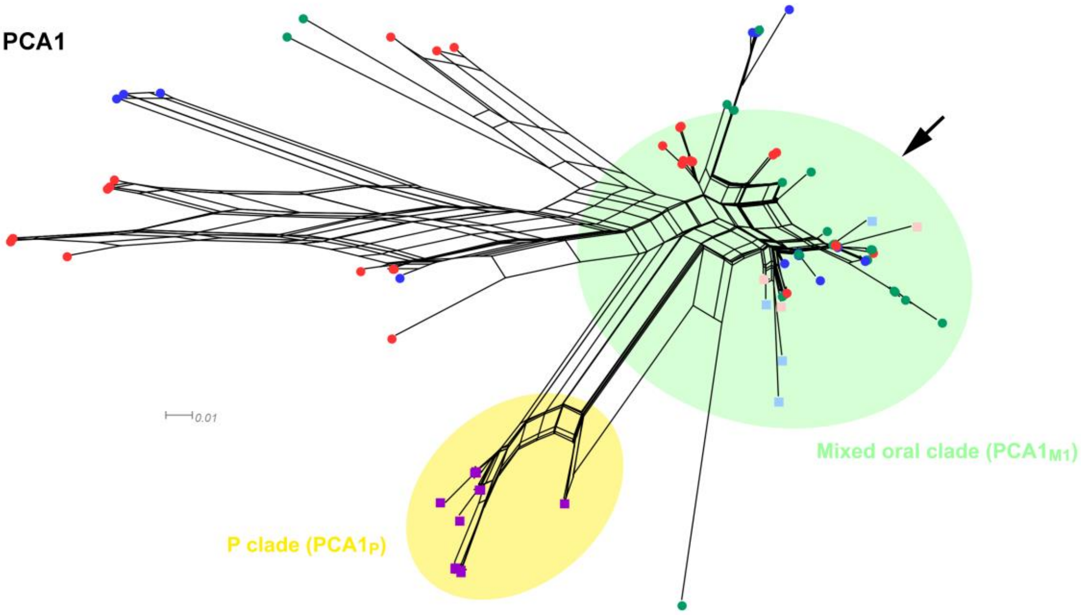
0.01
Mixed oral clade (PCA1M1)
P clade (PCA1P)

SUPPORTING FIGURES

Figure S1. Overview of methodology and analysis. **a,** Overview of methodology for identifying shared TerL markers in humans. **b,** Association of the TerL markers with pfams and COGs and phylogenetic characterization of the TerL markers. **c,** Determination of the prevalence of the TerL markers in humans based on the HMP oral cohort, oral viromes, and an in-house cohort of oral samples interrogated by targeted sequencing; **d,** Determination of the prevalence of the TerL markers in natural environments based on an analysis of environmental metagenomes, environmental viromes, and sequenced bacterial and phage isolates. **e,** Assessment of the functionality of metagenomic and PCR-amplified sequences belonging to shared TerL gene families. **f,** Investigation of the ecology of shared TerL gene families in the human microbiome in terms of bacterial hosts, lifestyle, spatial distribution, temporal dynamics, and phylogeny.

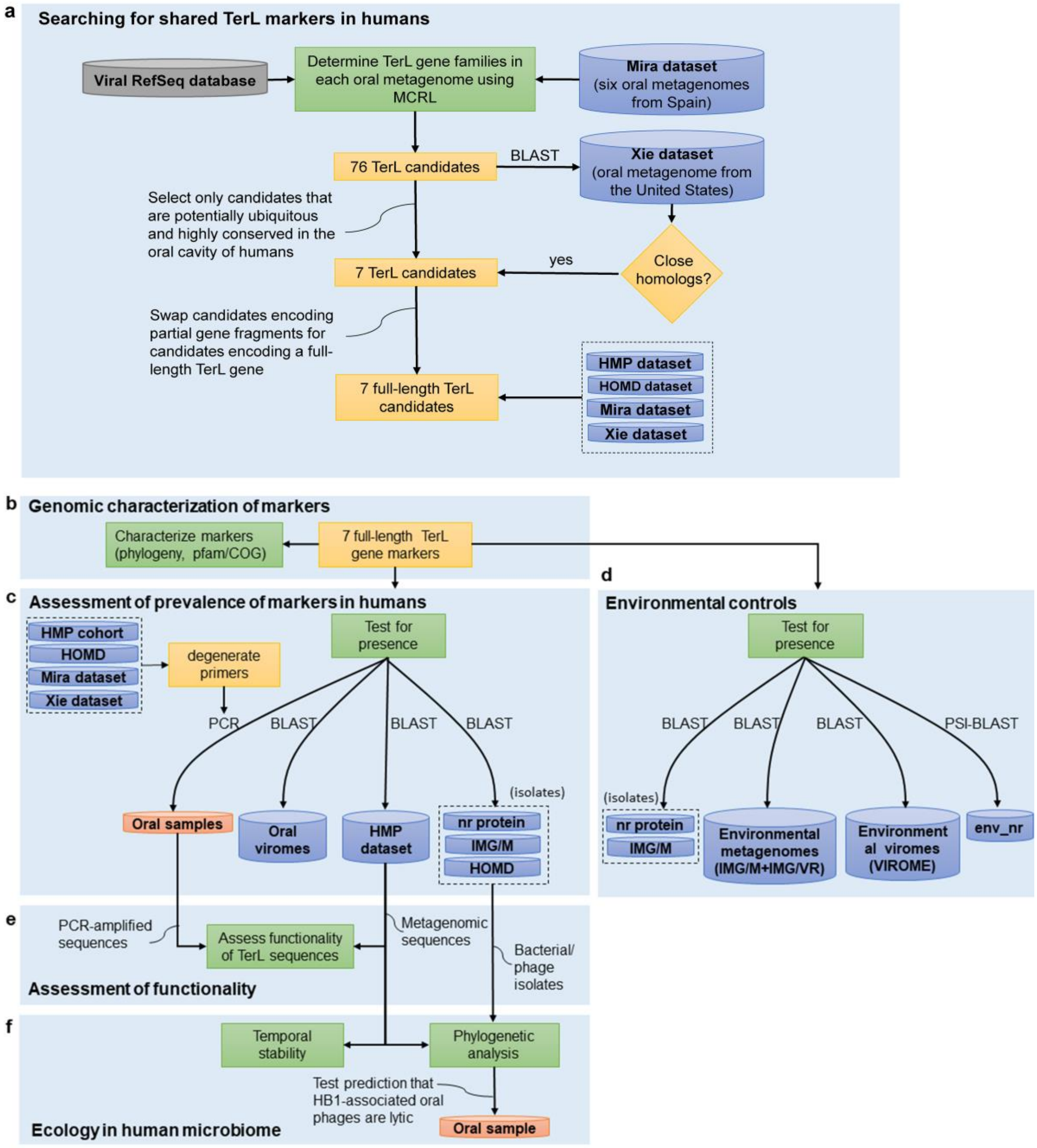

Figure S2. Summary of bioinformatic approach to identify shared TerL markers. a, The method to identify ubiquitous and conserved TerL markers in humans comprised the following steps: (1) the MCRL data mining algorithm was initially applied determine a nonredundant set of putative viral RefSeq genes families present in each of the six dental plaque metagenomes in the Mira study representing humans with varying degree of oral hygiene (see below), (2) putative viral RefSeq genes families were screened for TerL genes, (3) candidates corresponding to short alignments and/or candidates that had few homologs in the metagenomes were eliminated, (4) candidates that had close homologs in an oral metagenome from an independent study from another region of the world were retained, (5) remove redundant candidates (i.e., candidates homologous to other candidates). The final set of candidate TerL markers spanned only a fragment of the length of a TerL gene because the contigs in the Mira dataset were significantly shorter than the average length of a TerL gene (336±167 nt (s.d.) versus ~1650 nt). Therefore, in order for the markers to be effective, each TerL candidate was replaced with a closely related sequence that spanned the entire length of a TerL gene. **b, Prevalence of full-length TerL markers in the Mira and Xie datasets.** The heat map shows the maximum percent identity at the amino acid level obtained when BLASTing the indicated full-length TerL marker against the indicated metagenome, considering only alignments exceeding 120 amino acids (see Material and Methods for criteria used to determine the alignment length threshold). All alignments were performed on amino acid sequences. Cells highlighted with a blue frame indicate the metagenome from which the TerL candidate was first identified.

Naming convention for the Mira metagenomes

Subjects in the Mira study belonged to one of three oral hygiene groups (Belda-Ferre et al., 2012): good oral hygiene (orally **h**ealthy subjects who never developed caries in their lives) – denoted by the prefix '**H**', mediocre oral hygiene (individuals that had been regularly treated for **p**ast **c**aries and had a low number of active caries (1 and 4) at the moment of sampling) – denoted by the prefix '**PC**', and poor oral hygiene (individuals who had a high number of **a**ctive caries (8 and 15) and poor oral hygiene) – denoted by the prefix '**A**'. The Mira study included six subjects, two subjects per oral hygiene group, denoted by the postfix 'A' or 'B'. Metagenomes in the Mira study were hence labeled according to the oral hygiene group followed by the subject label. For example, the metagenome labeled 'HA' was obtained from subject A belonging to the good oral hygiene group (H).

Naming convention for the markers

Markers were labeled according to the metagenome from the Mira study in which they were identified, followed by an index counting the TerL candidate discovered in the given metagenome. For example, the marker 'HB2' is the second candidate (index=2) identified in the 'HB' metagenome. This naming convention was useful for correlating marker prevalence in the oral cavity of orally healthy humans with the degree of oral hygiene corresponding to the metagenome where the marker was discovered (see Supporting Text S5 for a statistical analysis of this correlation).

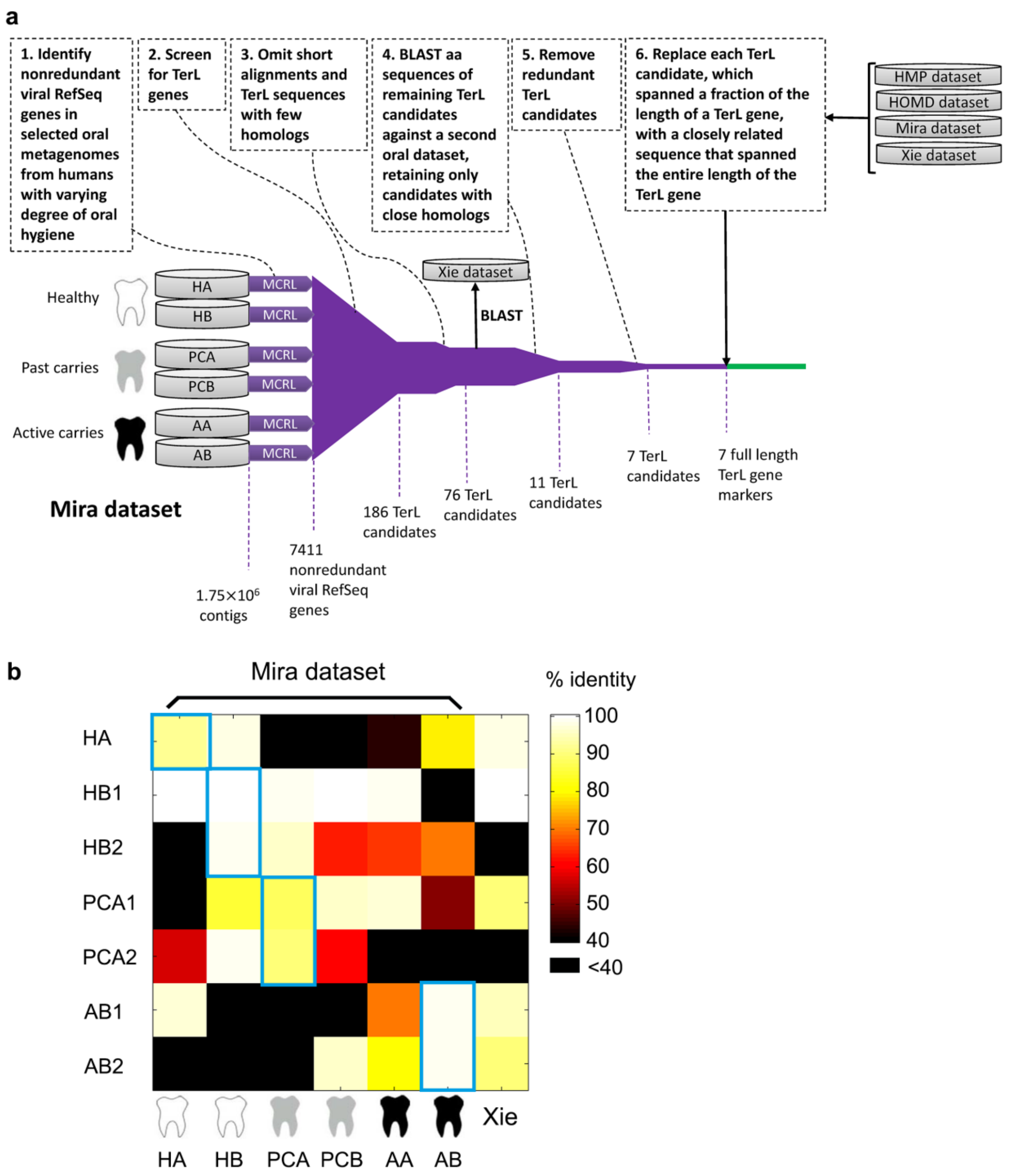
a
1. Identify nonredundant viral RefSeq genes in selected oral metagenomes from humans with varying degree of oral hygiene
2. Screen for TerL genes
3. Omit short alignments and TerL sequences with few homologs
4. BLAST aa sequences of remaining TerL candidates against a second oral dataset, retaining only candidates with close homologs
5. Remove redundant TerL candidates
6. Replace each TerL candidate, which spanned a fraction of the length of a TerL gene, with a closely related sequence that spanned the entire length of the TerL gene
HMP dataset
HOMD dataset
Mira dataset
Xie dataset
Xie dataset
BLAST
Healthy
Past carries
Active carries
HA
HB
PCA
PCB
AA
AB
MCRL
Mira dataset
1.75×10^6 contigs
7411 nonredundant viral RefSeq genes
186 TerL candidates
76 TerL candidates
11 TerL candidates
7 TerL candidates
7 full length TerL gene markers
b
Mira dataset
% identity
100
90
80
70
60
50
40
<40
HA
HB1
HB2
PCA1
PCA2
AB1
AB2
Xie
HA
HB
PCA
PCB
AA
AB

Figure S3. Conserved functional signatures in TerL lineages. For five of the seven markers for which 3D-stucture based domain models could be fitted (Marchler-Bauer et al., 2016), we found that functional signatures typical for terminase genes appeared intact across nearly all corresponding HMP subjects. In the case of four markers for which degenerate primers targeted regions containing functional signatures (HA, HB2, PCA1 and PCA2), functional signatures were strictly conserved in 96.5% of 289 PCR-amplified sequence**.** The figure below shows sequence alignments showing functional signatures of the N-terminal adenosine triphosphatase (ATPase) domain and C-terminal nuclease domain of TerL lineages based on HMP metagenomic sequences and PCR-amplified sequences. Alignments are shown for the following markers: **a** HB2, **b** PCA2, **c** HA, **d** HB1, and **e** PCA1. Alignments include (i) TerL sequences of phages with published functional signatures (Rao and Feiss, 2008; Sun et al., 2008), (ii) homologous TerL sequences that belong to phages that have 3D-stucture based domain models of their terminase genes in the conserved domain database (CDD) (Marchler-Bauer et al., 2016). These phages include for HA, HB1, HB2, and PCA2: HK97 and Lactococcus virus c2 (Lubbers et al., 1995), and for PCA1: SPP1 (Yasbin and Young, 1974), and T5, which are all known functional phages. For PCA1, prophage Lj928 was also included, which is a noninducible but complete prophage of *Lactobacillus johnsonii* NCC 533 (Ventura et al., 2004), (iii) all TerL sequences from the HMP cohort homologous to the indicated markers selecting one sequence per subject, and (iv) PCR-amplified TerL sequences obtained using degenerate primers targeting the markers.

For each marker, the N-terminal domain containing the conserved ATPase center of the TerL gene and C-terminal domain containing the nuclease center of the TerL gene were separately aligned using MUSCLE (Edgar, 2004) in MEGA (Tamura et al., 2013), and then combined, using an RPS BLAST alignment against the 3D-stucture based domain models in CDD to identify the flexible hinge motif combining both domains. Numbers in brackets correspond to aligned residues not shown. Stars indicate conserved residues in human-associated sequences (top track) and in the multiple alignment (bottom track). Dots indicate end of available sequence. The positions of the degenerate primers are also noted at the top of each alignment (PCR-amplified sequences are shown without primer regions). This figure demonstrates that human associated TerL sequences homologous to the indicated markers exhibit conserved functional signatures typical for terminase enzymes.
Functional signatures were identified by aligning in CDD TerL sequences of phages with published functional signatures (HK97 for HB2, PCA2, HA, HB1 and SPP1 for PCA1) (Rao and Feiss, 2008; Sun et al., 2008) against pfams corresponding to the markers using RPS BLAST (pfams are listed in Table S3). Conserved functional signatures for the N-terminal ATPase center include: an adenine binding motif YQ (pink), a Walker A motif G/A-XXXXGK(T/S) (purple), a Walker B motif ZZZZD where Z represents a hydrophobic amino acid (blue), a catalytic carboxylate group motif (usually) Glu (orange), and an ATPase coupling motif (T/S-G/A-T/S (N)) (green). Conserved functional signatures for the C-terminal nuclease center are a triad of Asp/Glu residues (red). The flexible hinge motif connecting the N-terminal domain and the C-terminal domain is shown in brown.

Panel a: Functional signatures for HB2 were conserved in all TerL sequences (in total 89 to 99 sequences) collected from 47 subjects, and include: (1) an adenine binding motif, with Tyr replaced by Trp, a biochemically similar amino acid, (2) a Walker A motif (purple), with the first residue replaced by an Ile residue matching the Ile residue found in HK97 at this position (and similar to the Val residue in Lactococcus virus c2 at this position), (3) a conserved Walker B motif (blue), (4) a catalytic carboxylate group motif - Glu (orange), (5) a putative ATP coupling motif (green) with residues matching those of Lactococcus virus c2. Using degenerate

primers we also retrieved seven additional alleles collected from two subjects and found that all sequences conserved the ATP coupling motif. Finally, (6) a catalytic Asp/Glu residue (here an Asp residue shown in red) (Rao and Feiss, 2008; Sun et al., 2008).

Panel b: Functional signatures for PCA2 were conserved or exhibited neutral substitutions in all TerL sequences (in total 57 to 68 sequences) collected from 33 subjects, and include: (1) an adenine binding motif with the first residue matching the Phe residue found in HK97 at this position, which is biochemically similar to Tyr found in the adenine binding motif at this position, (2) a Walker A motif (purple), with the first residue a Val, matching the Val residue found in Lactococcus virus c2 at this position and similar to the Ile residue found in HK97 at this position, (3) a Walker B motif (blue), which in the HMP dataset was conserved except for the fourth residue, which is not hydrophobic but was nevertheless a strictly conserved Cys residue in 42 subjects across 68 alleles and which is a neutral substitution compared to the hydrophobic Ala residue, and except for one sequence deviating at the second position but encoding a neutral Ala to Thr substitution. Moreover, non-hydrophobic amino acids in Walker B motifs also occur in functional phages. For example, T4 has a Tyr in the third position and λ has a Tyr in the fourth position (Rao and Feiss, 2008). Furthermore, using degenerate primers that spanned the first 3 amino acids of the Walker B motif we retrieved 106 sequences collected from seven subjects and found that all sequences conserved the Walker B motif except for seven sequences (6.6%) deviating in the second position, which are nevertheless swapped with similar or neutral substitutions with respect to the expected hydrophobic residue. In both the HMP dataset and the alleles we retrieved using degenerate primers the motif we found matched the HK97 motif in nearly all sequences. (4) A catalytic carboxylate group motif - Glu (orange), (5) a putative ATP coupling motif (green) with residues matching those of Lactococcus virus c2, and (6) a catalytic Asp/Glu residue (here an Asp residue shown in red).

Panel c: Functional signatures for HA were conserved, exhibited neutral substitutions or (at one position) exhibited a non-neutral but strictly conserved substitution in all TerL sequences (in total 92 to 105 TerL sequences) collected 46 subjects, and include: (1) a conserved adenine binding motif, (2) a Walker A motif (purple), with the first residue replaced by a Leu residue similar to the Ile/Val residues found in HK97 and Lactococcus virus c2 at this position, and an Asn residue at the last position, which is biochemically similar to the Thr/Ser residues found at this position in the Walker A motif, (3) a Walker B motif (blue), except for the first and third residues which were not hydrophobic but nevertheless strictly conserved in 54 subjects across 105 alleles, (4) a catalytic carboxylate group motif - Glu (orange), (5) a putative ATP coupling motif (green) with the first residue replaced by an Asn, which is biochemically similar to the Thr/Ser residues found at this position in the C-motif. Furthermore, using degenerate primers that span this motif we retrieved 168 sequences collected from nine subjects and found that all sequences conserved the C-motif. (6) A catalytic Asp/Glu residue (here an Asp residue shown in red).

Panel d: Functional signatures for HB1 were conserved or exhibited neutral substitutions in all TerL sequences (in total 367 to 408 sequences) collected 80 subjects, and include: (1) a Walker A motif (purple), with the first residue replaced by Ile/Val/Leu either matching or similar to the Ile/Val residues found in HK97 and Lactococcus virus c2 at this position, and the sixth residue replaced in >99% of sequences by an Ala residue, matching the Ala residue found in Lactococcus virus c2 at this position (a neutral change with respect to the Gly at this position in the Walker A motif), (2) a Walker B motif (blue) except for one residue encoding a conserved Thr residue in 87 subjects across 408 alleles – a neutral change compared to hydrophobic residues such as Val and Ala, a second residue ranging between a hydrophobic

residue and a residue that is a positive (positive BLOSUM62 score) or neutral substitution compared to hydrophobic residues, and with the first position encoding a hydrophobic residue in >99% of sequences, and a positive or neutral substitution in <1% of sequences, (3) a catalytic carboxylate group motif - Glu (orange), (4) a putative ATP coupling motif (green) where in some sequences the first amino acid is replaced with a Val/Ile/Met residue matching or similar to the Ile residue found in HK97 and Lactococcus virus c2 at this position, and with the second residue replaced with Ser/Thr matching the Ser/Thr residues found in HK97 and Lactococcus virus c2 at this position, and (5) a catalytic Asp/Glu residue (here an Asp residue shown in red).

Panel e: Functional signatures for PCA1 were conserved, exhibited neutral substitutions or (at one position) exhibited a non-neutral but strictly conserved substitution in all 94 TerL sequences collected from 55 subjects, and include: (1) a Walker A motif (purple), with the sixth residue replaced by a Lys, which although biochemically different from the Gly/Ala expected at this position, was strictly conserved across all 94 alleles collected from 55 subjects, (2) a Walker B motif (blue) with the Asp residue replaced by the biochemically similar Glu residue, (3) a catalytic carboxylate group motif - Glu (orange), (4) a putative ATP coupling motif (green) with the second residue matching the Phe residue found in *Lactobacillus johnsonii*. Using degenerate primers we also retrieved nine additional alleles collected from two subjects and found that 7 out of 9 sequences conserved the ATP coupling motif, one sequence contained a Ser residue instead of the Asn residue at the third position, which are biochemically similar residues, and one sequence contained a Leu residue instead of the Phe residue at the second position, a neutral substitution. Finally, (5) two catalytic Asp/Glu residues residue (here Asp residues shown in red).

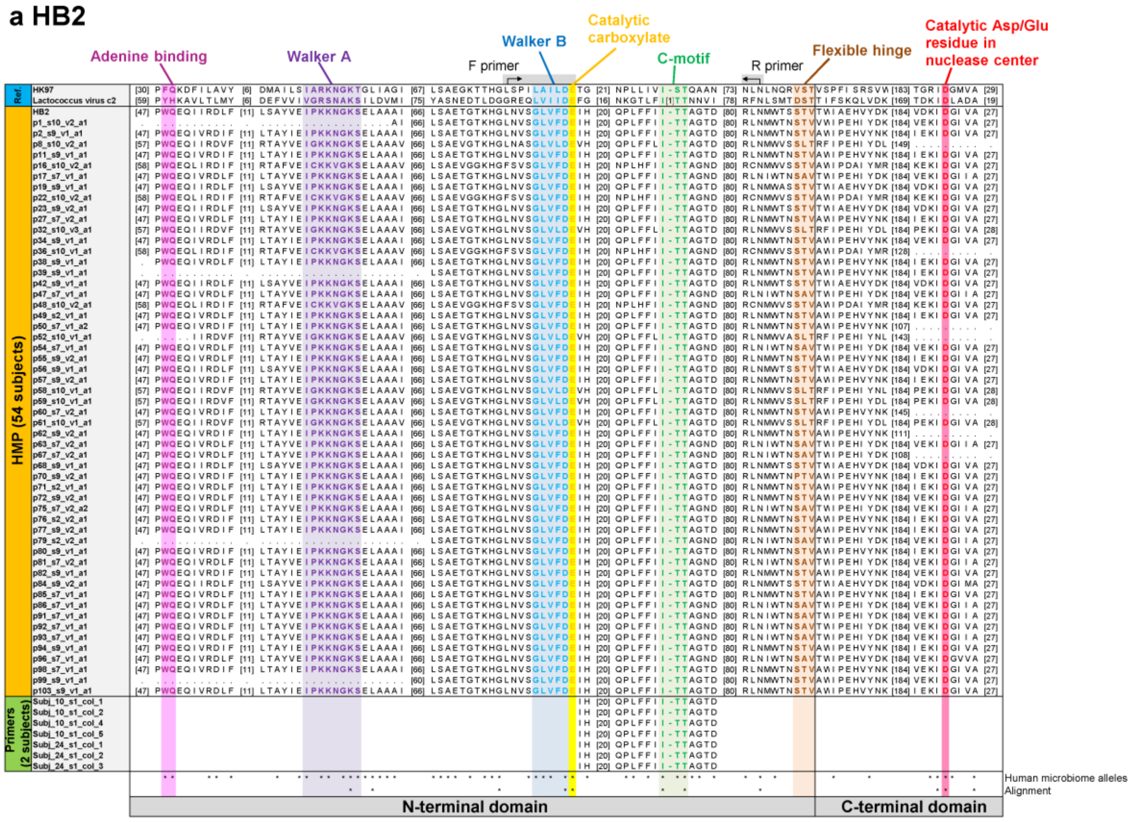

b PCA2

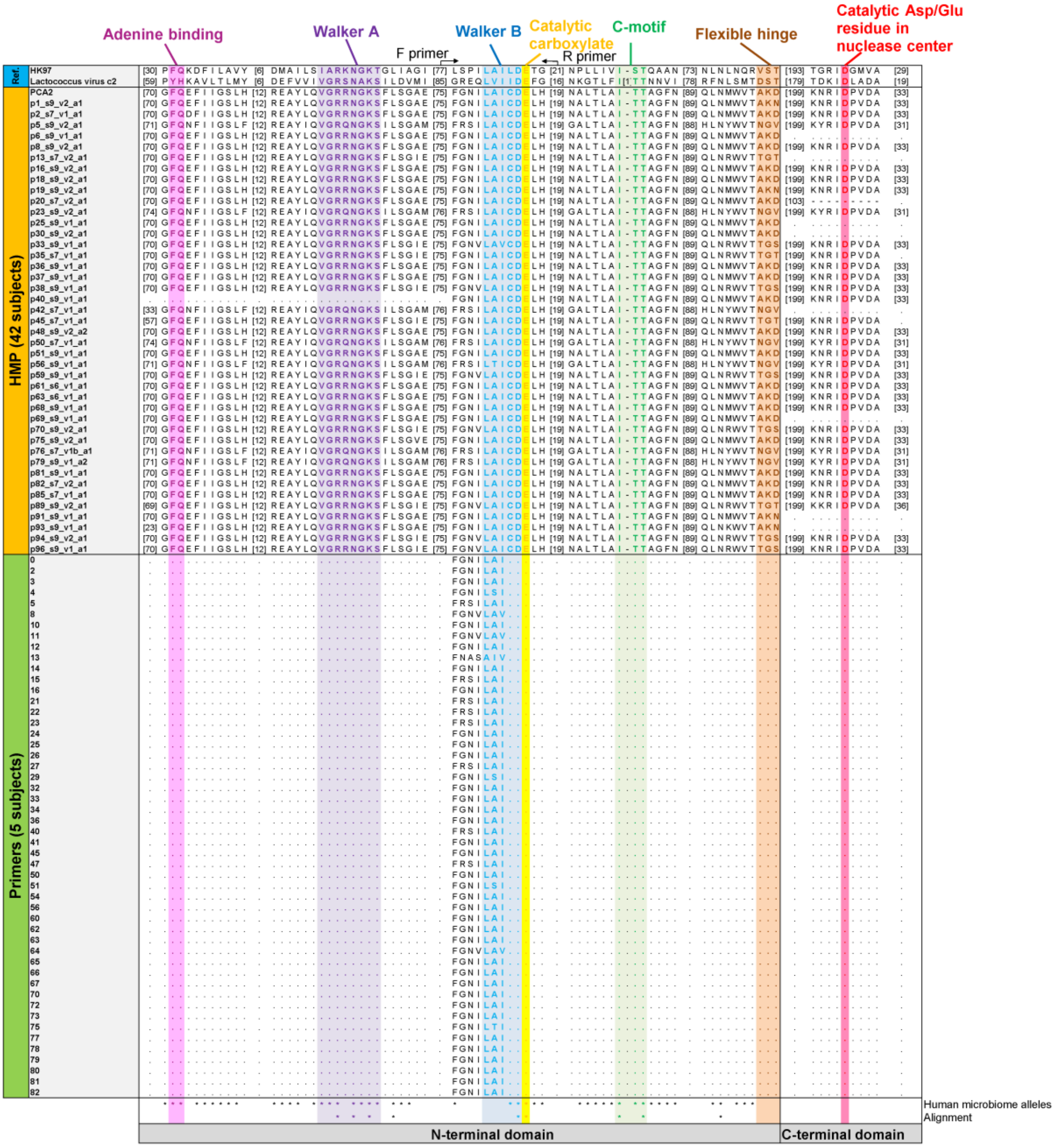
Adenine binding
Walker A
Walker B
Catalytic carboxylate
C-motif
Flexible hinge
Catalytic Asp/Glu residue in nuclease center
F primer
R primer
Ref.
HK97
Lactococcus virus c2
HMP (42 subjects)
PCA2
Primers (5 subjects)
Human microbiome alleles Alignment
N-terminal domain
C-terminal domain

c HA

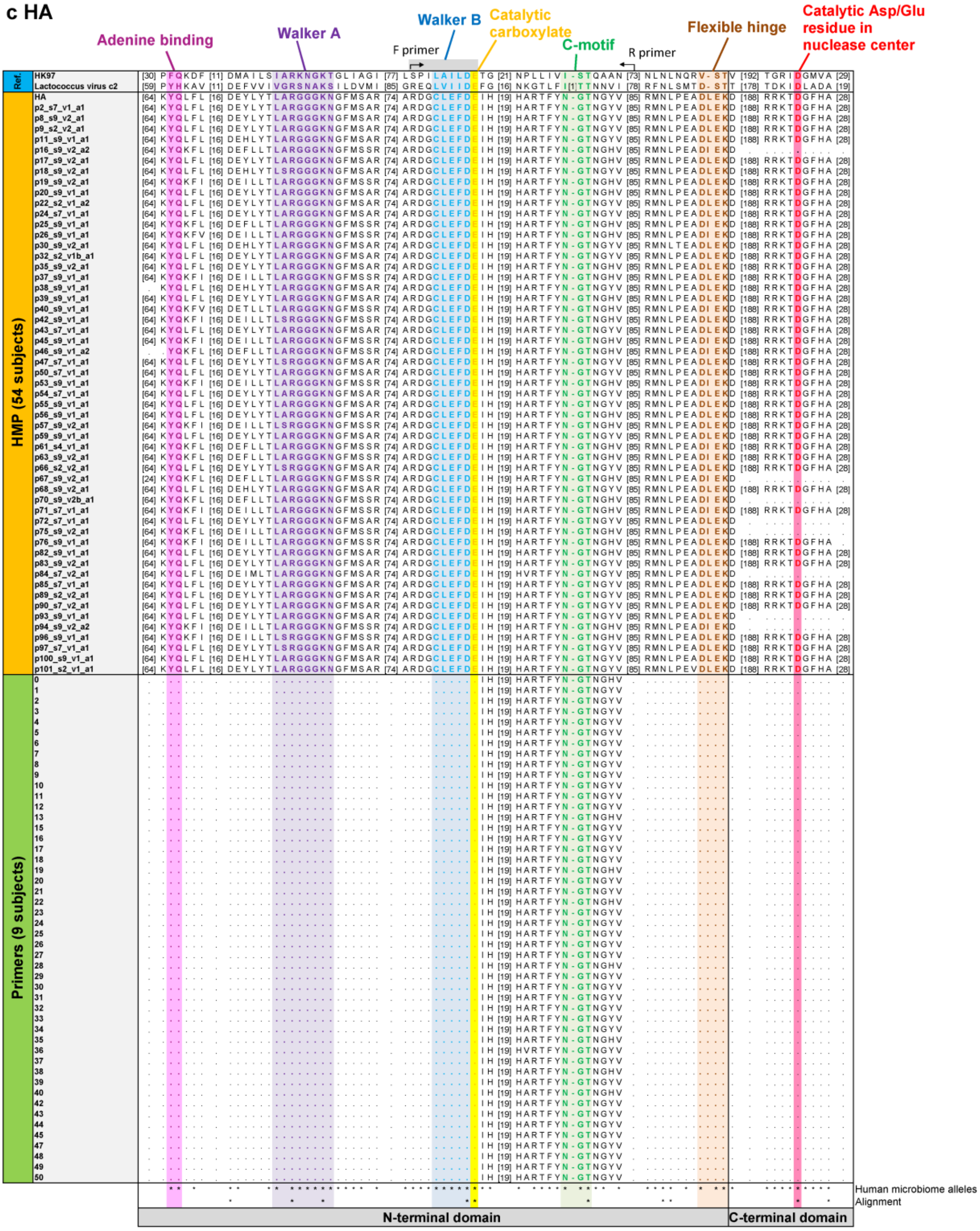
Adenine binding
Walker A
Walker B
Catalytic carboxylate
C-motif
Flexible hinge
Catalytic Asp/Glu residue in nucleast center
F primer
R primer
Ref.
HMP (54 subjects)
Primers (9 subjects)
Human microbiome alleles
Alignment
N-terminal domain
C-terminal domain

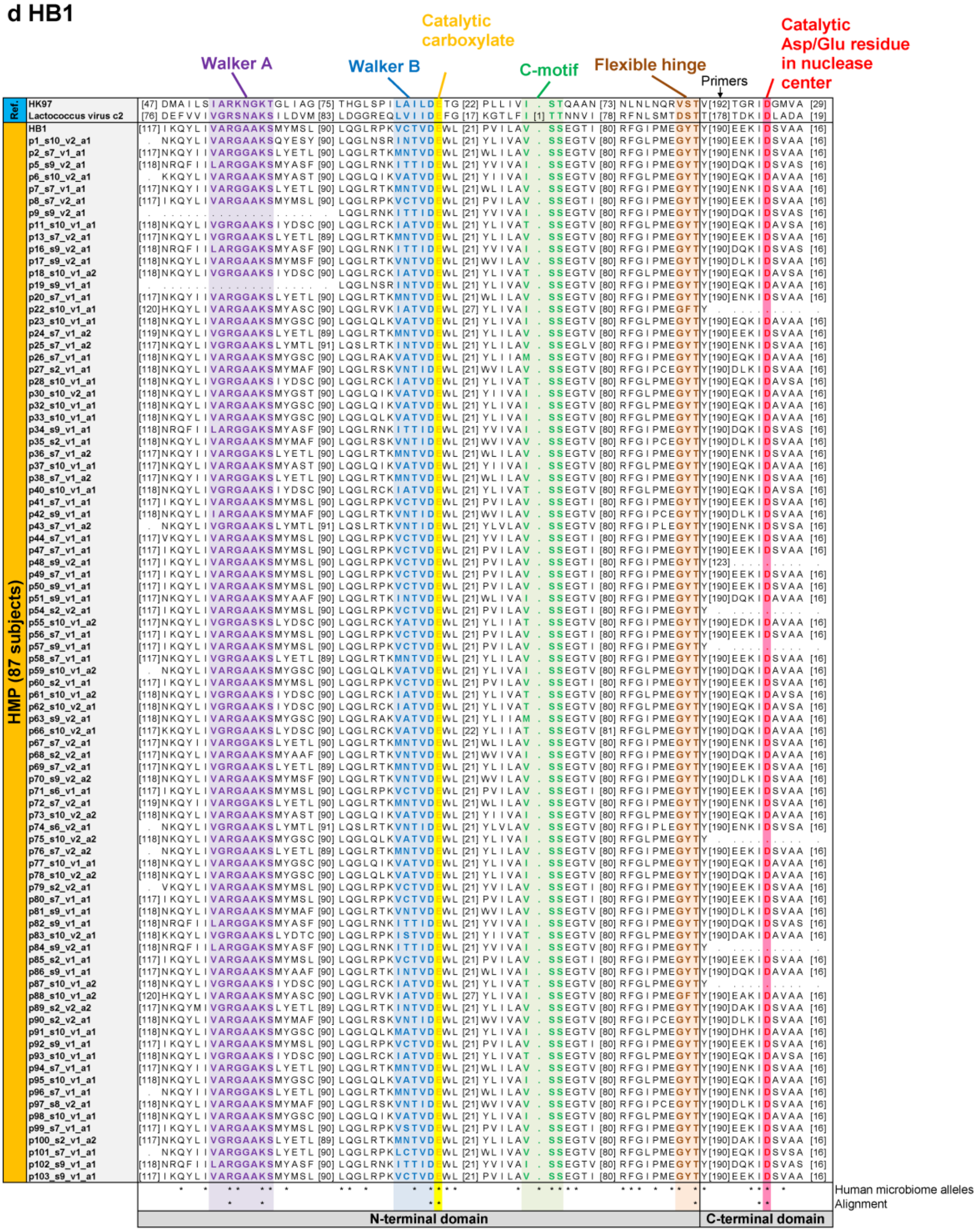
d HB1
Catalytic carboxylate
Walker A
Walker B
C-motif
Flexible hinge
Primers
Catalytic Asp/Glu residue in nuclease center
Ref.
HK97
Lactococcus virus c2
HB1
HMP (87 subjects)
Human microbiome alleles
Alignment
N-terminal domain
C-terminal domain

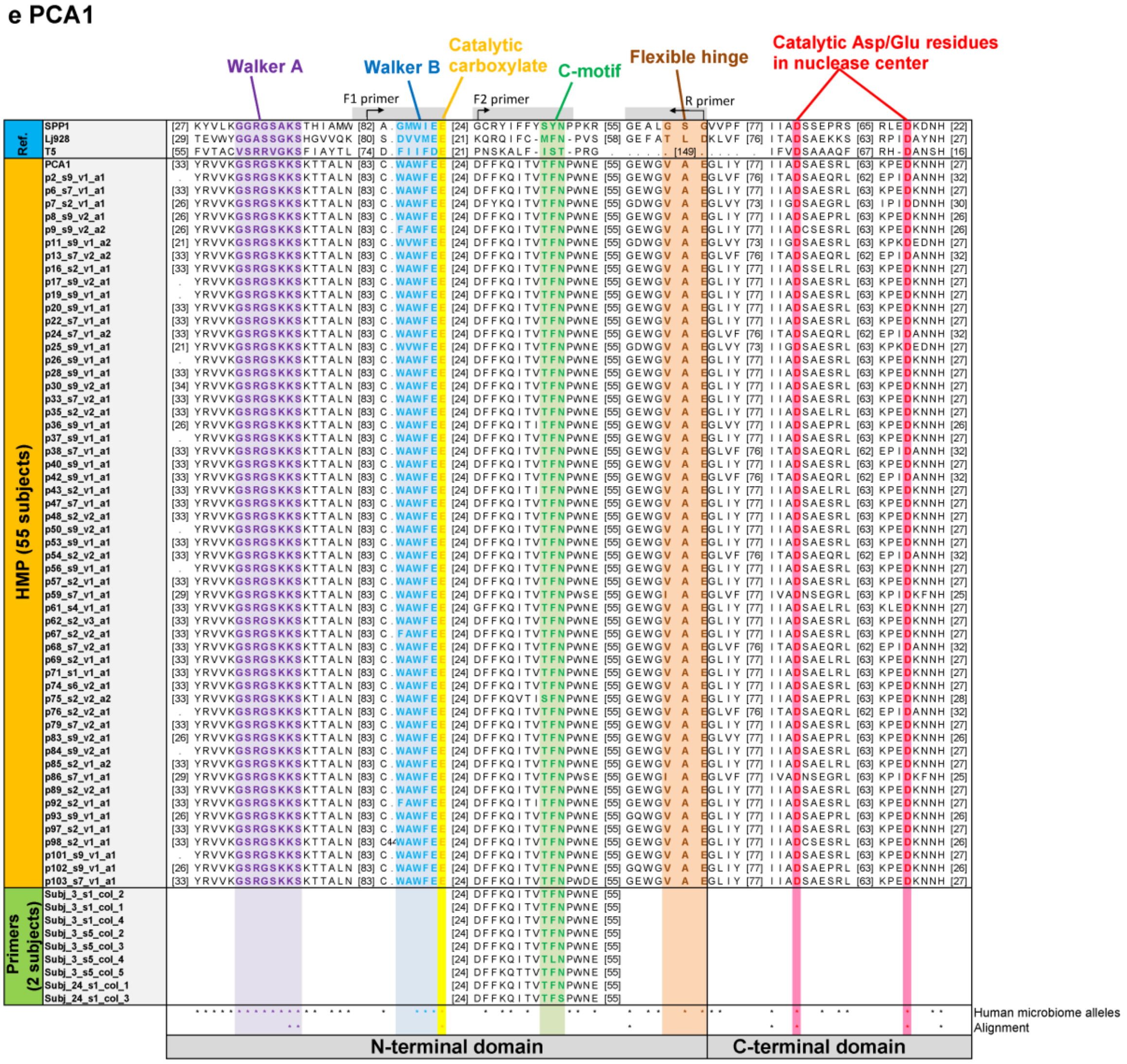
e PCA1
Walker A
Walker B
Catalytic carboxylate
C-motif
Flexible hinge
Catalytic Asp/Glu residues in nuclease center
F1 primer
F2 primer
R primer
Ref.
HMP (55 subjects)
Primers (2 subjects)
Human microbiome alleles
Alignment
N-terminal domain
C-terminal domain

Figure S4. Phylogenetic placement of TerL markers. TerL proteins recognize DNA for packing and have nuclease activity responsible for creating the ends of viral DNA (Catalano et al., 1995). Previous phylogenetic analysis by Casjens *et al.* of TerL protein sequences showed that TerL genes with similar enzymatic end-generation functions cluster together forming at least eight robust phylogenetic groups (Casjens et al., 2005). To characterize our seven TerL markers in the context of TerL genes found in nature we performed a maximum likelihood phylogenetic analysis of the amino acid sequence of the TerL markers along with 55 additional TerL genes previously analyzed by Casjens et al. Nodes shown in the tree were color coded based on the phylogenetic classification proposed by Casjens et al. Of the eight groups identified by Casjens et al., we were able to reproduce seven, with the group of “Mu-like headful” TerL genes omitted from our analysis because it destabilize our tree, reducing bootstrap support of other clades.

Our phylogenetic analysis showed that HA, PCA2, HB2 and AB2 grouped with 3’-extended COS ends sequences, and PCA1 grouped with P22-like headful sequences, in agreement with the associated pfam/COGs for these markers (Table S3). When omitting the GTA headful group, all members of the 3’-extended COS ends group (light blue) coalesced to one clade with 74% bootstrap support and with HA, HB2, AB2 and PCA2 grouping within this clade with higher internal bootstrap support (data not shown). HB1 and AB1 did not group with any of the TerL phylogenetic groups established by Casjens et al. and since their inclusion resulted in an unstable phylogeny they were excluded from our phylogenetic analysis. Indeed, Casjens et al. notes that the eight groups mapped by the authors do not encompass all known terminases. For example, 3’ cohesive end phages and certain headful packaging phages were left out by Casjens et al. since they diverged too greatly from most TerL enzymes and their inclusion was not sufficiently supported (Casjens et al., 2005).

The maximum likelihood analysis included 130 unambiguous amino acid residues, with the optimal amino acid substitution model determined by ProtTest3.428 (Darriba et al., 2011) to be WAG (Whelan and Goldman, 2001) +F+G according to the AIC criterion allowing for 48 model combinations supported by MEGA (Tamura et al., 2013). The bootstrap consensus tree was inferred from 1000 replicates, with branches corresponding to partitions reproduced in less than 50% bootstrap replicates are collapsed. A discrete Gamma distribution was used to model evolutionary rate differences among sites (5 categories (+G, parameter = 9.0279)).

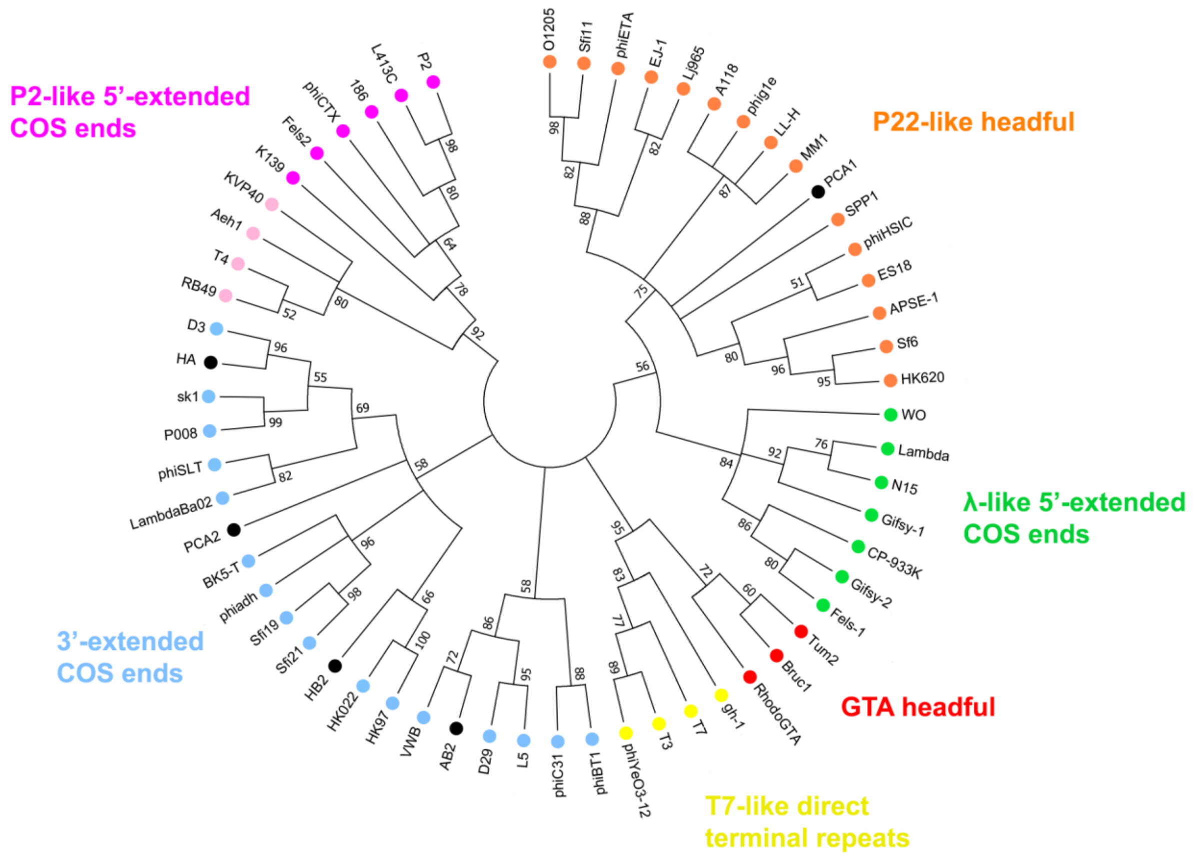
P2-like 5'-extended COS ends
P22-like headful
λ-like 5'-extended COS ends
GTA headful
T7-like direct terminal repeats
3'-extended COS ends
O1205
Sfi11
phiETA
EJ-1
Lj965
A118
phig1e
LL-H
MM1
PCA1
SPP1
phiHSIC
ES18
APSE-1
Sf6
HK620
WO
Lambda
N15
Gifsy-1
CP-933K
Gifsy-2
Fels-1
Tum2
Bruc1
RhodoGTA
gh-1
T7
T3
phiYeO3-12
phiBT1
phiC31
L5
D29
AB2
VWB
HK97
HK022
HB2
Sfi21
Sfi19
phiadh
BK5-T
PCA2
LambdaBa02
phiSLT
P008
sk1
HA
D3
RB49
T4
Aeh1
KVP40
K139
Fels2
phiCTX
186
L413C
P2

Figure S5. Annotation of the HMP contigs that encode the full-length TerL markers. The local genomic neighborhood of the full-length TerL markers is shown in the HMP contigs from which they were obtained. This figure shows that the full-length TerL genes were encoded in a region containing other phage genes. TerL genes were always adjacent to portal protein genes, an exclusive organization in tailed phages and prophages (Casjens, 2003). The identifiers of the contigs shown below and their corresponding metagenomes is provided in Table S3.

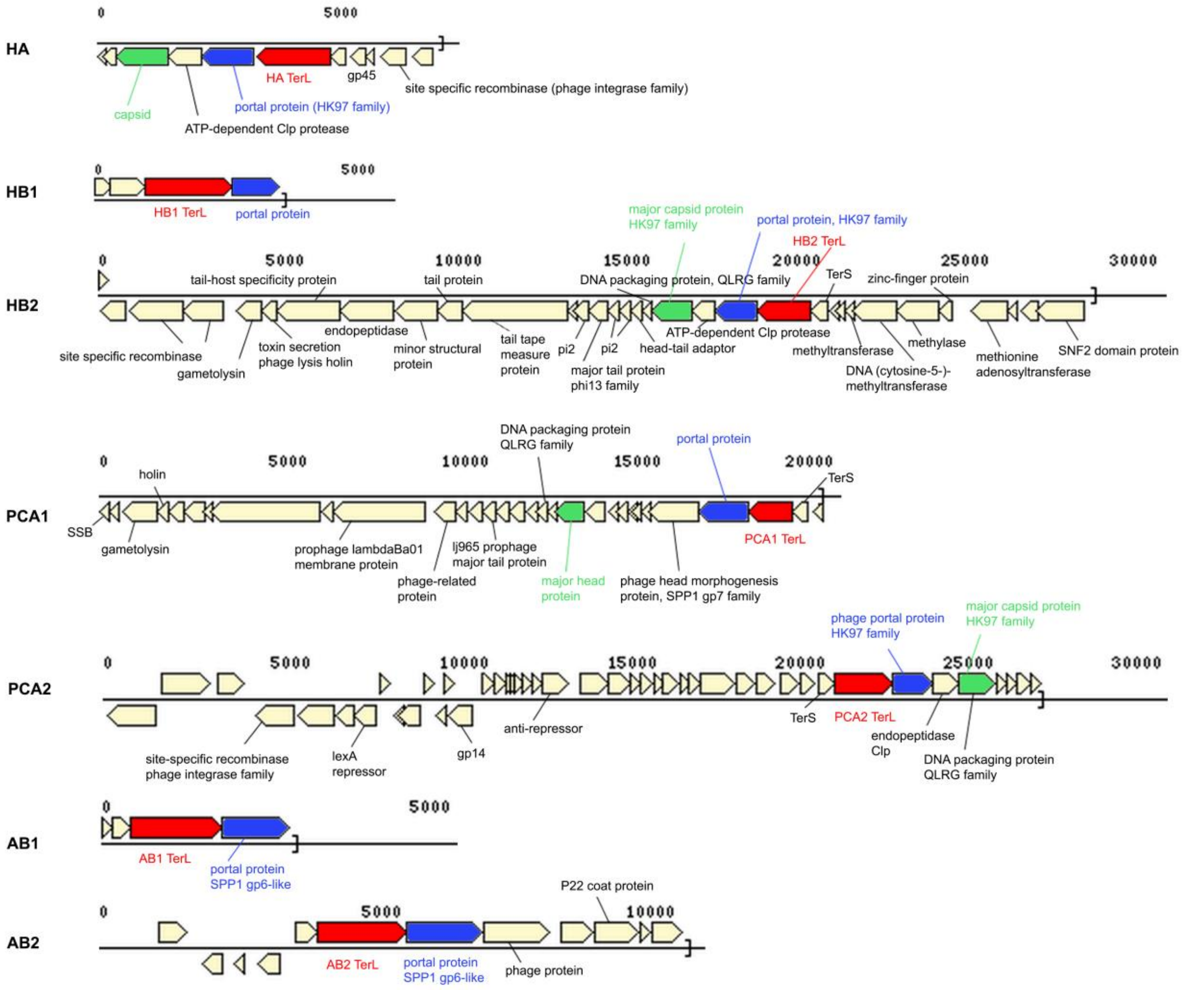

Figure S6. Prophage-like elements harboring close homologs of the TerL markers. For each marker we identified the bacterial isolate harboring the closest homolog in terms of amino acid percent identity, spanning at least 90% of the length of the TerL gene. Prophage-like elements are shown for **a**, HA, **b**, HB2, **c**, PCA1, **d**, PCA2 and **e**, AB2. **f**, HB1. In panels a-e TerL genes in the prophage-like elements aligned with 96% to 99% percent identity at the amino acid level against the markers. In the case of the HB1 marker (panel f), which belongs to the oral ("O") clade of the HB1 TerL lineage (Fig. 3 panel a), the closest homolog was found in *Subdoligranulum sp.* 4_3_54A2FAA, a gut bacterial isolate that grouped within the HB1 gut ("G") clade, and aligned against the HB1 marker with 75% identity at the amino acid level. This result is expected given that both our bioinformatic analysis and experimental data suggest that phages harboring HB1 alleles from the O (oral) clade (including the HB1 marker itself) should be predominately lytic.

The figure shows that close homologs of the TerL markers reside in prophage-like elements spanning at least 34.6 kb for HA, 27.6 kb for HB2, 27.6 kb for PCA1, 26.3 kb for PCA2, 23.7 kb for AB2 and 22.8 kb for HB1. The prophage-like elements shown in this figure were bounded by distinct phage genes as determined based on available annotation. The actual length of the prophage-like elements therefore possibly extends beyond the genomic regions shown. Terminase large and small subunit genes (TerL and TerS, respectively) are highlighted in red, portal protein genes are highlighted in blue, and additional genes associated with virion assembly are highlighted in green.

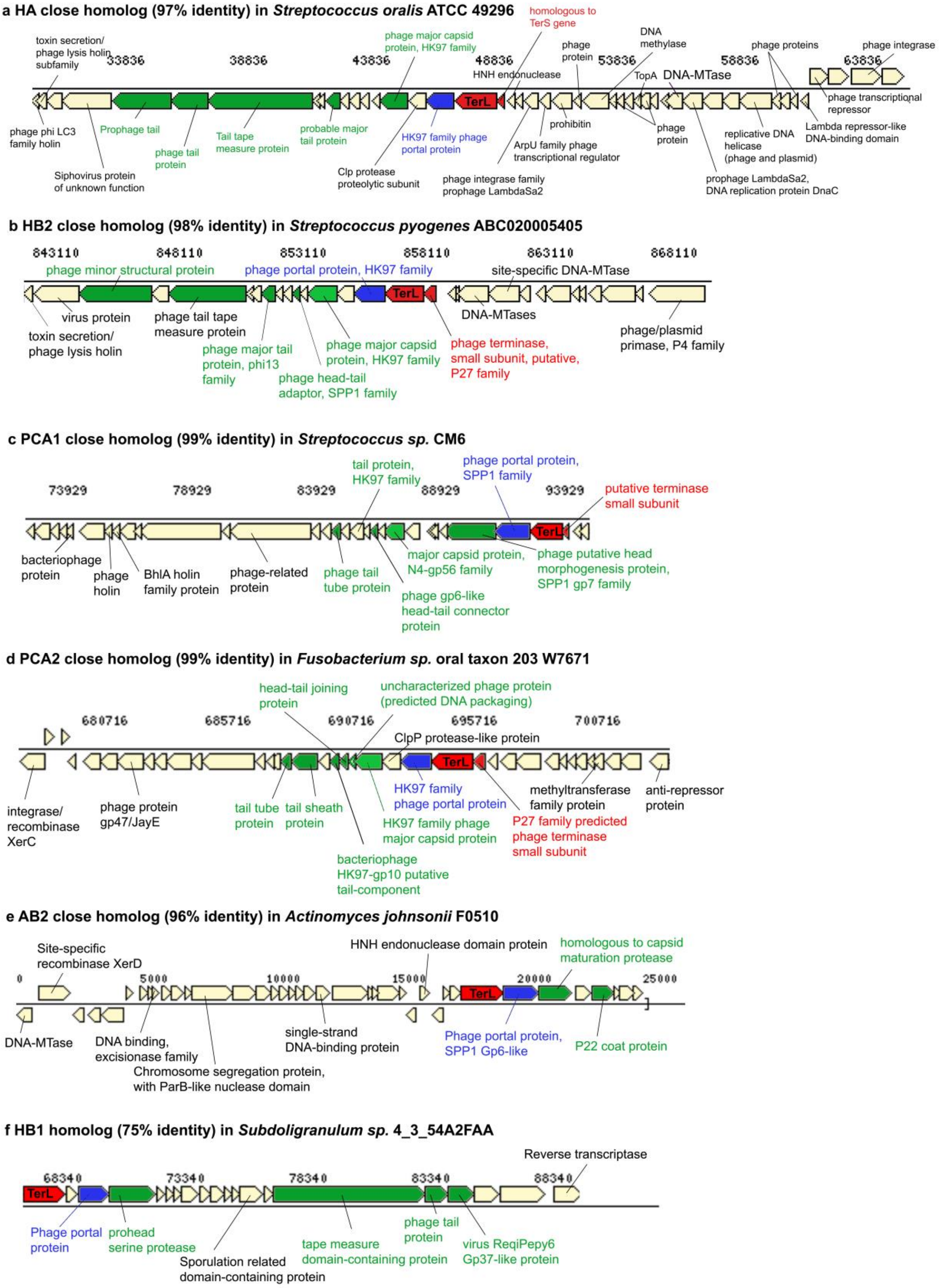
a HA close homolog (97% identity) in Streptococcus oralis ATCC 49296
homologous to TerS gene
toxin secretion/ phage lysis holin subfamily
33836
38836
43836
48836
53836
58836
63836
phage major capsid protein, HK97 family
HNH endonuclease
phage protein
DNA methylase
TopA
DNA-MTase
phage proteins
phage integrase
TerL
phage transcriptional repressor
Lambda repressor-like DNA-binding domain
phage phi LC3 family holin
Prophage tail
phage tail protein
Tail tape measure protein
probable major tail protein
HK97 family phage portal protein
prohibitin
ArpU family phage transcriptional regulator
phage protein
replicative DNA helicase (phage and plasmid)
Siphovirus protein of unknown function
Clp protease proteolytic subunit
phage integrase family prophage LambdaSa2
prophage LambdaSa2, DNA replication protein DnaC
b HB2 close homolog (98% identity) in Streptococcus pyogenes ABC020005405
843110
848110
853110
858110
863110
868110
phage minor structural protein
phage portal protein, HK97 family
site-specific DNA-MTase
TerL
virus protein
phage tail tape measure protein
DNA-MTases
toxin secretion/ phage lysis holin
phage major tail protein, phi13 family
phage major capsid protein, HK97 family
phage terminase, small subunit, putative, P27 family
phage/plasmid primase, P4 family
phage head-tail adaptor, SPP1 family
c PCA1 close homolog (99% identity) in Streptococcus sp. CM6
tail protein, HK97 family
phage portal protein, SPP1 family
73929
78929
83929
88929
93929
putative terminase small subunit
TerL
bacteriophage protein
phage holin
BhlA holin family protein
phage-related protein
phage tail tube protein
major capsid protein, N4-gp56 family
phage putative head morphogenesis protein, SPP1 gp7 family
phage gp6-like head-tail connector protein
d PCA2 close homolog (99% identity) in Fusobacterium sp. oral taxon 203 W7671
head-tail joining protein
uncharacterized phage protein (predicted DNA packaging)
680716
685716
690716
695716
700716
ClpP protease-like protein
TerL
integrase/ recombinase XerC
phage protein gp47/JayE
tail tube protein
tail sheath protein
HK97 family phage portal protein
HK97 family phage major capsid protein
methyltransferase family protein
P27 family predicted phage terminase small subunit
anti-repressor protein
bacteriophage HK97-gp10 putative tail-component
e AB2 close homolog (96% identity) in Actinomyces johnsonii F0510
Site-specific recombinase XerD
HNH endonuclease domain protein
homologous to capsid maturation protease
0
5000
10000
15000
20000
25000
TerL
DNA-MTase
DNA binding, excisionase family
single-strand DNA-binding protein
Phage portal protein, SPP1 Gp6-like
P22 coat protein
Chromosome segregation protein, with ParB-like nuclease domain
f HB1 homolog (75% identity) in Subdoligranulum sp. 4_3_54A2FAA
Reverse transcriptase
68340
73340
78340
83340
88340
TerL
Phage portal protein
prohead serine protease
Sporulation related domain-containing protein
tape measure domain-containing protein
phage tail protein
virus ReqiPepy6 Gp37-like protein

Figure S7. Amplification of the HB1 and HA markers from bacterial and viral fractions of a saliva sample. We attempted to amplify the HB1 and HA TerL markers from bacterial and viral fractions of a saliva sample obtained from an orally healthy subject. Panel a shows results for the HB1 marker and panel b shows results for the HA marker. **Panel a** shows that marker HB1 amplification was absent in the bacterial fraction (3) but present in a subset of viral fractions (5), consistent with a lytic phage at low concentration. 1.) no template control, 2.) extraction control of bacterial fraction, 3.) bacterial fraction, 4.) extraction control of viral fraction, 5.) viral fraction, including replicates, 6.) HB1 marker positive control from previous amplification. Amplified replicates of the same viral extract sample were inconsistently amplified. Replicates of the bacterial fraction did not amplify at lower primer concentrations, nor at higher primer concentrations. Amplification patterns were consistent with an active virus, appearing in the viral fraction at low concentration and low copy numbers. **Panel b** shows that marker HA consistently amplified in bacterial fractions (5) and was absent in viral fractions (7), consistent with a lysogenic phage. 1.) positive control, 2.) total extraction, 3.) no template control, 4.) extraction control of bacterial fraction, 5.) replicates bacterial fraction, 6.) extraction control of viral fraction ,7.) replicates of viral fraction. Replicates of bacterial fraction amplification were successfully amplified in all replicates, and consistently absent in the viral fraction. Amplification patterns were consistent with a prophage, appearing only in the bacterial fraction, rather than the filtered viral fraction.

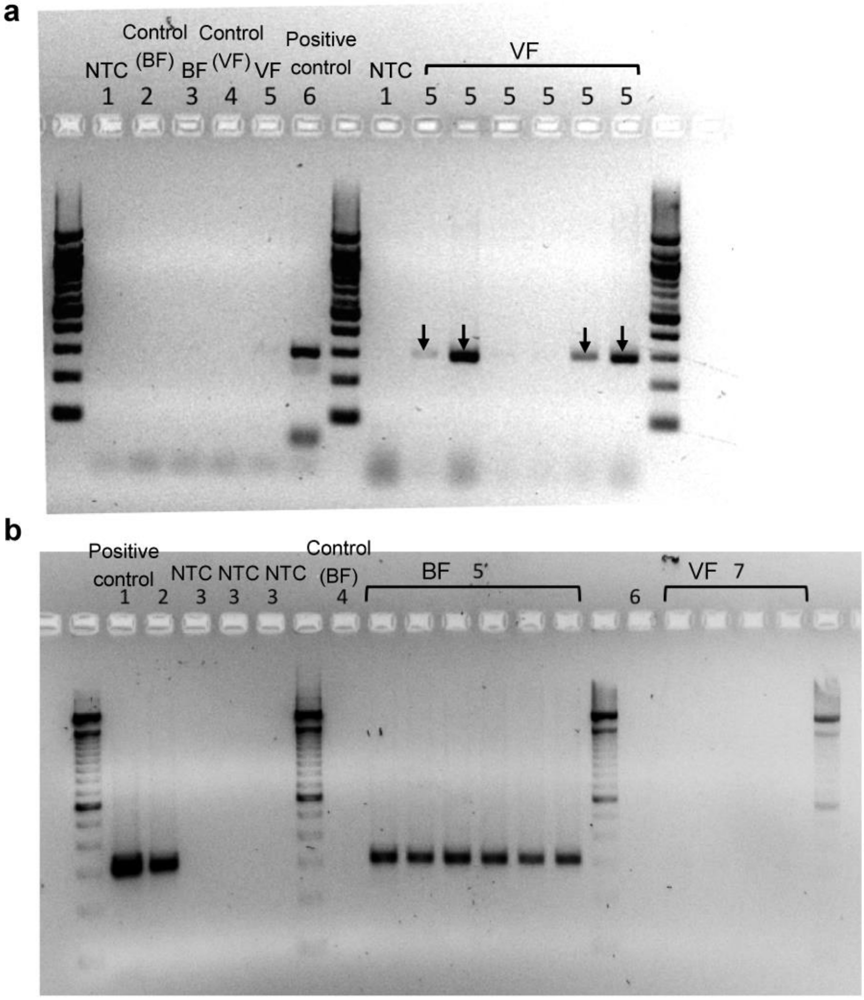

Figure S9. Diversity of HMP metagenomic sequences homologous to the TerL markers as a function of the applied percent identity threshold. The figure below shows for each marker the number of contigs yielding amino acid alignments spanning at least 400 residues and exceeding the given amino acid percent identity threshold, normalized by the number of contigs found when applying a threshold of 40% identity. This analysis shows that TerL diversity increases in a stepwise fashion, with the bulk of sequence diversity forming the 'primary' lineage for each marker, captured using a 70% identity threshold at the amino acid level. We therefore empirically defined a TerL phage family (TerL lineage) associated with a given marker using a 70% identity threshold. Including, however, more distantly related TerL sequences using a lower percent identity threshold did not impact our findings (see Supporting Text S8). Arrows mark ensembles of distant sublineages.

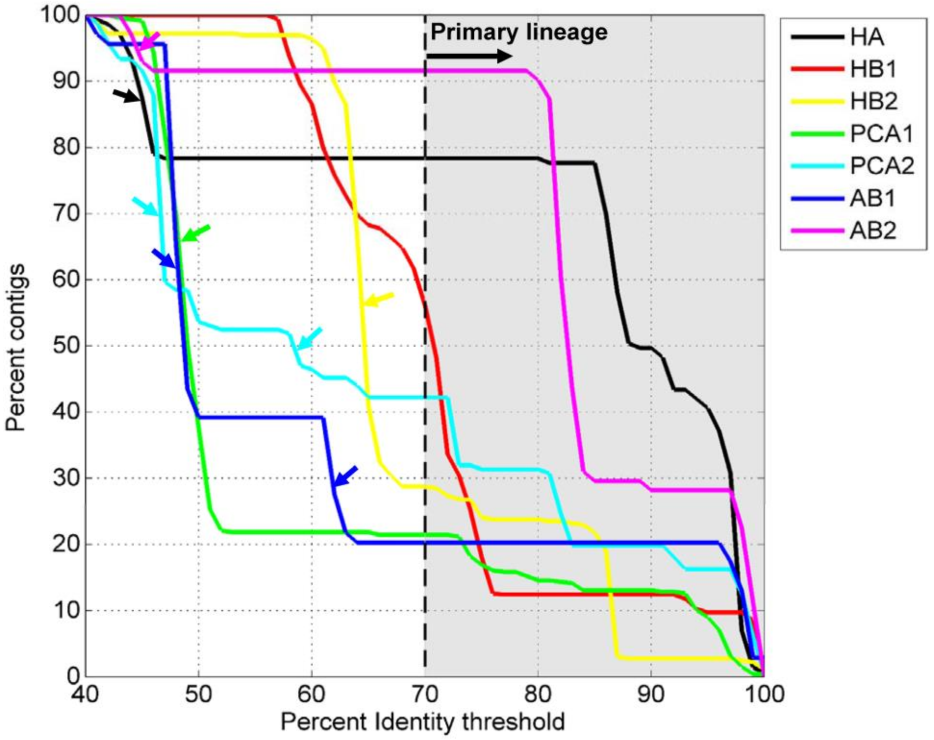

Figure S10. Tree-based phylogenetic analysis of the HB1 TerL lineage. Maximum likelihood tree based on 365 unambiguous amino acid residues of metagenomic alleles and representative human-associated bacterial isolates included in Fig. 3 panel a. Putative recombinant alleles and alleles leading to reticulate network patterns were removed prior to analysis (see below). Phylogenetic analysis was performed as follows: putative recombinant alleles were identified and removed prior to analysis with RDP4 beta 4.28 (Martin and Rybicki, 2000) using the Geneconv (Padidam et al., 1999), Maxchi (Smith, 1992), and RDP (Martin and Rybicki, 2000) algorithms as recommended in the RDP4 manual, and subsequently by identifying remaining alleles leading to reticulate network patterns using a Neighbor-Net analysis. To confirm that all remaining sequences were appropriate for a phylogenetic tree analysis, a likelihood mapping analysis was performed using TREE-PUZZLE 5.0 (Strimmer and Von Haeseler, 1997; Schmidt et al., 2002) with 10000 quartets that showed that 94.2% of the quartets fell in the triangle corners suggesting that a phylogenetic tree should fit the data (Schmidt et al., 2002). The maximum likelihood tree was calculated for 152 amino acid sequences with MEGA (Tamura et al., 2013) using an optimal substitution model predicted by ProtTest3.4 (Darriba et al., 2011) (LG (Le and Gascuel, 2008) +I +G) with 1000 bootstrap iterations. A discrete Gamma distribution was used to model evolutionary rate differences among sites (five categories (+G, parameter 0.8612)). The rate variation model allowed for some sites to be evolutionarily invariable ([+I], 19.02% sites). Branch lengths are measured in the number of substitutions per site. Support values greater than 50% for 1000 bootstrap iterations are shown.

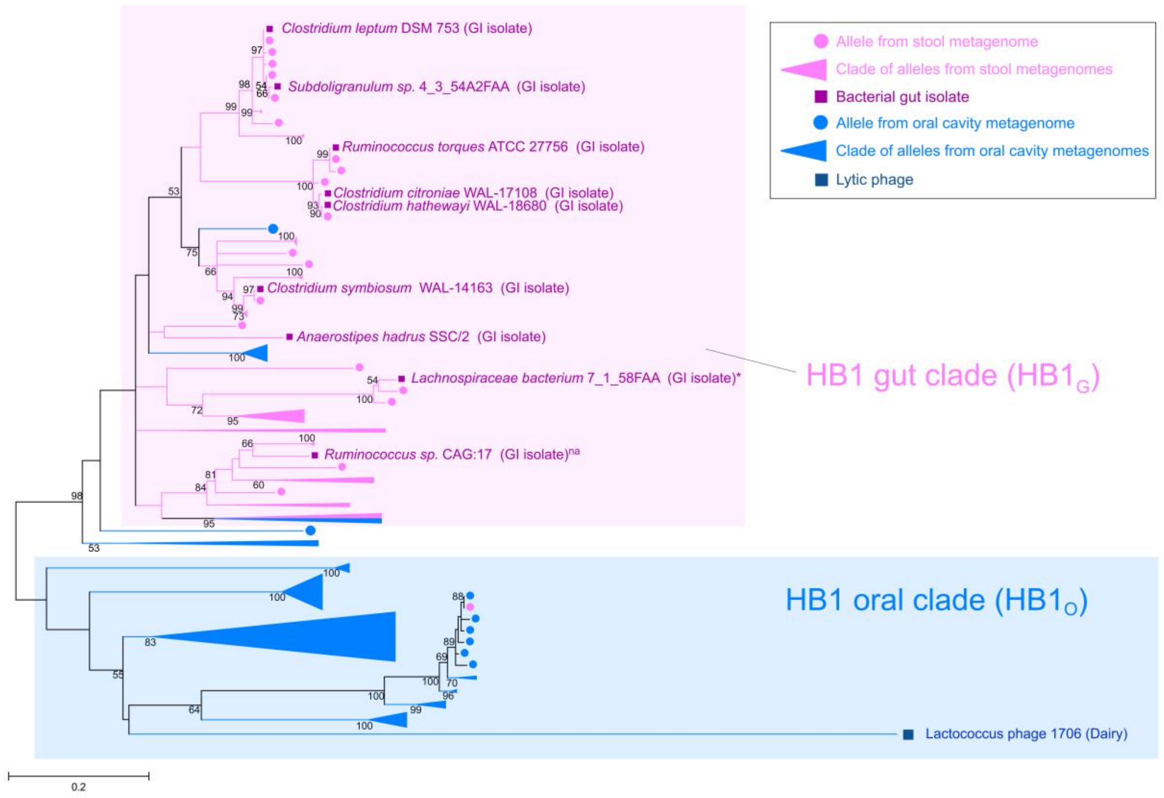

Figure S11. Phylogenetic analysis of HB1 and HA TerL lineages. Neighbor-Net analysis drawn with SplitsTree4 (Huson and Bryant, 2006) for **a,** 386 unambiguous amino acid residues of the HB1 TerL marker, and **b,** 351 unambiguous amino acid residues of the HA TerL marker based on alleles obtained from the HMP metagenomes (circular nodes) and sequenced bacterial and phage isolates (square nodes). S. = *Streptococcus*. For additional details see caption of Fig. 3. Phylogenetic analysis of HB1 was based on 333 sequences using the optimal WAG+I+G model based on the AIC criterion with optimal α and Pinv parameters. Phylogenetic analysis of HA was based on 112 sequences using the optimal JTT+I+G model based on the AIC criterion with optimal α and Pinv parameters.

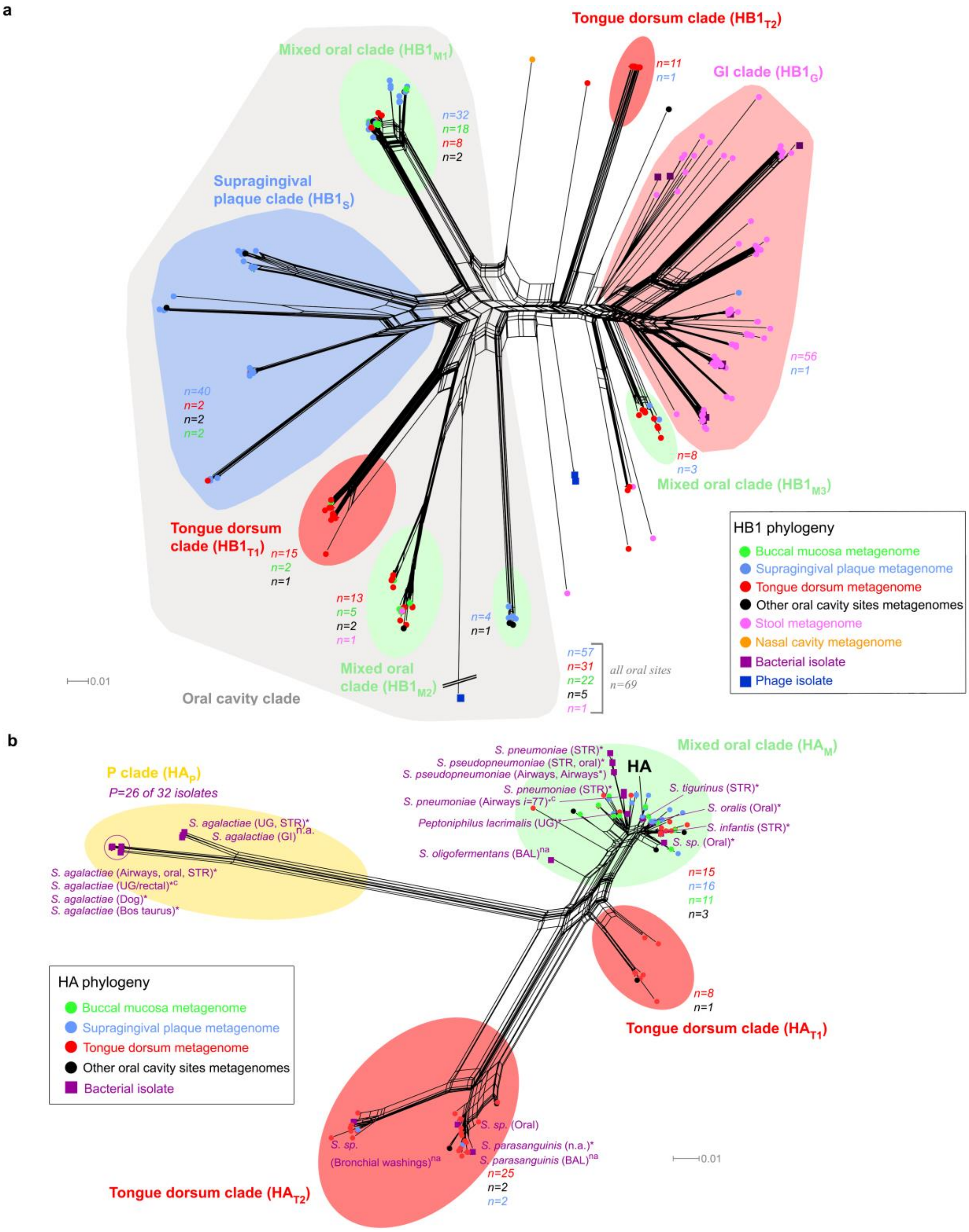

a
Tongue dorsum clade (HB1T2)
Mixed oral clade (HB1M1)
GI clade (HB1G)
Supragingival plaque clade (HB1S)
Tongue dorsum clade (HB1T1)
Mixed oral clade (HB1M2)
Mixed oral clade (HB1M3)
Oral cavity clade
all oral sites n=69
HB1 phylogeny
Buccal mucosa metagenome
Supragingival plaque metagenome
Tongue dorsum metagenome
Other oral cavity sites metagenomes
Stool metagenome
Nasal cavity metagenome
Bacterial isolate
Phage isolate
0.01
b
P clade (HAP)
P=26 of 32 isolates
Mixed oral clade (HAM)
HA
Tongue dorsum clade (HAT1)
Tongue dorsum clade (HAT2)
S. pneumoniae (STR)*
S. pseudopneumoniae (STR, oral)*
S. pseudopneumoniae (Airways, Airways*)
S. pneumoniae (STR)*
S. pneumoniae (Airways i=77)*c
Peptoniphilus lacrimalis (UG)*
S. oligofermentans (BAL)na
S. tigurinus (STR)*
S. oralis (Oral)*
S. infantis (STR)*
S. sp. (Oral)*
S. agalactiae (UG, STR)*
S. agalactiae (GI)n.a.
S. agalactiae (Airways, oral, STR)*
S. agalactiae (UG/rectal)*c
S. agalactiae (Dog)*
S. agalactiae (Bos taurus)*
S. sp. (Oral)
S. parasanguinis (n.a.)*
S. parasanguinis (BAL)na
S. sp. (Bronchial washings)na
HA phylogeny
Buccal mucosa metagenome
Supragingival plaque metagenome
Tongue dorsum metagenome
Other oral cavity sites metagenomes
Bacterial isolate
0.01

Figure S12. Phylogenetic analysis of PCA1 and PCA2 TerL lineages. Neighbor-Net analysis drawn with SplitsTree4 (Huson and Bryant, 2006) for **a,** 381 unambiguous amino acid residues of the PCA1 TerL marker, and **b,** 386 unambiguous amino acid residues of the PCA2 TerL marker based on alleles obtained from the HMP metagenomes (circular nodes) and sequenced bacterial and phage isolates (square nodes). *S.* = *Streptococcus*. For additional details see caption of Fig. 3. Phylogenetic analysis of PCA1 was based on 122 sequences using the optimal CpREV+I+G model based on the AIC criterion with optimal α and Pinv parameters. Phylogenetic analysis of PCA2 was based on 63 sequences using the optimal WAG+I+G model based on the AIC criterion with optimal α and Pinv parameters.

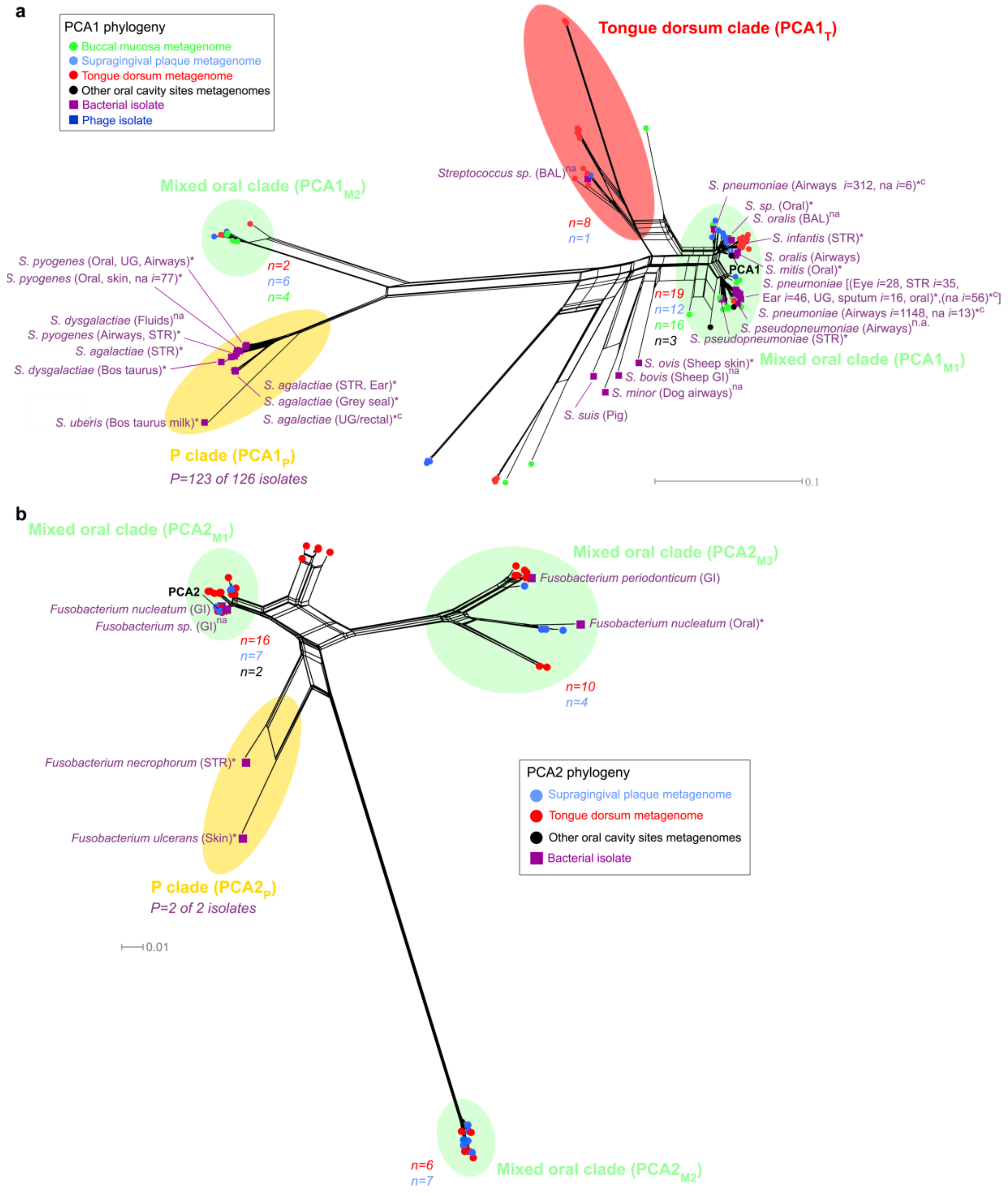

Figure S13. Phylogenetic analysis of TerL lineages recovered from in individual subjects. Each panel shows a Neighbor-Net analysis drawn with SplitsTree4 (Huson and Bryant, 2006) of all close homologs of a given marker obtained from all habitats associated with a particular subject in both visit 1 and visit 2. Networks for subject MRN-763961826 are shown for **a**, HB1, **b**, PCA1 and **c**, HA. Networks for HB1 are also shown for: **d**, subject MRN-159268001, and **e**, subject MRN-7763577454 (the MRN - Medical Record Number - is a unique subject identifier used in the HMP study). All networks are calculated for amino acid sequences. Nodes and labels are color coded by habitat as indicated in the legend. Alleles from visit number 1 are highlighted in yellow, and alleles from visit no. 2 are highlighted in blue. Nodes are labeled according to the convention $pPsSvV(b)aA$, where P is a unique subject identifier corresponding to the MRN of the subject ($1 \le P \le 103$ subjects); S is an integer index corresponding to the habitat as follows:1. attached/keratinized gingiva, 2. buccal mucosa, 3. hard palate, 4. palatine tonsils, 5. saliva, 6. subgingival plaque, 7. supragingival plaque, 8. throat, 9. tongue dorsum, 10. stool, 11. anterior nares, 12. posterior fornix, 13. mid vagina, 14. vaginal introitus, and the retroauricular crease (15. unspecified, 16. left crease, 17. right crease); V is the visit number ($V = 1$,2, or 3) with the optional index b representing a replicate of the sample; A is an integer index corresponding to the TerL allele in the metagenome.

Although coverage bias may play a role in marker variably, sequence data appears to support a hypothesis of host migration between oral habitats. For instance, panel a shows an example of an HB1 allele belonging to an "M" clade (M1 clade) that was found to be spread out simultaneously in the same subject across five oral habitats, including the buccal mucosa. In another example, panel b shows a possible host migration event involving a PCA1 allele, also belonging to an "M" clade, from gums to the buccal mucosa occurring between two consecutive visits. In this example, a PCA1 allele associated with the M1 clade (by virtue of sequence similarity) is shown to be present in the gums and absent in the buccal mucosa in visit 1 (the closest homolog of the PCA1 marker in the buccal mucosa metagenome yielded only 36% identity at the amino acid level). However, in visit 2 this allele was detected in the buccal mucosa, despite the buccal mucosa metagenome from visit 2 having a lower coverage compared to the buccal mucosa metagenome in visit 1 (309 vs. 437 nt/gene). This example supports the hypothesis that TerL alleles in "M"-type clades may be associated bacterial hosts that potentially migrate from one oral habitat to another.

Phylogenetic analysis of HB1 in panels a, d and e was based on 314, 398 and 398 unambiguous amino acid residues obtained from 14, 16 and 19 sequences using the optimal WAG+I, WAG+I+G and the WAG+I+G model based on the AIC criterion with an optimal α (d, e) parameter and an optimal Pinv (a, d, e) parameter, respectively. Phylogenetic analysis of PCA1 in panel b was based on 291 unambiguous amino acid residues obtained from 8 sequences using the optimal WAG+I model based on the AIC criterion with an optimal Pinv parameter. Phylogenetic analysis of HA in panel c was based on 314 unambiguous amino acid residues obtained from 8 sequences using the optimal WAG+I model based on the AIC criterion with an optimal Pinv parameter.

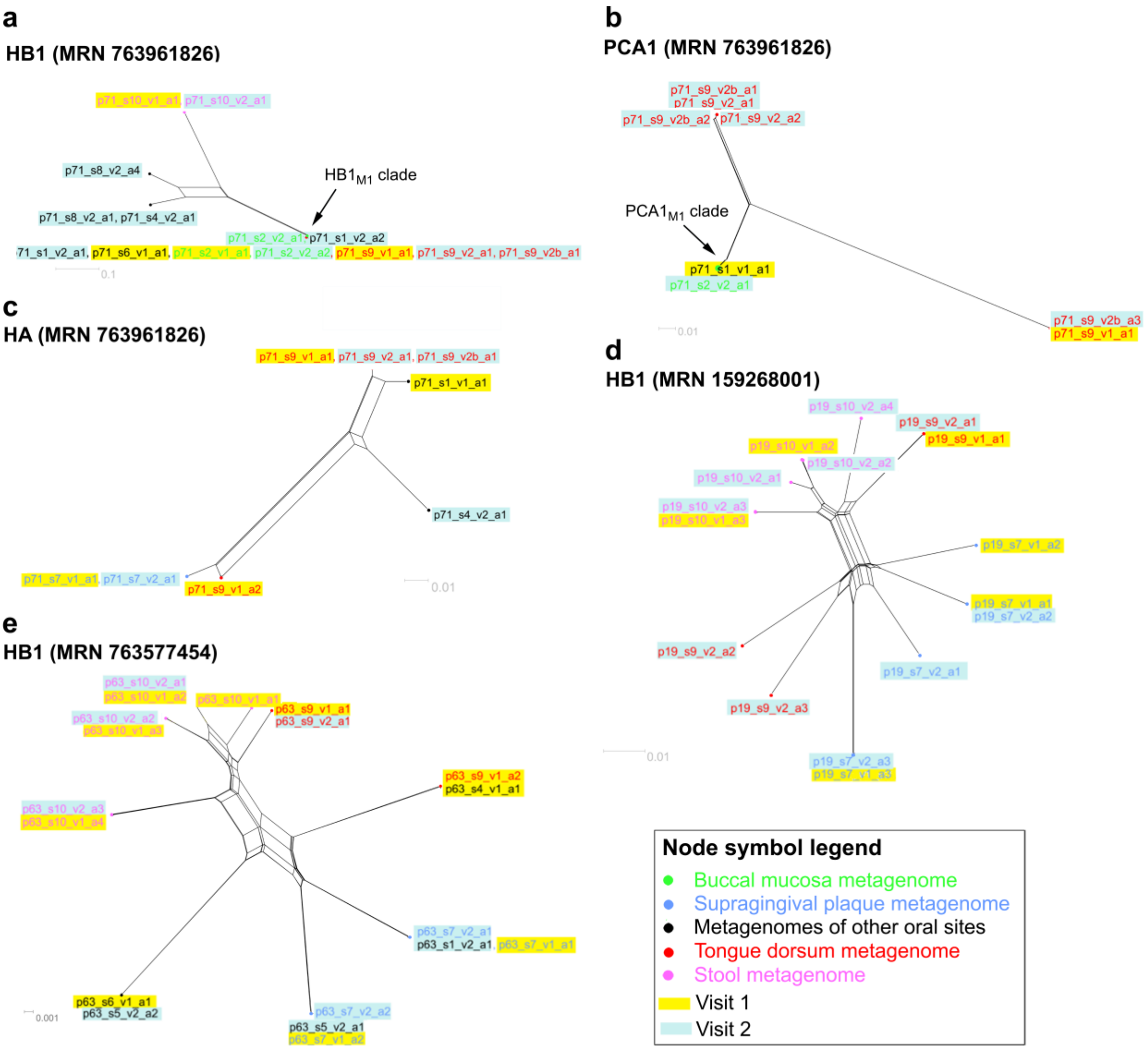
a
HB1 (MRN 763961826)
p71_s10_v1_a1, p71_s10_v2_a1
p71_s8_v2_a4
p71_s8_v2_a1, p71_s4_v2_a1
HB1M1 clade
p71_s2_v2_a1, p71_s1_v2_a2
71_s1_v2_a1, p71_s6_v1_a1, p71_s2_v1_a1, p71_s2_v2_a2, p71_s9_v1_a1, p71_s9_v2_a1, p71_s9_v2b_a1
0.1
b
PCA1 (MRN 763961826)
p71_s9_v2b_a1
p71_s9_v2_a1
p71_s9_v2b_a2
p71_s9_v2_a2
PCA1M1 clade
p71_s1_v1_a1
p71_s2_v2_a1
0.01
p71_s9_v2b_a3
p71_s9_v1_a1
c
HA (MRN 763961826)
p71_s9_v1_a1, p71_s9_v2_a1, p71_s9_v2b_a1
p71_s1_v1_a1
p71_s4_v2_a1
p71_s7_v1_a1, p71_s7_v2_a1
p71_s9_v1_a2
0.01
d
HB1 (MRN 159268001)
p19_s10_v2_a4
p19_s9_v2_a1
p19_s9_v1_a1
p19_s10_v1_a2
p19_s10_v2_a2
p19_s10_v2_a1
p19_s10_v2_a3
p19_s10_v1_a3
p19_s7_v1_a2
p19_s7_v1_a1
p19_s7_v2_a2
p19_s9_v2_a2
p19_s7_v2_a1
p19_s9_v2_a3
0.01
p19_s7_v2_a3
p19_s7_v1_a3
e
HB1 (MRN 763577454)
p63_s10_v2_a1
p63_s10_v1_a2
p63_s10_v1_a1
p63_s9_v1_a1
p63_s9_v2_a1
p63_s10_v2_a2
p63_s10_v1_a3
p63_s9_v1_a2
p63_s4_v1_a1
p63_s10_v2_a3
p63_s10_v1_a4
p63_s7_v2_a1
p63_s1_v2_a1, p63_s7_v1_a1
p63_s6_v1_a1
p63_s5_v2_a2
0.001
p63_s7_v2_a2
p63_s5_v2_a1
p63_s7_v1_a2
Node symbol legend
Buccal mucosa metagenome
Supragingival plaque metagenome
Metagenomes of other oral sites
Tongue dorsum metagenome
Stool metagenome
Visit 1
Visit 2

SUPPORTING TABLES

Table S1. Nonredundant list of viral RefSeq genes reported by MCRL for the metagenomes in the Mira dataset (provided as an excel file). This table provides the raw output of the MCRL algorithm (Tadmor and Phillips, 2022) when applied to each of the six oral metagenomes from the Mira study. For each oral metagenome the table lists the viral RefSeq genes with significant BLAST hits in the given metagenome (E value $<10^{-7}$ at the amino acid level) that were determined by MCRL to have non-overlapping signatures in the metagenome using a stringent overlap condition. Columns are as follows: "Index": counter of viral RefSeq gene in the table, "Reference gene": name of viral RefSeq gene as it appears in the definition line of the viral RefSeq database, "Representative contig": identifier of contig that yielded the minimal E value when BLASTing the amino acid sequence of the given viral RefSeq gene against the translated metagenome, "Size of signature of reference gene": number of contigs in the signature of the given viral RefSeq gene, "Size of signature of reference gene cluster": number of contigs in the union of all signatures of all viral RefSeq genes in the given 'reference gene cluster'. The 'reference gene cluster' is the group of all viral RefSeq genes that have overlapping signatures and 'elected' the given viral RefSeq gene (Tadmor and Phillips, 2022), "# of related reference genes": number of viral RefSeq genes in the given 'reference gene cluster'. The next seven columns (columns G – M) correspond to details regarding the amino acid alignment between the given viral RefSeq gene and the representative contig, "Representative contig aa sequence": the amino acid sequence of the representative contig, "Representative contig definition": description of representative contig as provided in the metagenome FASTA file, "Reference gene aa sequence": the amino acid sequence of the given viral RefSeq gene, the following seven columns (columns Q – W) are various definitions of the viral RefSeq gene parsed from the FASTA and GenPept files of the viral RefSeq database.

Table S2. Nonredundant list of viral RefSeq genes reported by MCRL for the metagenomes in the Mira dataset encoding a TerL gene (provided as an excel file). This table lists the subset of viral RefSeq genes from Table S1 that encode a TerL gene with a signature size of 5 or higher and that share at least 10% amino acid residues when aligned against the representative contig. Representative contigs yielding at least 75% identity at the amino acid level when BLASTed against the Xie metagenome are highlighted in yellow (all alignments were performed at the amino acid level). In total 11 TerL candidates were identified in this manner, which grouped by homology in the following way: {(HB2), (HA), (AB1), (AB2), (PCA2), (HB1, PCB1, AA1), (PCA1, PCB2, AA2)}. TerL genes in this table are sorted by signature size. Columns D – X are the same as the corresponding columns in Table S1. The last three columns show the amino acid alignment information of the top hit when BLASTing the amino acid sequence of the representative contig of the given RefSeq TerL gene against the dataset indicated in the column. Alignment information includes: E value, number of identical amino acids, total number of amino acids in the local alignment, and the percent of identical amino acids.

Table S3. Definition of TerL markers and corresponding degenerate primers (provided as an excel file). Definition of the full-length TerL markers and degenerate primers targeting the TerL lineages. Columns are as follows: "TerL marker": name of full-length TerL marker, "IMG genome ID": IMG genome ID identifying the metagenome from which the full-length TerL marker was obtained (https://img.jgi.doe.gov), "IMG gene ID": IMG gene ID identifying the HMP contig encoding the full-length TerL marker, "aa sequence": amino acid sequence of the full-length TerL marker, "Length (aa)": length of full-length TerL marker in amino acids, "pfam/COG": corresponding pfam and COG associated with the TerL marker, "E value": E value of BLAST alignment against the indicated pfam/COG in CDD (Marchler-Bauer et al., 2016) v3.16, "Phages in pfam/COG": phages with published functional signatures that belong to the given pfam or COG, "Degenerate primers": Forward and reverse degenerate primers targeting the given TerL marker, "Forward/Reverse": F=forward primer; R=reverse primer, "Conserved aa motif": conserved amino acid motifs targeted by the indicated forward or reverse primer. Residues highlighted in red were invariant in the CDD alignment, and residues highlighted in blue received an RPS-BLAST score greater or equal to 3.5 bits, "# of alleles used for primer design": number of TerL alleles in the amino acid sequence alignment used to design the given degenerate primer, "Amplicon size (bp)": expected PCR amplicon length for given primer set.

Table S4. Pairwise alignment of full-length TerL markers. All pairwise alignment combinations of full-length TerL markers. For each combination of markers, the following table shows the percent identity and the alignment length obtained for the top BLAST solution. Alignments were performed on amino acid sequences. Results corresponding to alignment lengths shorter than 50 amino acids are not shown.

	HA	HB1	HB2	PCA1	PCA2	AB1	AB2
HA							
HB1	21.1% (468)						
HB2	21.3% (352)	22.6% (540)					
PCA1	-	-	-				
PCA2	25.3% (367)	20.2% (574)	27.3% (506)	-			
AB1	-	-	22.9% (244)	-	-		
AB2	-	-	30.3% (66)	-	-	-	

Table S5. Diversity of TerL lineages based on HMP metagenomes and PCR-amplified sequences. The following table compares the average percent identity between TerL markers and human-associated TerL alleles (metagenomic and PCR-amplified) versus the closest hit of the TerL markers in NCBI's env_nr database. The table shows that human-associated TerL alleles were closely related to the TerL markers, with homologs in a significant number of subjects yielding at least 95% identity at the amino acid level. In contrast, most TerL markers had only remote homologs in env_nr. All alignments were performed on amino acid sequences, with alignments of markers against human-associated TerL alleles performed using BLAST and alignments of markers against the env_nr database performed using PSI-BLAST.

Columns are as follows: "alignment length (aa)": number of unambiguous amino acid residues in the given alignment, "*n* sequences": number of TerL sequences in the amino acid alignment. In the case of the HMP cohort, identical sequences from the same subject and sampling site were removed, selecting alleles spanning at least 400 amino acids (200 amino acids for AB1) and yielding at least 70% identity at the amino acid level when locally aligned against the markers. "*n* subjects": total number of subjects from which homologues alleles were obtained, "Average % identify": average percent identity (±s.d.) at the amino acid level obtained when aligning TerL sequences against the indicated TerL marker, "Maximum % identify": maximum percent identity at the amino acid level between aligned TerL sequences and the indicated TerL marker, No. subjects yielding >95% identity": number of subjects for which the indicated marker aligned with >95% identity at the amino acid level compared to metagenomic sequences, "% identity of top hit in env_nr": percent identity of hit yielding the lowest E value when aligning the TerL markers against NCBI's env_nr database using PSI-BLAST. The lower table is analogous to upper table showing alignments corresponding to translated PCR-amplified sequences obtained using targeted sequencing.

	HMP cohort					
**TerL marker**	**alignment length (aa)**	***n* sequeneces**	***n* subjects**	**Average % identity**	**No. subjects yielding >95% identity**	**% identity of top hit in env_nr**
**HB1**	369	318	85	79±10.3	38	26
**HB2**	340	82	53	85±6.8	7	52
**PCA2**	385	56	39	88.3±10.6	22	31
**PCA1**	384	86	54	88.8±9.9	33	35
**HA**	351	87	53	93.7±5.1	36	26
**AB2**	350	51	40	87±7.9	15	34
**AB1**	390	9	5	98.3±0.7	5	26

	PCR-amplified			
**TerL marker**	**alignment length (aa)**	***n* sequeneces**	**Average % identity**	**Maximum % identity**
**HB1**	80	124	87.8±16	100
**HB2**	105	8	85.1±0.4	85.7
**PCA2**	59	106	93.1±12.5	100
**PCA1**	93	10	96.4±1.1	97.8
**HA**	81	169	98.1±3	100
**AB2**	139	14	81.4±8.2	96.4
**AB1**	n/a	n/a	n/a	n/a

Table S6. Selection pressure analysis of TerL lineages (provided as an excel file). Selection pressure analysis was performed on PCR-amplified sequences and close homologs of the TerL markers obtained from HMP metagenomes. The selection pressure analysis was performed on nucleotide alignments spanning at least two thirds of the TerL gene length using the PAML package (Yang, 2007) assuming one site class (model M0, fixed ω), two site classes (model M1a, $\omega = 1$ and $\omega < 1$), and three site classes (model M2a, $\omega = 1$, $\omega < 1$ and $\omega > 1$). For each model, associated log likelihoods and estimated ω values are reported, where ω represents the ratio of the rate of non-synonymous substitutions to the rate of synonymous substitutions. To enable a comparison between different models, the likelihood ratio test statistic is denoted with its statistical significance determined based on the chi-squared distribution. We found that for all markers, M1a was preferred over M0 (P value < 0.001), and adding positive selection pressure (M2a) did not result in a better model. The M1a models show that for all markers the vast majority of sites (89 to 99% of sites) were under substantial negative selection pressure ($0.01 < \omega < 0.07$). Columns are as follows: "TerL marker": the TerL marker for which selection pressure was assessed based on either metagenomic sequences (upper part of table) or PCR-amplified sequences (lower part of table), "No. of sequences": number of sequences used in the analysis, "Alignment length (nts)": number of nucleotides in the alignment. For each model (M0, M1a, and M2a) the log likelihood score for the given model and the estimated value for ω are provided, with the fraction of sites estimated to have the specified site class shown in parenthesis. The last four columns show the chi-squared values comparing M0 to M1a and M1a to M2a followed by the decision whether to reject M0 in favor of M1a, and reject M1a in favor M2a, respectively, based on the statistical significance determined according to the given chi-squared distribution.

				One site class model Model M0 (fixed ω)		Two site classes model Model M1a (ω=1 and ω<1)				Three site classes model Model M2a (ω=1, ω<1, and ω>1)					M0 vs M1a		M1a vs M2a	
	TerL marker	No. of sequences	Alignment length (nts)	Log Likelihood (Ln0)	Estimated ω	Log Likelihood (Ln1)	Estimate d ω	ω=1	ω<1	Log Likelihood (Ln2)	Estimated ω	ω=1	ω<1	ω>1	Chi^2 = 2 (Ln1 - Ln0)	**Reject Model M0 in favor of M1a? (d.f. =1)**	Chi^2= 2 (Ln2 - Ln1)	**Reject Model M1a in favor of M2a? (d.f.=2)**
**Metagenomically-derived marker sequences**	**HB1**	30	1158	-18990.3	0.023	-18976.1	0.048	1 (2.1%)	0.02831 (97.9%)	-18976.1	0.048	1 (2.1%)	0.02831 (97.9%)	34.65107 (0%)	28.5	yes, P <0.001	0	no, P > 0.1
	**HA**	88	1053	-14936.8	0.024	-14855.6	0.038	1 (1.9%)	0.01946 (98.1%)	-14855.6	0.038	1 (1.9%)	0.01946 (98.1%)	43.77607 (0%)	162.3	yes, P <0.001	0	no, P > 0.1
	**PCA2**	57	1158	-10238.7	0.021	-10199.9	0.029	1 (1.1%)	0.01871 (98.9%)	-10199.9	0.029	1 (1.1%)	0.01871 (98.9%)	26.09248 (0%)	77.5	yes, P <0.001	-1E-05	no, P > 0.1
	**PCA1**	87	1140	-24101.6	0.031	-23936.3	0.041	1 (1.6%)	0.02544 (98.4%)	-23936.3	0.041	1 (0.1%)	0.02544 (98.4%)	1 (1.5%)	330.7	yes, P <0.001	0	no, P > 0.1
	**HB2**	83	1023	-12309.3	0.041	-12241.2	0.063	1 (3.0%)	0.03332 (97.0%)	-12241.2	0.063	1 (3.0%)	0.03332 (97.0%)	26.59471 (0%)	136.3	yes, P <0.001	0	no, P > 0.1
	**AB2**	26	1308	-8152.8	0.021	-8062.6	0.047	1 (3.4%)	0.01388 (96.6%)	-8062.6	0.047	1 (3.4%)	0.01388 (96.6%)	45.75934 (0%)	180.4	yes, P <0.001	0	no, P > 0.1
	**AB1**	14	1017	-2743.3	0.015	-2711.1	0.021	1 (1.3%)	0.01341 (98.7%)	-2710.2	0.032	1 (0.8%)	0.00817 (98.7%)	3.46838 (0.5%)	64.3	yes, P <0.001	1.84829	no, P > 0.1
**Primer-derived**	**HB1**	123	243	-7560.4	0.036	-7456.8	0.152	1 (11.1%)	0.046 (88.9%)	-7456.8	0.152	1 (11.0%)	0.046 (88.9%)	1 (0.1%)	207.2	yes, P <0.001	0	no, P > 0.1
	**HA**	168	246	-4004.5	0.031	-3987.9	0.049	1 (2.5%)	0.025 (97.5%)	-3987.9	0.049	1 (2.5%)	0.025 (97.5%)	3.7 (0%)	33.3	yes, P <0.001	3E-05	no, P > 0.1
	**PCA2**	105	180	-2003.3	0.073	-1993.9	0.139	1 (8.1%)	0.063(91.9%)	-1993.9	0.139	1 (3.1%)	0.063 (91.9%)	1 (5.0%)	18.7	yes, P <0.001	-2.8E-05	no, P > 0.1

Table S7. Conserved functional signatures in TerL lineages (provided as an excel file)**.** Summary of functional signatures of the TerL gene identified in PCR-amplified sequences and in metagenomic alleles based on the amino acid alignments shown in Fig. S3. Functional signatures are shown for the following TerL lineages: **a,** HB2, **b,** PCA2, **c,** HA, **d,** HB1, and **e,** PCA1. For each functional signature we note the number of HMP and PCR-amplified sequences in the alignments and the total number of subjects from which sequences were obtained. Signatures are color coded as follows: a position in an alignment encoding one or more amino acids that match the expected residue(s) for the given TerL functional signature is highlighted in **dark green**. A position in an alignment encoding one or more amino acids that were substituted compared to the expected residue(s) within the given TerL functional signature, scoring positive, negative or neutral BLOSUM62 scores are highlighted in **light green**, **red** and **gray**, respectively. Expected residues are based either on published functional signatures of the TerL gene (Rao and Feiss, 2008; Sun et al., 2008), or on residues found in homologous TerL sequences that belong to phages that have 3D-stucture based domain models of their TerL genes in CDD (Marchler-Bauer et al., 2016) and that are known to be functional phages (HK97, Lactococcus virus c2, SPP1, T5). For one marker (PCA1) we included Lj928 - a noninducible but complete prophage sequence in *Lactobacillus johnsonii* NCC 533 that was used to explain one of the conserved residues in the ATPase coupling motif of PCA1. Z denotes a hydrophobic residue (G, A, V, L, I, P, F, M, W) and X denotes any residue.

Table S8. HMP metagenomes that passed HMP quality control criteria (provided as an excel file). The tab "after QC" provides the IMG genome identifiers as well as metadata and data statistics fields exported from the IMG platform (https://img.jgi.doe.gov/) for the 690 HMP metagenomes that passed HMP quality control criteria and that were analyzed in this study. The tab "before QC" provides the IMG genome identifiers as well as additional metadata fields exported from the IMG platform for all 748 metagenomes generated in Phase I of the HMP study prior to filtering metagenomes based on HMP quality control criteria.

Table S9. Environmental metagenomes and viromes interrogated for the presence of TerL markers (provided as an excel file)**. Panel a, IMG/M environmental metagenomes.** Columns are as follows: "Class", "Order" and "Family": IMG designation of environment corresponding to 'class', 'order' and 'family', respectively, "Taxon index": index of environment corresponding to Fig. 1 panel g, "IMG genome ID": IMG genome ID of environment, "Genome Name": IMG description of the given environment. Additional columns include genome, metadata and data statistics fields exported from the IMG platform (https://img.jgi.doe.gov/). At the end of the table are statistics related to representative ribosomal proteins (pfam00318, pfam00347, pfam00411, pfam00573, pfam01196), including the mean and median read depth, and total number of genes detected in the given metagenome. **Panel b, environmental viromes.** All columns were exported from the MetagenomesOnline (MgOl) portal for virome datasets (http://metagenomesonline.org/home) hosted on the VIROME platform (36) (http://virome.dbi.udel.edu/). Selection criteria for metagenomes and viromes are provided in the Material and Methods section. **Panel c IMG/VR environmental metagenomes**, Metadata for all environmental metagenomes included in Fig. S8.

Table S10. Bacterial and phage isolates harboring close homologs of the TerL markers (provided as an excel file). Sequenced bacterial and phage genomes harboring close homologs of the markers yielding at least 70% identity at the amino acid level across at least 90% of the length of the marker. The table lists all bacterial and phage isolates found in (i) the IMG database, (ii) NCBI's non-redundant (nr) protein database, and (iii) HOMD. Isolates belonging to "P" clades in phylogenetic networks are highlighted in yellow. Alphabetical references (shown in green) are provided at the bottom of each table. Columns are as follows: "Origin": origin of isolate, e.g.: human, animal, or environment, "Database": database in which the isolate was found, "Percent Identity": Percent identity achieved by BLAST alignment of the marker sequence against the TerL gene in the isolate genome, performed on amino acid sequences, "NCBI Taxon ID": NCBI taxon ID of isolate, "IMG taxon ID": IMG taxon ID of isolate, "Health-related status": health-related status of the bacterial or phage isolate as defined in the Materials and Methods section. If no reference is provided in this field, information was derived from IMG annotation, "Annotation regarding health-related status": description of supporting evidence for the designation of the health-related status. Unless otherwise stated, the description provided in this column was based on IMG annotation. "Reference": references supporting the designation of the health-related status. "Additional markers in genome": if the bacterial isolate contained additional close homologs of TerL markers those markers are specified, "Phylogenetic analysis": "X" indicates that the TerL sequence corresponding to the given isolate was used for phylogenetic analysis. Other strains belonging to the same OTU are indicated by percent identity. See Materials and Methods for details how OTU assignment was performed. "P" clade: isolate belongs to a "P" clade in the corresponding phylogenetic network.

In the case of PCA2, we found close homologs of PCA2 in a gastric bacterial isolate and in a colon bacterial isolate from the *Fusobacterium* genus, despite finding no close homologs of PCA2 in any of the HMP stool samples collected from 82 subjects, with only a small fraction of stool samples (<8%) containing distant homologs of PCA2 (Fig. 2 panel c). In the case of the gastric bacterial isolate, *F. periodonticum* EAVG_022 (Strauss et al., 2008; Strauss et al., 2011), this isolate is in fact suspected to be a swallowed oral bacterium caught in the act of transiting (personal communication, E. Allen-Vercoe), which would be consistent with *Fusobacteria* being almost exclusively limited to the oral cavity of healthy subjects (Huttenhower et al., 2012). The colon bacterial isolate, on the other hand, does not appear to reflect stool microbiota: *F. nucleatum* EAVG_003 (Strauss et al., 2008; Strauss et al., 2011) was obtained from a healthy individual with careful measures taken to avoid stool contamination (Strauss et al., 2008), and indeed the *Fusobacterium* genus is almost undetectable in fecal samples (Walter et al., 2002). Conversely, *F. nucleatum* has been associated with gastrointestinal pathologies including colorectal cancer (Brennan and Garrett, 2018). Therefore, the colon bacterial isolate positive for PCA2 appears to reflect the microbiota of the colon and not that of stool. Indeed, it has been proposed that bacterial species colonizing the gut lumen are different from those resident on the gut mucosa (Zoetendal et al., 2003; Strauss et al., 2008).

Table S11. Prevalence of markers in metagenomic studies of stool samples obtained from healthy individuals. Prevalence of markers was determined in 14 metagenomic studies included in the Gut Virome Database (GVD) *(Gregory et al., 2020)* investigating stool samples obtained from heathy individuals. The table shows the maximum percent identity at the amino acid level in each study using an alignment length threshold of 150 amino acids (increasing the alignment length threshold to 250 amino acids did not change the results). When no alignment exceeded the alignment length threshold, the percent identity was set to 0. bulk: metagenomic studies of whole microbial communities, VLP: metagenomic studies of virus-like particles. Presence of remote homologs of PCA1 and PCA2 in stool was consistent with results from the HMP study (Fig. 2 panel c).

					TerL marker						
	**Study**	**Age group**	**Health status**	**Country**	**HB1**	**HB2**	**PCA1**	**PCA2**	**HA**	**AB2**	**AB1**
bulk	Han_2018	Adults	Hypertension, Healthy	China	75.8	83.8	51.1	84.3	36.5	31	43.3
bulk	Rampelli_2017	Adults	Healthy	Tanzania, Peru, Italy, USA	75.7	80.9	55.2	64.1	35	28	41.8
bulk + VLP	Shkoporov_2019	Adults	Healthy	Ireland	75.8	80	54.7	58.1	59.6	28	44.1
VLP	Kang_2017	Children, Adults	ASD FMT: donors+recipients, Healthy	USA	97.2	73.9	51.3	46.6	40.7	82	46.3
VLP	Yinda_2019	Infants, Children, Adults, Elderly	Healthy (but with contact with bats)	Cameroon	99.8	66.8	51.4	55.1	42.1	0	28.1
VLP	Minot_2012	Adults	Healthy	USA	76.1	63.5	97	48.8	86.3	30	98.8
VLP	Minot_2013	Adults	Healthy	USA	74.2	62.5	50.6	46.9	85.3	0	24.2
VLP	Moreno-Gallego_2019	Adults	Healthy	UK	76.3	29	34.8	28.6	34	0	23.7
VLP	Minot_2011	Adults	Healthy	USA	72.2	30.1	49.9	33	24.8	0	25.8
VLP	McCann_2018	Infants	Birthmode	Ireland	75	32	33.8	30.3	33.3	35	31.1
VLP	Manrique_2016	Adults	Healthy	USA	75.1	37.8	35.2	47	42.5	28	30.4
VLP	Ly_2016	Adults	Healthy	USA	73.7	32.6	47.7	26.9	29.1	0	25.3
VLP	Lim_2015	Infants	Healthy	USA	74.6	31.6	48.9	26.8	34.6	0	25
VLP	Stockdale_2018	Elderly	Healthy	Ireland	0	25.4	45.9	27.1	23.6	0	0

Percent identity legend

**>70%**
**>55%**
**>50%**
**<50%**

Table S12. Phylogenetic placement of bacterial and phage isolates harboring close homologs of the TerL markers (provided as an excel file). Phylogenetic placement is provided for a representative host of each species provided in Table S10. Columns are as follows: “Source”: source of genome (bacterial or phage), “Origin”: origin of bacterial or phage isolate (e.g., human, animal, environmental, etc.). The following six columns provide a phylogenetic placement of the bacterium in terms of phylum, class, order, family, genus, and species. “Example of strain”: example of a strain harboring a close homolog of the corresponding marker. In the case of phage isolates, a viral classification is provided.

Table S13. Diversity of TerL lineages in individual metagenomes. Overall mean percent identity between indicated TerL markers and all close homologs present in the indicated metagenomes. Collected TerL sequences for each marker were aligned using MUSCLE (Edgar, 2004) in MEGA (Tamura et al., 2013), trimmed to a fixed alignment length, and the mean percent identity was calculated between all pairs of sequences at both the nucleotide (nt) level and the amino acid (aa) level. "No. seqs": number of alleles found for each marker in the given metagenome and included in the final alignment. Also indicated are the alignment length, the body habitat and the medical record number corresponding to each metagenome.

	Overall mean percent identity		Length of alignment (nt)	No. seqs	IMG ID	Habitat	Medical record number (MRN)
	nt	aa					
**HA**	79.3	89.1	546	4	7000000389	Tongue dorsum	160319967
**HB1**	83.8	95.8	456	4	7000000639	Buccal mucosa	764224817
**HB2**	81.6	90.2	945	3	7000000268	Tongue dorsum	160502038
**PCA1**	83.3	92.1	315	3	7000000374	Tongue dorsum	159632143
**PCA2**	84.5	94.8	498	3	7000000727	Tongue dorsum	763577454

Table S14. Statistical evaluation of "P" clades. To evaluate the statistical significance of absence of metagenomic alleles from "P" clades we assumed a null hypothesis wherein any allele in the phylogenetic network can map with equal likelihood to any node in the network. Since "P" clades were strictly devoid of metagenomic alleles from the HMP cohort, we calculated the probability of observing no metagenomic alleles in the "P" clade given the number of human-associated bacterial hosts observed in the "P" clade, and given the total number of alleles from human-associated bacterial hosts and the total number of alleles from the HMP cohort observed in the network, assuming that the null hypothesis is true. The calculation was performed once using an OTU assignment for human-associated bacterial hosts, and once without assigning OTUs. The second part of the table breaks down the number of human-associated bacterial hosts in the given "P" clade into one of three categories based on information provided by the submitting author (see Materials and Methods): (i) hosts that are pathogenic, isolated from a diseased body site/organ or designated as a carriage strain , (ii) hosts isolated from healthy subjects, or (iii) there was insufficient information to classify the host (references for classification of hosts are provided in Table S10).

						Human-associated bacterial hosts in "P" clade		
	HMP metagenomic alleles	OTUs	Human-associated bacterial hosts	Human-associated bacterial hosts in "P" clade	P value	Hosts associated with pathogenicity, disease or carriage	Hosts isolated from healthy subjects	Unknown
**HB2**	83	yes	36	12	5.9E-07			
		no	230	196	0	192	2	2
**HA**	88	yes	22	7	1.3E-05			
		no	134	29	4.4E-07	24	0	5
**PCA1**	87	yes	30	13	2.1E-08			
		no	1800	121	3.3E-03	119	0	2
**PCA2**	57	yes	6	2	9.1E-03			
		no	7	2	1.2E-02	2	0	0
**AB2**	52	yes	5	2	7.7E-03			
		no	7	3	1.7E-03	3	0	0

Table S15. Fraction of TerL gene families in the phageome associated with the TerL markers. The table shows the percent of TerL gene families reported by MCRL for each of the Mira dataset metagenomes that were associated with TerL markers. A viral RefSeq gene encoding a TerL gene was determined to be associated with a TerL marker if its signature contained one or more contigs that yielded at least 70% identity at the amino acid level when aligned against the given TerL marker. For this estimation was used an inclusive overlap condition because this criterion is more effective at removing redundant reference genes compared to a stringent overlap condition (Tadmor and Phillips, 2022) and therefore should provide an unbiased estimation. Columns are as follows: "Metagenome": metagenome identifier from the Mira study, "No. of TerL gene families": number of viral RefSeq genes reported by MCRL for the given metagenome that encode a TerL gene, "No. TerL gene families corresponding to markers": number of viral RefSeq genes reported by MCRL for the given metagenome that encode a TerL gene that were determined to be associated with a TerL marker, "%": percent of TerL gene families reported by MCRL for the given metagenome that were associated with TerL markers, determined based on the previous two columns.

Metagenome	No. TerL gene families	No. TerL gene families corresponding to markers	%	
**HA**	12	4	33.3	
**HB**	13	4	30.8	
**PCA**	14	2	14.3	
**PCB**	14	3	21.4	
**AA**	17	5	29.4	
**AB**	21	4	19	
			**24.7±7.5**	**mean±std**

Table S16. Statistical analysis of potential biases in the prevalence of markers in HMP and environmental metagenomes (provided as an excel file). **a**, HMP metagenomes in Fig. 2 panel c were divided into two groups: metagenomes above and below the median genome size for the given body site. We then calculated the proportion of metagenomes carrying close homologs of the TerL markers (≥70% identity at the amino acid level) for each group and determined the P values for observing these differences by chance. All P values in this table were calculated using a two tailed Z test. **b,** Same analysis as in (a) but for the subset of metagenomes for the corresponding body habitat. **c,** Same analysis as in (a) but applying the statistical analysis to the median contig length of each metagenome. **d,** Statistical evaluation of potential biases in the prevalence of homologs of the TerL markers in the 233 IMG/M environmental metagenome included in Fig. 1 panel g. The first three columns show the prevalence of homologs of TerL markers yielding at least 70% identity at the amino acid level when aligned against the given TerL marker in the oral metagenomes included in Fig. 1 panel f. The next column shows the prevalence of homologs of TerL markers using the same alignment criteria for the 233 IMG/M environmental metagenomes included in Fig. 1 panel g. In the following columns the statistical analysis described in (a) is repeated for the 233 environmental metagenomes for the following metagenomic parameters: genome size, mean contig length, read depth determined by coverage of ribosomal proteins, the number of 5S, 16S and 23S rRNA phylotypes detected by the standard IMG annotation pipeline (Huntemann et al., 2015), and the average number of ribosomal proteins (averaging counts for the following representative pfams: pfam00318, pfam00347, pfam00411, pfam00573, pfam01196). **e,** Same as panel d but restricting the analysis only to metagenomes that exceed the median genome size of oral metagenomes included in Fig. 1 panel f ($7 \cdot 10^7$ nt). **Panels f, g,** Same as panel d but for the subset of environmental metagenomes with an estimated read depth above (f) and below (g) the median read depth. **h**, Same analysis as panel d but using a percent identity threshold of 55% at the amino acid level. **Panels i, j,** same as panels f and g but using a percent identity threshold of 55% at the amino acid level.

Supplementary References

Baker, M., Jiang, W., Rixon, F., and Chiu, W. (2005). Common ancestry of herpesviruses and tailed DNA bacteriophages. *Journal of virology* 79(23)**,** 14967.

Belda-Ferre, P., Alcaraz, L.D., Cabrera-Rubio, R., Romero, H., Simón-Soro, A., Pignatelli, M., et al. (2012). The oral metagenome in health and disease. *The ISME journal* 6(1)**,** 46-56.

Black, L. (1995). DNA packaging and cutting by phage terminases: control in phage T4 by a synaptic mechanism. *Bioessays* 17(12)**,** 1025-1030.

Brennan, C.A., and Garrett, W.S. (2018). *Fusobacterium nucleatum*—symbiont, opportunist and oncobacterium. *Nature Reviews Microbiology***,** 1.

Casjens, S. (2003). Prophages and bacterial genomics: what have we learned so far? *Molecular Microbiology* 49(2)**,** 277-300.

Casjens, S., Gilcrease, E., Winn-Stapley, D., Schicklmaier, P., Schmieger, H., Pedulla, M., et al. (2005). The generalized transducing *Salmonella* bacteriophage ES18: complete genome sequence and DNA packaging strategy. *Journal of bacteriology* 187(3)**,** 1091.

Casjens, S.R. (2008). Diversity among the tailed-bacteriophages that infect the Enterobacteriaceae. *Research in microbiology* 159(5)**,** 340-348.

Catalano, C.E., Cue, D., and Feiss, M. (1995). Virus DNA packaging: the strategy used by phage λ. *Molecular microbiology* 16(6)**,** 1075-1086.

Chai, S., Bravo, A., Lüder, G., Nedlin, A., Trautner, T., and Alonso, J. (1992). Molecular analysis of the Bacillus subtilis bacteriophage SPP 1 region encompassing genes 1 to 6: the products of gene 1 and gene 2 are required for pac cleavage. *Journal of molecular biology* 224(1)**,** 87-102.

Chen, C., Hemme, C., Beleno, J., Shi, Z.J., Ning, D., Qin, Y., et al. (2018a). Oral microbiota of periodontal health and disease and their changes after nonsurgical periodontal therapy. *The ISME journal* 12(5)**,** 1210-1224.

Chen, I.-M.A., Chu, K., Palaniappan, K., Pillay, M., Ratner, A., Huang, J., et al. (2018b). IMG/M v. 5.0: an integrated data management and comparative analysis system for microbial genomes and microbiomes. *Nucleic acids research* 47(D1)**,** D666-D677.

Darriba, D., Taboada, G.L., Doallo, R., and Posada, D. (2011). ProtTest 3: fast selection of best-fit models of protein evolution. *Bioinformatics* 27(8)**,** 1164-1165.

Daw, M., and Falkiner, F. (1996). Bacteriocins: nature, function and structure. *Micron* 27(6)**,** 467-479.

Edgar, R. (2004). MUSCLE: a multiple sequence alignment method with reduced time and space complexity. *BMC bioinformatics* 5(1)**,** 113.

Eppler, K., Wyckoff, E., Goates, J., Parr, R., and Casjens, S. (1991). Nucleotide sequence of the bacteriophage P22 genes required for DNA packaging. *Virology* 183(2)**,** 519-538.

Gregory, A.C., Zablocki, O., Zayed, A.A., Howell, A., Bolduc, B., and Sullivan, M.B. (2020). The gut virome database reveals age-dependent patterns of virome diversity in the human gut. *Cell host & microbe* 28(5)**,** 724-740. e728.

Huntemann, M., Ivanova, N.N., Mavromatis, K., Tripp, H.J., Paez-Espino, D., Palaniappan, K., et al. (2015). The standard operating procedure of the DOE-JGI Microbial Genome Annotation Pipeline (MGAP v. 4). *Standards in genomic sciences* 10**,** 1-6.

Huson, D., and Bryant, D. (2006). Application of phylogenetic networks in evolutionary studies. *Molecular biology and evolution* 23(2)**,** 254.

Huttenhower, C., Gevers, D., Knight, R., Abubucker, S., Badger, J.H., Chinwalla, A.T., et al. (2012). Structure, function and diversity of the healthy human microbiome. *Nature* 486(7402)**,** 207.

Koonin, E., Senkevich, T., and Dolja, V. (2006). The ancient Virus World and evolution of cells. *Biology direct* 1(1), 29.

Lang, A., and Beatty, J. (2000). Genetic analysis of a bacterial genetic exchange element: the gene transfer agent of *Rhodobacter capsulatus*. *Proceedings of the National Academy of Sciences of the United States of America* 97(2), 859.

Lang, A., and Beatty, J. (2007). Importance of widespread gene transfer agent genes in [alpha]-proteobacteria. *Trends in Microbiology* 15(2), 54-62.

Le, S.Q., and Gascuel, O. (2008). An improved general amino acid replacement matrix. *Molecular Biology and Evolution* 25(7), 1307-1320.

Lubbers, M.W., Waterfield, N.R., Beresford, T.P., Le Page, R.W., and Jarvis, A.W. (1995). Sequencing and analysis of the prolate-headed lactococcal bacteriophage c2 genome and identification of the structural genes. *Appl. Environ. Microbiol.* 61(12), 4348-4356.

Marchler-Bauer, A., Bo, Y., Han, L., He, J., Lanczycki, C.J., Lu, S., et al. (2016). CDD/SPARCLE: functional classification of proteins via subfamily domain architectures. *Nucleic acids research* 45(D1), D200-D203.

Martin, D., and Rybicki, E. (2000). RDP: detection of recombination amongst aligned sequences. *Bioinformatics* 16(6), 562.

Methé, B.A., Nelson, K.E., Pop, M., Creasy, H.H., Giglio, M.G., Huttenhower, C., et al. (2012). A framework for human microbiome research. *Nature* 486(7402), 215-221.

Michel-Briand, Y., and Baysse, C. (2002). The pyocins of *Pseudomonas aeruginosa*. *Biochimie* 84(5-6), 499-510.

Mitchell, M., Matsuzaki, S., Imai, S., and Rao, V. (2002). Sequence analysis of bacteriophage T4 DNA packaging/terminase genes 16 and 17 reveals a common ATPase center in the large subunit of viral terminases. *Nucleic Acids Research* 30(18), 4009.

Moore, S.D., and Prevelige Jr, P.E. (2002). DNA packaging: a new class of molecular motors. *Current Biology* 12(3), R96-R98.

Nakayama, K., Takashima, K., Ishihara, H., Shinomiya, T., Kageyama, M., Kanaya, S., et al. (2000). The R-type pyocin of *Pseudomonas aeruginosa* is related to P2 phage, and the F-type is related to lambda phage. *Molecular Microbiology* 38(2), 213-231.

Nawrocki, E.P., and Eddy, S.R. (2013). Infernal 1.1: 100-fold faster RNA homology searches. *Bioinformatics* 29(22), 2933-2935.

Padidam, M., Sawyer, S., and Fauquet, C. (1999). Possible emergence of new geminiviruses by frequent recombination. *Virology* 265(2), 218-225.

Paez-Espino, D., Chen, I.-M.A., Palaniappan, K., Ratner, A., Chu, K., Szeto, E., et al. (2016). IMG/VR: a database of cultured and uncultured DNA Viruses and retroviruses. *Nucleic acids research*, gkw1030.

Rao, V.B., and Feiss, M. (2008). The bacteriophage DNA packaging motor. *Annual Review of Genetics* 42, 647-681.

Schmidt, H., Strimmer, K., Vingron, M., and Von Haeseler, A. (2002). TREE-PUZZLE: maximum likelihood phylogenetic analysis using quartets and parallel computing. *Bioinformatics* 18(3), 502.

Smith, J. (1992). Analyzing the mosaic structure of genes. *Journal of molecular evolution* 34(2), 126-129.

Strauss, J., Kaplan, G.G., Beck, P.L., Rioux, K., Panaccione, R., DeVinney, R., et al. (2011). Invasive potential of gut mucosa-derived fusobacterium nucleatum positively correlates with IBD status of the host. *Inflammatory Bowel Diseases* 17(9), 1971-1978.

Strauss, J., White, A., Ambrose, C., McDonald, J., and Allen-Vercoe, E. (2008). Phenotypic and genotypic analyses of clinical< i> Fusobacterium nucleatum</i> and< i> Fusobacterium periodonticum</i> isolates from the human gut. *Anaerobe* 14(6), 301-309.

Strimmer, K., and Von Haeseler, A. (1997). Likelihood-mapping: a simple method to visualize phylogenetic content of a sequence alignment. *Proceedings of the National Academy of Sciences* 94(13)**,** 6815.

Sun, S., Kondabagil, K., Draper, B., Alam, T., Bowman, V., Zhang, Z., et al. (2008). The structure of the phage T4 DNA packaging motor suggests a mechanism dependent on electrostatic forces. *Cell* 135(7)**,** 1251-1262.

Tadmor, A.D., Ottesen, E.A., Leadbetter, J.R., and Phillips, R. (2011). Probing individual environmental bacteria for viruses by using microfluidic digital PCR. *Science* 333(6038)**,** 58-62.

Tadmor, A.D., and Phillips, R. (2022). MCRL: using a reference library to compress a metagenome into a non-redundant list of sequences, considering viruses as a case study. *Bioinformatics* 38(3)**,** 631-647.

Tamura, K., Stecher, G., Peterson, D., Filipski, A., and Kumar, S. (2013). MEGA6: Molecular Evolutionary Genetics Analysis Version 6.0. *Molecular Biology and Evolution* 30(12)**,** 2725-2729.

Ventura, M., Canchaya, C., Pridmore, R.D., and Brüssow, H. (2004). The prophages of *Lactobacillus johnsonii* NCC 533: comparative genomics and transcription analysis. *Virology* 320(2)**,** 229-242.

Walter, J., Margosch, D., Hammes, W.P., and Hertel, C. (2002). Detection of Fusobacterium species in human feces using genus-specific PCR primers and denaturing gradient gel Electrophoresis. *Microbial ecology in health and disease* 14(3)**,** 129-132.

Whelan, S., and Goldman, N. (2001). A general empirical model of protein evolution derived from multiple protein families using a maximum-likelihood approach. *Molecular Biology and Evolution* 18(5)**,** 691-699.

Wommack, K.E., Bhavsar, J., Polson, S.W., Chen, J., Dumas, M., Srinivasiah, S., et al. (2012). VIROME: a standard operating procedure for analysis of viral metagenome sequences. *Standards in genomic sciences* 6(3)**,** 421.

Yang, Z. (2007). PAML 4: phylogenetic analysis by maximum likelihood. *Molecular biology and evolution* 24(8)**,** 1586-1591.

Yasbin, R.E., and Young, F.E. (1974). Transduction in *Bacillus subtilis* by Bacteriophage SPP1. *Journal of virology* 14(6)**,** 1343-1348.

Zoetendal, E., Plugge, C., Akkermans, A., and de Vos, W. (2003). Victivallis vadensis gen. nov., sp. nov., a sugar-fermenting anaerobe from human faeces. *International journal of systematic and evolutionary microbiology* 53(1)**,** 211.
